# Effects of Treatment Delay on Efficacy of Tecovirimat Following Lethal Aerosol Monkeypox Virus Challenge in Cynomolgus Macaques

**DOI:** 10.1093/infdis/jiy326

**Published:** 2018-07-05

**Authors:** Andrew T Russo, Douglas W Grosenbach, Trevor L Brasel, Robert O Baker, Andrew G Cawthon, Erin Reynolds, Tara Bailey, Philip J Kuehl, Victoria Sugita, Krystle Agans, Dennis E Hruby

**Affiliations:** 1 Lovelace Respiratory Research Institute, Albuquerque, New Mexico; 2 Poxvirus Research Group, SIGA Technologies, Inc, Corvallis, Oregon; 3 Sealy Center for Vaccine Development, University of Texas Medical Branch, Galveston; 4 Microbiology and Molecular Biology Division, Illinois Institute of Technology Research Institute, Chicago; 5 Bacteriology, Virology and In Vitro Operations, Battelle Memorial Institute, Columbus, Ohio; 6 Department of Pathology, University of Texas Medical Branch, Galveston; 7 Covance Laboratories, Madison, Wisconsin; 8 University of New Mexico, Albuquerque; 9 Department of Microbiology and Immunology, University of Texas Medical Branch, Galveston; 10 SIGA Technologies, Inc, Corvallis, Oregon

**Keywords:** smallpox, tecovirimat, monkeypox virus, antiviral, ST-246

## Abstract

**Background:**

Tecovirimat (ST-246) is being developed as an antiviral therapeutic for smallpox for use in the event of an accidental or intentional release. The last reported case of smallpox was 1978 but the potential for use of variola virus for biowarfare has renewed interest in smallpox antiviral therapeutics.

**Methods:**

Cynomolgus macaques were challenged with a lethal dose of monkeypox virus (MPXV) by aerosol as a model for human smallpox and treated orally with 10 mg/kg tecovirimat once daily starting up to 8 days following challenge. Monkeys were monitored for survival, lesions, and clinical signs of disease. Samples were collected for measurement of viremia by quantitative real-time polymerase chain reaction, and for white blood cell counts.

**Results:**

Survival in animals initiating treatment up to 5 days postchallenge was 100%. In animals treated starting 6, 7, or 8 days following challenge, survival was 67%, 100%, and 50%, respectively. Treatment initiation up to 4 days following challenge reduced severity of clinical manifestations of infection.

**Conclusions:**

Tecovirimat treatment initiated up to 8 days following a lethal aerosol MPXV challenge improves survival and, when initiated earlier than 5 days after challenge, provides protection from clinical effects of disease, supporting the conclusion that it is a promising smallpox antiviral therapeutic candidate.

## Teaser

Oral administration of tecovirimat at 10 mg/kg to cynomolgus macaques following a lethal aerosol challenge with MPXV strain Zaire 79 significantly improves survival when treatment is initiated up to 7 days following challenge.

Tecovirimat is a small molecule antiviral drug, with activity against multiple *Orthopoxvirus* species, being developed as a therapeutic for smallpox in the event of a variola virus (VARV) outbreak [1, 2]. Naturally occurring smallpox has not been recorded since 1977 [3], and the last known cases were the result of a laboratory accident in 1978 [4]. Smallpox was officially declared eradicated worldwide and immunization was discontinued in 1980 [5, 6], but the potential for use of VARV for biowarfare or bioterrorism has renewed interest in smallpox antiviral therapeutics [7]. Postexposure prophylaxis using the smallpox vaccine is estimated to be 80%–93% effective in preventing disease if administered within 3 days of natural exposure to VARV [8] but loses efficacy rapidly thereafter and is ineffective after the appearance of clinical signs of smallpox [9, 10]. Also, the safety of the currently licensed smallpox vaccine is a concern [11, 12]. There is no US Food and Drug Administration (FDA)–approved drug for the treatment of symptomatic smallpox that could be used in an outbreak or intentional release of *Orthopoxvirus* into the population.

Tecovirimat targets the VARV p37 protein (C17L gene product), and its homologs in other orthopoxviruses [13], that is required for formation and release of enveloped virions, a form associated with increased virulence of poxviruses [14]. Preclinical studies showed that tecovirimat was highly protective against lethal challenge with all orthopoxviruses evaluated, including cowpox virus, ectromelia virus, and vaccinia virus in mice [15], monkeypox virus (MPXV) and variola virus [16] in nonhuman primates (NHPs) [17], and rabbitpox virus in rabbits [18], via different challenge routes including intravenous, intranasal, and aerosol. Protection could be achieved when treatment was initiated as late as 72 hours postinfection in small animals [13, 15, 18], and 5 days postinfection in NHPs using the intravenous challenge model [15, 17].

Smallpox was one of the most devastating diseases of human history [19]. VARV is highly contagious and can cause severe disease and death in humans. Monkeypox in humans, which resembles smallpox, is a less virulent emerging zoonosis caused by MPXV [20]. The deliberate release of either pathogen in a bioterrorist attack is considered possible by the Centers for Disease Control and Prevention [21] and VARV is considered a material threat to national security by the Department of Homeland Security [22]. Human testing with either VARV or MPXV would be unethical; therefore, therapeutic efficacy studies of tecovirimat must be performed in “well-characterized” animal models according to the FDA [23]. In published studies of MPXV aerosol challenge in NHPs, presented doses from 1.0 × 10^4^ to 1.4 × 10^5^ plaque-forming units (PFU) resulted in expected disease progression and 67%–100% lethality [24, 25]. Peak disease, between 5 and 10 days following challenge, was consistently observed. In addition, a sharp increase in total white blood cell (WBC) counts has been defined as a key endpoint in the model. A lethal aerosol challenge model is useful as this mimics natural exposure routes. Here we report the results of studies to evaluate the ability of tecovirimat to affect disease outcome when administered for 14 days starting 1 to 8 days following lethal aerosol MPXV challenge in cynomolgus macaques.

## MATERIALS AND METHODS

### Animals

Sixty-two cynomolgus macaques (31 males/31 females) between 2 and 7 years of age were obtained from Covance or SNBL USA Ltd. The studies were approved by the Institutional Animal Care and Use Committee of the Lovelace Respiratory Research Institute (LRRI), which is accredited by the Association for Assessment and Accreditation of Laboratory Animal Care, and were conducted in the Animal Biosafety Level 3–enhanced facility at LRRI.

### Challenge Virus

MPXV strain Zaire-V79-I-005 is considered the most well-characterized strain for use in regulated studies and is recommended by the FDA for evaluation of smallpox antivirals. Working stocks were obtained from BEI Resources and were used as received. The reported titer from the BEI Resources Certificate of Analysis was verified by plaque assay on Vero-E6 cells.

Virus aerosol challenge material was prepared individually for each animal on the day of challenge. Working stock was diluted in generator medium (Dulbecco’s modified Eagle medium/2% fetal bovine serum) to a target concentration of 2.5 × 10^7^ PFU/mL and stored on wet ice through the duration of the exposures.

### Aerosol Challenge

Exposures were performed in multiple cohorts over 6 days. Animals were anesthetized with Telazol (2–6 mg/kg intramuscularly) prior to exposure to a target presented dose of 1.0 × 10^5^ PFU MPXV in a head-only chamber housed in a Class 3 Biosafety cabinet. Respiratory parameters of animals were continuously monitored by real-time plethysmography (emka Technologies). Aerosols were generated using a Collison 3-jet nebulizer (BGI Inc). Particle size (1.2–2.6 µm Mass Median Aerodynamic Diameter) was determined using a GRIMM Aerosol Spectrometer (GRIMM Technologies).

Aerosol samples from the exposure chamber were collected into an all-glass impinger (AGI-4, Ace Glass) containing Generator Medium plus antifoam A for MPXV quantification by plaque assay. Presented doses were calculated for each animal based on the plaque assay for each animal’s exposure and the individual total inhaled volume and ranged from 1.83 × 10^4^ to 3.23 × 10^5^ PFU (geometric mean = 6.52 × 10^4^).

### Tecovirimat Treatment

Eleven treatment groups were included to evaluate 8 treatment regimens and untreated controls (Table 1). Animals were administered tecovirimat or vehicle (placebo) by gavage in a volume of 1.0 (± 0.1) mL/kg body weight. Tecovirimat or vehicle delivery was followed by meloxicam (0.3 ± 0.03 mg/kg), then hydrated monkey chow (5 ± 0.5 mL/kg; 6–8 dry biscuits blended with 300–400 mL of tap water).

**Table 1.** Experimental Design, Antiviral Efficacy: Monkeypox Virus Zaire 79 Challenge and Antiviral Treatment

Designation	Test Article Treatment Dose, mg/kg	Animals/Group (Target)		Target Challenge Dose (PFU)
		Male	Female	
Placebo-A	NA	2	2	1.0 × 10^5^
Treatment day 1	10	2	2	1.0 × 10^5^
Treatment day 2	10	3	3	1.0 × 10^5^
Treatment day 3	10	4	4	1.0 × 10^5^
Treatment day 4-A	10	4	4	1.0 × 10^5^
Placebo-B	NA	2	2	1.0 × 10^5^
Treatment day 4-B	10	2	2	1.0 × 10^5^
Treatment day 5	10	3	3	1.0 × 10^5^
Treatment day 6	10	3	3	1.0 × 10^5^
Treatment day 7	10	3	3	1.0 × 10^5^
Treatment day 8	10	3	3	1.0 × 10^5^

Abbreviations: NA, not applicable; PFU, plaque-forming units.

### Observations and Sample Collection

Animals were examined twice daily for up to 45 days for clinical signs of infection, body weight was measured once daily, and lesion counts and blood collection for viral load and hematology analysis were performed as described in the relevant figures. Whole blood (0.5 mL) for hematology and pathogen load analysis was collected from the femoral vein into ethylenediaminetetraacetic acid tubes and stored at 4°C until processed or analyzed. Additional lesion counts and blood collections were performed if euthanasia was required outside the specified sample collection schedule. Animals found moribund were killed. Moribund animals were defined as those demonstrating involuntary movements, respiratory distress or severe dyspnea, persistent recumbency and weakness, unresponsiveness to touch or external stimuli, or body weight loss >20% over a 7-day period.

### Hematology

Hematology parameters were measured using an Advia120 Hematology System (Bayer Corporation).

### DNA Isolation and Quantitative Real-time Polymerase Chain Reaction

Total DNA was isolated from whole blood using DNeasy Blood and Tissue Kit (Qiagen), and MPXV genome quantification was performed as previously described [26]. Extracted DNA was analyzed using the Applied Biosystems 7300 real-time polymerase chain reaction (PCR) system as described previously [26]. Analysis was specific for the pan-*Orthopoxvirus* conserved hemagglutinin (HA) gene. Primers and probes were obtained from BEI Resources (product number NR-9351). To quantify samples, a standard curve was prepared using DNA containing the conserved HA J7R gene segment (GenBank accession number L22579) at known copy number concentrations. The lower limit of quantitation (LLOQ) was 1000 copies/mL blood.

### Statistical Analysis

Statistical analysis was performed by Alpha StatConsult. Methods and results are provided in the Supplementary Materials.

## RESULTS

### Survival

**Figure 1.**
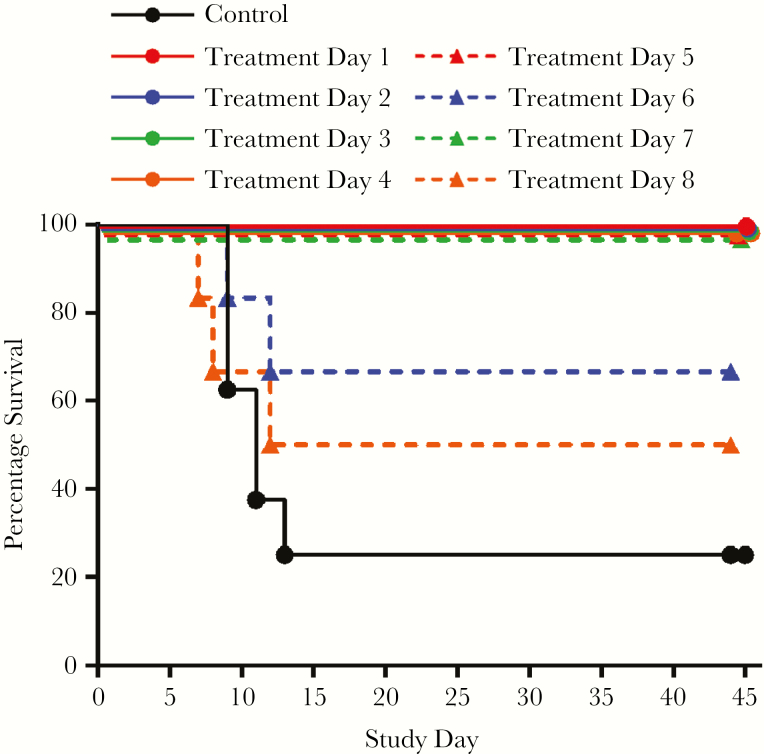
Kaplan–Meier survival analysis of monkeypox virus (MPXV) aerosol challenge by delay of treatment initiation following challenge. Cynomolgus macaques were challenged with MPXV Zaire 79 by the aerosol route and then treated with tecovirimat once daily by the oral route. The day treatment was initiated following challenge is indicated in the key. The effect of tecovirimat treatment on survival was statistically significant (log-rank test, *P* < .05) for study groups initiating tecovirimat from 1 to 7 days following lethal aerosol challenge with MPXV.

Kaplan–Meier plots are presented in Figure 1. Of 54 animals assigned to tecovirimat treatment, 49 survived (90.7%). Of 8 placebo animals, 2 survived (25%). For animals initiating treatment 1–5 and 7 days following challenge, 100% survival was observed. Of animals initiating treatment on day 6, 66% survived, and of animals initiating treatment on day 8, 50% survived. Protective efficacy of tecovirimat was statistically significant for groups that initiated treatment up to day 7. Two animals scheduled to initiate treatment on day 8 were declared moribund and were killed prior to initiation of treatment. One other was declared moribund and was killed 12 days following challenge after receiving 4 tecovirimat doses, making the survival rate 75% for remaining animals that initiated treatment on day 8.

### Body Weight

**Figure 2.**
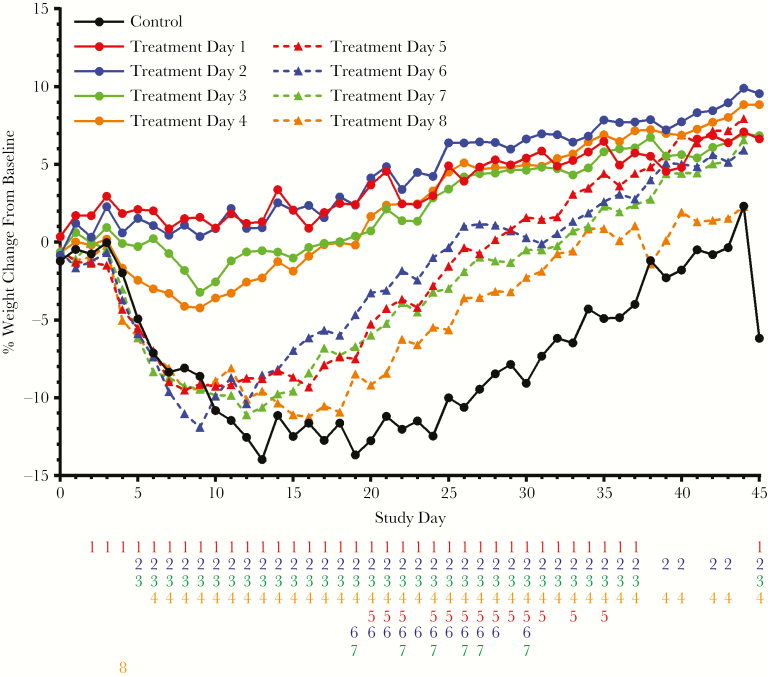
Group mean daily percentage weight change by interval between monkeypox virus challenge and initiation of treatment. Number columns below the x-axis indicate statistically significant differences (determined using analysis of variance; *P* < .05) in the fractional weight loss between the untreated placebo group and the groups initiating treatment on the indicated day following challenge (treatment days 1–8). If a number is present, then there was a significant difference between the group mean body weight change of the indicated treatment group and the placebo group. Numbers are color-matched to the treatment groups (treatment days 1 and 5, red; treatment days 2 and 6, blue; treatment days 3 and 7, green; treatment days 4 and 8, orange).

Mean body weight change for each treatment group is shown in Figure 2. Mean body weight increased in all treated groups by the study conclusion. Mean body weight in the placebo group decreased starting 5 days after challenge and did not return to baseline until day 38. The decreased weight loss observed in groups that initiated treatment from 1 to 4 days following challenge was statistically significant over most of the study. Weight loss in groups that initiated treatment later than day 4 was not significantly different from the placebo results until later in the study due to slower recovery of lost weight in the placebo group. Regardless of the delay between challenge and initiation of treatment, all groups starting treatment 4 or more days following challenge continued to lose weight until at least day 8, and later treatment initiation was associated with increased maximum weight loss. All surviving animals gained additional weight relative to starting weight by the study conclusion.

### Clinical Scores

**Figure 3.**
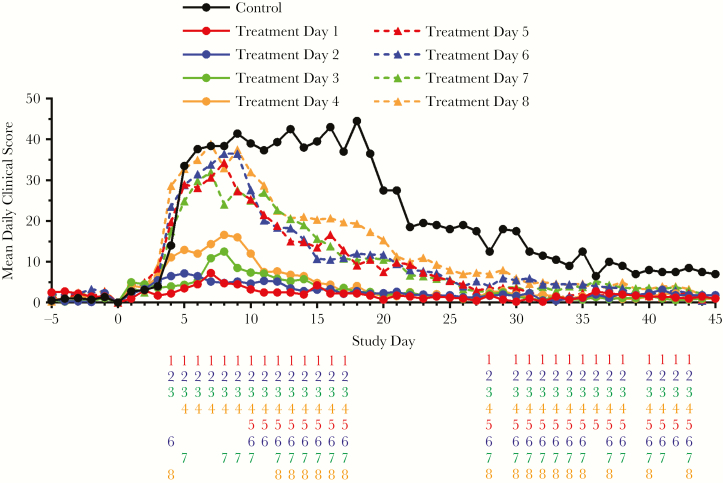
Mean daily clinical observation scores following lethal aerosol challenge with monkeypox virus (MPXV). Detailed clinical observations including temperature, respiratory characteristics, neurological signs, appetite, body weight, appearance, natural cage behavior, provoked (chair) behavior, and gastrointestinal/urogenital characteristics were monitored and documented. Recorded observations were assigned numerical values as indicated below, were compiled into quantitative scores, and the group mean score plotted on each study day. Observations fell into 4 severity categories. Category 1 observations were assigned a value of 1 and included dry cough, mild lethargy, reduced grooming, and thin appearance. Category 2 observations were assigned a value of 2 and included rapid respiration, ataxia, minimal urine output, and sustained piloerection. Category 3 observations were assigned a value of 3 and included dyspnea and extended anorexia, among others. Category 4 observations were assigned a value of 4 and included gasping, hunched or prostrate posture, and unresponsiveness. Results for placebo subjects (solid black line) from day 13 onward represent 2 survivors. Number columns below the x-axis indicate statistically significant differences (determined using analysis of variance; *P* < .05) in the group mean clinical score between the untreated placebo group and the groups initiating treatment on the indicated day following MPXV challenge (treatment day 1–8). If a number is present, then there was a significant difference between the indicated treatment group and the placebo group. Numbers are color-matched to the treatment groups (treatment days 1 and 5, red; treatment days 2 and 6, blue; treatment days 3 and 7, green; treatment days 4 and 8, orange).

Normalized daily cumulative clinical observation scores are presented in Figure 3. In placebo animals, clinical scores increased rapidly following exposure and reached a broad peak between 5 and 19 days after challenge. The variety and severity of observed signs increased as infection progressed and recovery was slow in survivors. Clinical disease was associated with respiratory distress including coughing/sneezing/nasal discharge, dyspnea, diaphragmatic breathing, increased respiratory rate, and respiratory failure. Reduced grooming, reduced appetite, scant or no stool, minimal urine output, hunched posture, and lethargy were also observed.

Scores for animals initiating treatment on day 1 or 2 were very low and quickly resolved to near prechallenge levels. Scores for groups starting treatment on day 3 or 4 were higher than for groups that started treatment earlier. Clinical scores in these groups peaked around day 8. Significant differences between the treated and placebo group are indicated daily in the figure. Groups starting treatment earlier show significant protection from the effects of disease, including during the peak of illness. Animals starting treatment later show significant improvement in clinical scores later in the course of disease due to earlier recovery than placebo animals.

Peak clinical scores for groups initiating tecovirimat treatment 5 or more days following challenge were higher than the other groups and were similar regardless of whether treatment started on day 5, 6, 7, or 8.

### Viral Load in Blood

**Figure 4.**
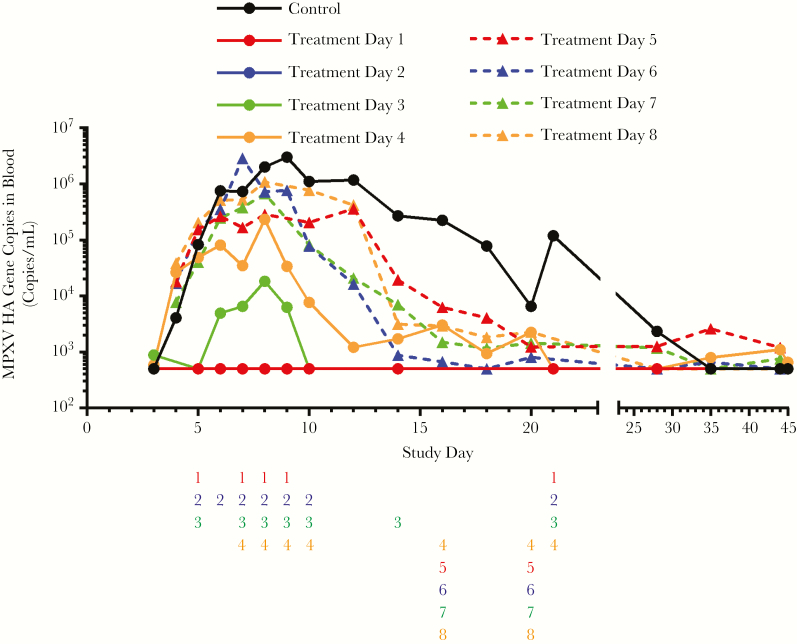
Quantitative real-time polymerase chain reaction results from blood, following monkeypox virus (MPXV) aerosol challenge, for each treatment group. In subjects initiating treatment with tecovirimat starting 1–3 days following challenge, samples were collected prior to day 0 and on days 3, 5, 6, 7, 8, 9, 10, 14, 17, 24, 35, and 45. In subjects initiating treatment with tecovirimat 5–8 days following challenge, samples were collected prior to day zero and on days 4, 5, 6, 7, 8, 9, 10, 12, 14, 16, 18, 20, 22, 30, 35, and 44. The placebo group consists of 4 subjects from each of the sample collection schedules described (8 subjects total), and the group that initiated tecovirimat treatment on day 4 consists of 8 subjects starting sample collection on day 3 and 4 subjects starting sample collection on day 4 (12 subjects total). Number columns below the x-axis indicate statistically significant differences (determined using Kruskal–Wallis test; *P* < .05) in group mean hemagglutinin (HA) gene copies between the untreated placebo group and the groups initiating treatment on the indicated day following MPXV challenge (treatment day 1–8). If a number is present, then there was a significant difference between the indicated treatment group and the placebo group. Numbers are color-matched to the treatment groups (treatment days 1 and 5, red; treatment days 2 and 6, blue; treatment days 3 and 7, green; treatment days 4 and 8, orange).

Group geometric mean viral load is shown in Figure 4. In the placebo group, virus was detected in blood by quantitative real-time PCR (qPCR) as soon as day 4 and was noted in all animals by day 5. In animals that succumbed to infection, viral load generally increased until animals died or were humanely euthanatized. In survivors, viral load peaked on approximately day 9 and then decreased to below the LLOQ prior to the study conclusion. In groups initiating treatment on day 1 or 2, qPCR measurements never exceeded the LLOQ. In the day 3 treatment group, viremia was measurable 3 days following challenge and dropped below the LLOQ by day 10. The day 4 treatment group was viremic between days 5 and 21. Viral DNA was detectable in 2 animals from this group at the conclusion of the study.

Virus was detected by day 5 in all 24 animals that initiated treatment between 5 and 8 days following challenge. Peak viremia observed in these groups was generally between 10^5^ and 10^6^ copies/mL, with some individual animals showing counts as high as 10^7^ (comparable to peak viremia in placebo animals). Viral load remained elevated until death in nonsurvivors. Viremia generally began to decrease by day 10 and fell more slowly than in groups starting treatment earlier, with measurable viremia in some animals until the study conclusion.

### Lesions

**Figure 5.**
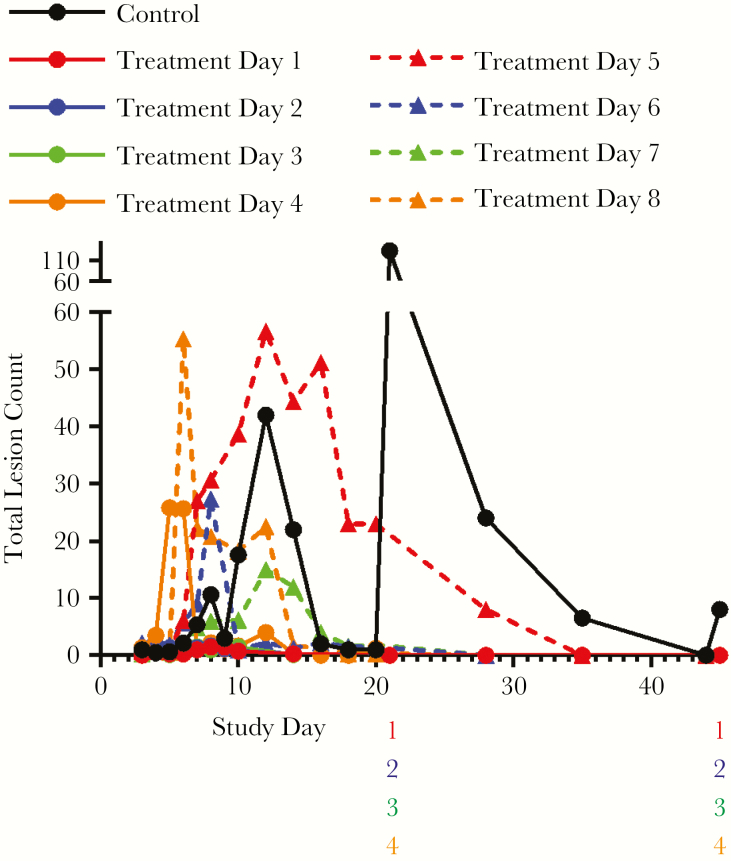
Pox lesion enumeration. Average number of lesions per animal by treatment group following monkeypox virus (MPXV) challenge. The spike in group mean lesion counts in the placebo group is due to results from a single subject in which lesion development appeared delayed relative to the other survivor in the group. Lesions were counted on the same schedule as described for quantitative real-time polymerase chain reaction blood sample collection. Number columns below the x-axis indicate statistically significant differences (determined using Kruskal–Wallis test; *P* < .05) in group mean lesion counts between the untreated placebo group and the groups initiating treatment on the indicated day following MPXV challenge (treatment day 1–8). If a number is present, then there was a significant difference between the indicated treatment group and the placebo group. Numbers are color-matched to the treatment groups (treatment days 1 and 5, red; treatment days 2 and 6, blue; treatment days 3 and 7, green; treatment days 4 and 8, orange).

Group mean total lesion counts are presented in Figure 5. Individual animal lesion counts varied considerably from 1 to >200. Lesions were observed on most moribund and found dead animals, but counts did not typically peak until around 12 days following challenge.

Lesions were initially recorded on day 2 in all groups, except the day 1 treatment group. In the placebo group, lesions were observed in all animals and counts increased until animals died. Peak counts in the 2 placebo group survivors were 2 and 150, and ranged between 1 and 87 in animals that succumbed to disease.

In all treated groups, mean counts peaked between days 7 and 12 and lesions resolved in survivors by the study conclusion. Only early-stage lesions were observed in the day 1 treatment group. In the rest of the treatment groups, all lesion stages were observed. Total lesion counts, progression, and maturation appear to be affected by the day of treatment initiation up to day 4, but the observed differences are not statistically significant.

### Hematology

#### Total White Cells

**Figure 6.**
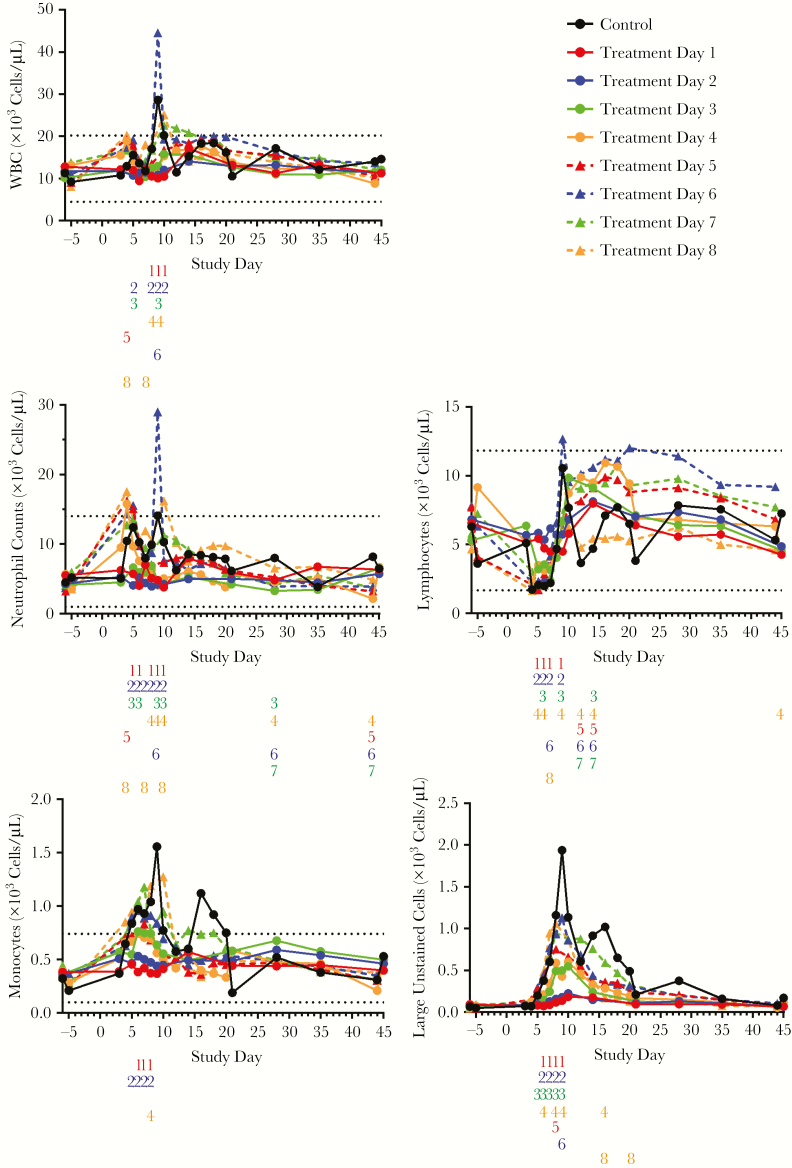
Average cell counts by interval between monkeypox virus (MPXV) challenge and initiation of treatment with tecovirimat. With respect to normal ranges, 2 sources of data were used: mean values compiled from the literature [27–32] and average results compiled from prior studies performed at Lovelace Respiratory Research Institute. Normal ranges are indicated in the figures by horizontal dashed lines. Number columns below the x-axis indicate statistically significant differences (determined using analysis of variance and Kruskal–Wallis test; *P* < .05) in group mean white blood cell (WBC) counts between the untreated placebo group and the groups initiating treatment on the indicated day following MPXV challenge (treatment day 1–8). If a number is present, then there was a significant difference between the indicated treatment group and the placebo group. Numbers are color-matched to the treatment groups (treatment days 1 and 5, red; treatment days 2 and 6, blue; treatment days 3 and 7, green; treatment days 4 and 8, orange).

Increases in group mean total WBC counts were observed in as early as day 4 in placebo and late treatment initiation groups (Figure 6) and in all study groups by day 14. Significant differences in WBC counts between treated groups and placebo were observed before day 10, especially in groups starting treatment before day 5. In the placebo group, and groups that initiated treatment on day 6 or later, peak counts exceeded the normal range described in the literature [27] or obtained by LRRI. Counts in all surviving animals were within normal range by the end of the study.

#### Neutrophils

The placebo group and groups that initiated treatment on or after day 4 showed increased mean counts between days 4 and 5, followed by a decrease until day 7 or 8 and a second peak around day 9 or 10. Significant differences in counts between treated and placebo groups were observed before day 10, especially in groups starting treatment before day 5. Mean counts in groups that initiated treatment on or before day 3 showed negligible changes. Mean counts for survivors in all groups returned to near prechallenge levels by the study conclusion.

#### Lymphocytes

Increased mean lymphocyte counts were observed in all study groups following challenge. Peaks were observed between day 9 and 15, with significant differences observed between the placebo group and most of the treatment groups on multiple days. Group mean counts fell rapidly in the placebo group as animals died, and were dispersed widely within the physiological range in the survivors.

All groups starting treatment on or after day 3 showed reduced group mean counts by day 4. After day 7, all groups showed increased mean counts that generally correlated with delay of treatment initiation with the exception of animals that initiated treatment on day 8, which showed a weak response compared to that observed for the other groups. Group mean counts in all groups were within the normal range by the conclusion of the study.

#### Monocytes

Group mean counts increased by day 4 in the placebo group and all groups that initiated tecovirimat treatment on day 3 or later and peaked above the normal range. Peak counts were usually higher for the placebo group and the groups that initiated treatment later. Lower counts observed in groups starting treatment on day 1, 2, and 4 were statistically significant. Counts returned to normal values by day 12 except for the day 7 group, which remained elevated until day 18.

#### Large Unstained Cells

Prior to challenge, large unstained cells (LUCs) were practically absent (<100 cells/µL). LUC counts in the placebo group increased rapidly and rose to higher levels than in the treated groups. Groups initiating treatment on days 1 or 2 showed elevated mean counts starting on day 7 that peaked on day 10. In groups initiating treatment on day 3 or later, counts increased more rapidly than in the other treated groups. Prior to day 10, the lower counts observed in groups starting treatment before day 6 were statistically significant. Elevated counts peaked between days 8 and 10, and returned to prechallenge levels by the conclusion of the study.

## DISCUSSION

Smallpox was declared eradicated and routine immunization was discontinued in 1980 [5, 6]. Since that time concerns have been raised about the possible use of VARV as a weapon [7] and emergence of other orthopoxviruses such as monkeypox and cowpox virus in a human population with waning immunity [33, 34]. Although effective, the vaccine is not approved for use in the general population unless there is a smallpox outbreak, due to a high incidence of vaccine-related adverse events [35–37]. Currently, the only approved medical countermeasures available for use against *Orthopoxvirus* infections in humans are Vaccinia Immune Globulin Intravenous (Human) (Emergent BioSolutions), and ACAM2000 (Sanofi Pasteur); hence, there is a clear unmet need for safe and effective antiviral therapeutics for use against *Orthopoxvirus* diseases. Tecovirimat is being developed as an oral therapeutic for the treatment of symptomatic smallpox in humans.

As smallpox no longer occurs, clinical trials of tecovirimat in human smallpox are impossible [6]. While human monkeypox outbreaks have occurred periodically in central Africa, and once in the United States, incidents are infrequent, and outbreaks occur in regions where well-controlled clinical trials would be challenging to conduct, making evaluation of clinical efficacy difficult. When human efficacy trials are unfeasible it becomes necessary to investigate clinical efficacy in “well characterized” animal models of the agent of interest or closely related agents expected to have predictive value with regard to the effect of treatment in humans. This has required the development of multiple animal models for human smallpox using various *Orthopoxvirus* species delivered by different exposure routes for evaluation of tecovirimat efficacy [2, 13, 15, 16, 18, 38–45]. In each model, dose regimens that effectively reduced or eliminated clinical signs of disease and mortality were identified.

Here we evaluate the efficacy of oral tecovirimat treatment in the lethal MPXV aerosol challenge model in cynomolgus macaques when initiated from 1 to 8 days following challenge, demonstrating significant efficacy in therapeutic and postexposure prophylactic applications. The oral suspension formulation used in this study mimics human dosing and has similar pharmacokinetic properties in animals as the capsule formulation in humans. Treatment as late as 7 days postinfection significantly improved survival, while earlier treatment initiation (prior to day 5 postinfection) significantly reduced the severity of secondary endpoints of the model, such as weight loss, clinical signs, and viremia. While survival results for groups initiating treatment on day 6 or 7 were both statistically improved relative to placebo-treated animals, unexpectedly, treatment initiation on day 7 (100% survival) provided greater protection from mortality than treatment initiation on day 6 (66%) survival. This result may be unintuitive but considering the complexity of the test system, and the severity of disease experienced by animals treated so late in disease, it is not entirely unexpected.

Significant elevation of WBC levels by 4 days following aerosol MPXV challenge, compared to prechallenge baseline, has been observed previously. Increases in neutrophils and decreases in lymphocytes are consistent with an inflammatory response [24]. Similarly, leukocytosis was observed in 45% of human monkeypox cases [46].

We observed clearly elevated WBC counts with neutrophil elevation and lymphocyte depletion following challenge unless treatment was initiated within 3 days. The amplitude of changes in WBC counts was influenced by the delay of treatment initiation with reduced responses when treatment was initiated as late as 3 days following challenge relative to placebo treatment and later treatment groups, suggesting that earlier initiation of tecovirimat treatment mitigates the inflammatory effects of MPXV infection in macaques.

These results demonstrate that tecovirimat treatment provides protection from mortality and clinical signs of disease when administered up to 8 days following lethal aerosol challenge with MPXV and that the protective efficacy is affected by the day of treatment initiation following challenge. This also supports the use of the aerosol challenge model in cynomolgus macaques as an alternate to the more commonly used and better characterized intravenous challenge model that has been used previously to evaluate tecovirimat efficacy.

The demonstrated effectiveness of tecovirimat against all orthopoxviruses tested in vitro (including VARV) and in numerous animal models provides a reasonable expectation of efficacy vs smallpox in humans. Given that human smallpox is unlikely to be diagnosed until after clinical signs of disease are evident, the success of treatment late in disease as demonstrated here is suggestive of efficacy in symptomatic smallpox.

## Supplementary Data

Supplementary materials are available at *The Journal of Infectious Diseases* online. Consisting of data provided by the authors to benefit the reader, the posted materials are not copyedited and are the sole responsibility of the authors, so questions or comments should be addressed to the corresponding author.

Supplementary Material

## Notes

***Acknowledgments.*** We thank Nicola Richardson-Harman, PhD (Alpha StatConsult LLC, Damascus, Maryland) for detailed statistical analysis of the results presented in this manuscript. We also gratefully acknowledge Helen F. Schiltz, PhD, and Judith Hewitt, PhD, for extensive technical and administrative support.

***Financial support.*** This work was supported by the NIAID, NIH (contract number HHSN2722010000171).

***Potential conflicts of interest.*** A. T. R., D. W. G., and D. E. H. are employees of SIGA Technologies, Inc, and hold stock or equity interests in the company. All other authors report no potential conflicts of interest. All authors have submitted the ICMJE Form for Disclosure of Potential Conflicts of Interest. Conflicts that the editors consider relevant to the content of the manuscript have been disclosed.

Presented in part: 10th Annual American Society of Microbiology Biodefense and Emerging Diseases Research Meeting, Washington, D.C., February 2012, Abstract 228; and 25th International Conference on Antiviral Research, Sapporo, Japan, April 2012, Abstract 24.
