# Supplementary material for: Effects of Treatment Delay on Efficacy of Tecovirimat Following Lethal Aerosol Monkeypox Virus Challenge in Cynomolgus Macaques
Source: J Infect Dis. 2018 Jul 5;218(9):1490–9. doi: 10.1093/infdis/jiy326 (PMC6151088; doi:10.1093/infdis/jiy326)
Supplement: Supplementary Material [file jiy326_suppl_supplementary_materials.docx]

# Statistical Analysis Methods

Daily study endpoints were compared between the Control and Treatment Days 1-8 Groups. Endpoint values below the limit of detection (LOD) were imputed with LOD/2. Non-numerical data were excluded from the analysis. Kaplan-Meier survival curves were generated and Dunnett-Hsu adjusted Logrank Chi-Square was used to test for differences in survival probability between Groups. Survival rates were compared between Groups by Stepdown Bonferroni adjusted Fisher Exact test. Study endpoints were tested for normality with the Shapiro-Wilk test on residuals from a one-way ANOVA. When a significant Shapiro-Wilk statistic (*p*^®^ Version 9.4 (Cary, North Carolina) and an alpha level of 0.05 for treatment comparisons.

# Tables of Statistical Analysis Results

| **Day** | **Test** | **Normality p** | **Main p** | **Comparison** | **Pairwise p** | **Significance** |
| --- | --- | --- | --- | --- | --- | --- |
| 3 | ANOVA | 0.0511 | 0.9767 | 1 vs. 0 | 0.9366 | ns |
|  |  |  |  | 2 vs. 0 | 0.7063 | ns |
|  |  |  |  | 3 vs. 0 | 0.6469 | ns |
|  |  |  |  | 4 vs. 0 | 0.5829 | ns |
| 4 | Kruskal-Wallis | 0.0001 | 0.3510 | 4 vs. 0 | 0.4163 | ns |
|  |  |  |  | 5 vs. 0 | 0.7818 | ns |
|  |  |  |  | 6 vs. 0 | 0.7818 | ns |
|  |  |  |  | 7 vs. 0 | 0.7012 | ns |
|  |  |  |  | 8 vs. 0 | 0.4163 | ns |
| 5 | ANOVA | 0.0156 | 0.0027 | 1 vs. 0 | 0.0991 | ns |
|  |  |  |  | 2 vs. 0 | 0.0965 | ns |
|  |  |  |  | 3 vs. 0 | 0.0469 | p<0.05 |
|  |  |  |  | 4 vs. 0 | 0.3603 | ns |
|  |  |  |  | 5 vs. 0 | 0.2195 | ns |
|  |  |  |  | 6 vs. 0 | 0.1518 | ns |
|  |  |  |  | 7 vs. 0 | 0.7723 | ns |
|  |  |  |  | 8 vs. 0 | 0.2008 | ns |
| 6 | ANOVA | 0.0021 | <.0001 | 1 vs. 0 | 0.0020 | p<0.05 |
|  |  |  |  | 2 vs. 0 | 0.0006 | p<0.05 |
|  |  |  |  | 3 vs. 0 | 0.0036 | p<0.05 |
|  |  |  |  | 4 vs. 0 | 0.0228 | p<0.05 |
|  |  |  |  | 5 vs. 0 | 0.6983 | ns |
|  |  |  |  | 6 vs. 0 | 0.4074 | ns |
|  |  |  |  | 7 vs. 0 | 0.4947 | ns |
|  |  |  |  | 8 vs. 0 | 0.2205 | ns |
| 7 | Kruskal-Wallis | 0.0001 | <.0001 | 1 vs. 0 | 0.0383 | p<0.05 |

| **Day** | **Test** | **Normality p** | **Main p** | **Comparison** | **Pairwise p** | **Significance** |
| --- | --- | --- | --- | --- | --- | --- |
|  |  |  |  | 2 vs. 0 | 0.0154 | p<0.05 |
|  |  |  |  | 3 vs. 0 | 0.0370 | p<0.05 |
|  |  |  |  | 4 vs. 0 | 0.3783 | ns |
|  |  |  |  | 5 vs. 0 | 1.0000 | ns |
|  |  |  |  | 6 vs. 0 | 1.0000 | ns |
|  |  |  |  | 7 vs. 0 | 0.6205 | ns |
|  |  |  |  | 8 vs. 0 | 0.6205 | ns |
| 8 | ANOVA | 0.0088 | <.0001 | 1 vs. 0 | <.0001 | p<0.05 |
|  |  |  |  | 2 vs. 0 | <.0001 | p<0.05 |
|  |  |  |  | 3 vs. 0 | 0.0006 | p<0.05 |
|  |  |  |  | 4 vs. 0 | 0.0013 | p<0.05 |
|  |  |  |  | 5 vs. 0 | 0.0445 | p<0.05 |
|  |  |  |  | 6 vs. 0 | 0.2652 | ns |
|  |  |  |  | 7 vs. 0 | 0.2907 | ns |
|  |  |  |  | 8 vs. 0 | 0.6424 | ns |
| 9 | ANOVA | 0.0036 | <.0001 | 1 vs. 0 | <.0001 | p<0.05 |
|  |  |  |  | 2 vs. 0 | <.0001 | p<0.05 |
|  |  |  |  | 3 vs. 0 | <.0001 | p<0.05 |
|  |  |  |  | 4 vs. 0 | <.0001 | p<0.05 |
|  |  |  |  | 6 vs. 0 | 0.0487 | p<0.05 |
| -5 | ANOVA | 0.8698 | 0.3739 | 4 vs. 0 | 0.4239 | ns |
|  |  |  |  | 5 vs. 0 | 0.8686 | ns |
|  |  |  |  | 6 vs. 0 | 0.3441 | ns |
|  |  |  |  | 7 vs. 0 | 0.2225 | ns |
|  |  |  |  | 8 vs. 0 | 0.8686 | ns |
| -6 | ANOVA | 0.0020 | 0.8024 | 1 vs. 0 | 0.4211 | ns |

| **Day** | **Test** | **Normality p** | **Main p** | **Comparison** | **Pairwise p** | **Significance** |
| --- | --- | --- | --- | --- | --- | --- |
|  |  |  |  | 2 vs. 0 | 0.5886 | ns |
|  |  |  |  | 3 vs. 0 | 0.8705 | ns |
|  |  |  |  | 4 vs. 0 | 0.6110 | ns |
|  |  |  |  | 5 vs. 0 | 0.3043 | ns |
|  |  |  |  | 6 vs. 0 | 0.3043 | ns |
|  |  |  |  | 7 vs. 0 | 0.8972 | ns |
|  |  |  |  | 8 vs. 0 | 0.5332 | ns |
| 10 | Kruskal-Wallis | 0.0002 | 0.0030 | 1 vs. 0 | 0.1924 | ns |
|  |  |  |  | 2 vs. 0 | 0.1080 | ns |
|  |  |  |  | 3 vs. 0 | 0.5484 | ns |
|  |  |  |  | 4 vs. 0 | 0.5692 | ns |
|  |  |  |  | 5 vs. 0 | 1.0000 | ns |
|  |  |  |  | 6 vs. 0 | 1.0000 | ns |
|  |  |  |  | 7 vs. 0 | 1.0000 | ns |
|  |  |  |  | 8 vs. 0 | 1.0000 | ns |
| 12 | ANOVA | 0.3933 | 0.4044 | 4 vs. 0 | 0.6189 | ns |
|  |  |  |  | 5 vs. 0 | 0.8624 | ns |
|  |  |  |  | 6 vs. 0 | 0.7769 | ns |
|  |  |  |  | 7 vs. 0 | 0.3446 | ns |
|  |  |  |  | 8 vs. 0 | 0.5839 | ns |
| 14 | Kruskal-Wallis | 0.0001 | 0.0025 | 1 vs. 0 | 0.2653 | ns |
|  |  |  |  | 2 vs. 0 | 0.2653 | ns |
|  |  |  |  | 3 vs. 0 | 0.2530 | ns |
|  |  |  |  | 4 vs. 0 | 0.2263 | ns |
|  |  |  |  | 5 vs. 0 | 0.2653 | ns |
|  |  |  |  | 6 vs. 0 | 0.2653 | ns |

| **Day** | **Test** | **Normality p** | **Main p** | **Comparison** | **Pairwise p** | **Significance** |
| --- | --- | --- | --- | --- | --- | --- |
|  |  |  |  | 7 vs. 0 | 0.3144 | ns |
|  |  |  |  | 8 vs. 0 | 0.2653 | ns |
| 16 | ANOVA | 0.0013 | 0.1790 | 4 vs. 0 | 0.0494 | p<0.05 |
|  |  |  |  | 5 vs. 0 | 0.0700 | ns |
|  |  |  |  | 6 vs. 0 | 0.0642 | ns |
|  |  |  |  | 7 vs. 0 | 0.2190 | ns |
|  |  |  |  | 8 vs. 0 | 0.0299 | p<0.05 |
| 18 | ANOVA | 0.0086 | 0.4641 | 4 vs. 0 | 0.1577 | ns |
|  |  |  |  | 5 vs. 0 | 0.2935 | ns |
|  |  |  |  | 6 vs. 0 | 0.2730 | ns |
|  |  |  |  | 7 vs. 0 | 0.5222 | ns |
|  |  |  |  | 8 vs. 0 | 0.1359 | ns |
| 20 | ANOVA | 0.0198 | 0.1947 | 4 vs. 0 | 0.0729 | ns |
|  |  |  |  | 5 vs. 0 | 0.1083 | ns |
|  |  |  |  | 6 vs. 0 | 0.2643 | ns |
|  |  |  |  | 7 vs. 0 | 0.2934 | ns |
|  |  |  |  | 8 vs. 0 | 0.0386 | p<0.05 |
| 21 | ANOVA | 0.1522 | 0.2843 | 1 vs. 0 | 0.1408 | ns |
|  |  |  |  | 2 vs. 0 | 0.1716 | ns |
|  |  |  |  | 3 vs. 0 | 0.2775 | ns |
|  |  |  |  | 4 vs. 0 | 0.5437 | ns |
| 28 | Kruskal-Wallis | 0.0001 | 0.2086 | 1 vs. 0 | 0.3845 | ns |
|  |  |  |  | 2 vs. 0 | 0.3185 | ns |
|  |  |  |  | 3 vs. 0 | 0.2892 | ns |
|  |  |  |  | 4 vs. 0 | 0.3845 | ns |
|  |  |  |  | 5 vs. 0 | 0.5394 | ns |

| **Day** | **Test** | **Normality p** | **Main p** | **Comparison** | **Pairwise p** | **Significance** |
| --- | --- | --- | --- | --- | --- | --- |
|  |  |  |  | 6 vs. 0 | 0.5394 | ns |
|  |  |  |  | 7 vs. 0 | 0.5394 | ns |
|  |  |  |  | 8 vs. 0 | 0.3845 | ns |
| 35 | Kruskal-Wallis | 0.0001 | 0.5461 | 1 vs. 0 | 0.8246 | ns |
|  |  |  |  | 2 vs. 0 | 0.5829 | ns |
|  |  |  |  | 3 vs. 0 | 0.2846 | ns |
|  |  |  |  | 4 vs. 0 | 0.8246 | ns |
|  |  |  |  | 5 vs. 0 | 0.8246 | ns |
|  |  |  |  | 6 vs. 0 | 1.0000 | ns |
|  |  |  |  | 7 vs. 0 | 1.0000 | ns |
|  |  |  |  | 8 vs. 0 | 0.5829 | ns |
| 44 | ANOVA | 0.1141 | 0.7054 | 4 vs. 0 | 0.6146 | ns |
|  |  |  |  | 5 vs. 0 | 0.8891 | ns |
|  |  |  |  | 6 vs. 0 | 0.6146 | ns |
|  |  |  |  | 7 vs. 0 | 0.9167 | ns |
|  |  |  |  | 8 vs. 0 | 0.8449 | ns |
| 45 | ANOVA | 0.0025 | 0.2425 | 1 vs. 0 | 0.1154 | ns |
|  |  |  |  | 2 vs. 0 | 0.0962 | ns |
|  |  |  |  | 3 vs. 0 | 0.1283 | ns |
|  |  |  |  | 4 vs. 0 | 0.3639 | ns |
| -32 | ANOVA | 0.2976 | 0.7195 | 4 vs. 0 | 0.2568 | ns |
|  |  |  |  | 5 vs. 0 | 0.8190 | ns |
|  |  |  |  | 6 vs. 0 | 0.6500 | ns |
|  |  |  |  | 7 vs. 0 | 0.8190 | ns |
|  |  |  |  | 8 vs. 0 | 0.9087 | ns |
| -33 | ANOVA | 0.9959 | 0.4955 | 4 vs. 0 | 0.8517 | ns |

| **Day** | **Test** | **Normality p** | **Main p** | **Comparison** | **Pairwise p** | **Significance** |
| --- | --- | --- | --- | --- | --- | --- |
|  |  |  |  | 5 vs. 0 | 0.6434 | ns |
|  |  |  |  | 6 vs. 0 | 0.9254 | ns |
|  |  |  |  | 7 vs. 0 | 0.2568 | ns |
|  |  |  |  | 8 vs. 0 | 0.9254 | ns |
| -34 | ANOVA | 0.0208 | 0.8056 | 4 vs. 0 | 0.8018 | ns |
|  |  |  |  | 5 vs. 0 | 0.3603 | ns |
|  |  |  |  | 6 vs. 0 | 0.4958 | ns |
|  |  |  |  | 7 vs. 0 | 0.8844 | ns |
|  |  |  |  | 8 vs. 0 | 0.9036 | ns |

| **Day** | **Test** | **Normality p** | **Main p** | **Comparison** | **Pairwise p** | **Significance** |
| --- | --- | --- | --- | --- | --- | --- |
| 3 | ANOVA | 0.1381 | 0.7428 | 1 vs. 0 | 0.9745 | ns |
|  |  |  |  | 2 vs. 0 | 0.6680 | ns |
|  |  |  |  | 3 vs. 0 | 0.3286 | ns |
|  |  |  |  | 4 vs. 0 | 0.9946 | ns |
| 4 | ANOVA | 0.0020 | 0.2858 | 4 vs. 0 | 0.4468 | ns |
|  |  |  |  | 5 vs. 0 | 0.5826 | ns |
|  |  |  |  | 6 vs. 0 | 0.4311 | ns |
|  |  |  |  | 7 vs. 0 | 0.0598 | ns |
|  |  |  |  | 8 vs. 0 | 0.9091 | ns |
| 5 | ANOVA | 0.0082 | <.0001 | 1 vs. 0 | 0.0001 | p<0.05 |
|  |  |  |  | 2 vs. 0 | <.0001 | p<0.05 |
|  |  |  |  | 3 vs. 0 | 0.0842 | ns |
|  |  |  |  | 4 vs. 0 | 0.0202 | p<0.05 |
|  |  |  |  | 5 vs. 0 | 0.6157 | ns |
|  |  |  |  | 6 vs. 0 | 0.7044 | ns |
|  |  |  |  | 7 vs. 0 | 0.9110 | ns |
|  |  |  |  | 8 vs. 0 | 0.6405 | ns |
| 6 | ANOVA | 0.2591 | <.0001 | 1 vs. 0 | 0.0002 | p<0.05 |
|  |  |  |  | 2 vs. 0 | <.0001 | p<0.05 |
|  |  |  |  | 3 vs. 0 | 0.0077 | p<0.05 |
|  |  |  |  | 4 vs. 0 | 0.0365 | p<0.05 |
|  |  |  |  | 5 vs. 0 | 0.1535 | ns |
|  |  |  |  | 6 vs. 0 | 0.3376 | ns |
|  |  |  |  | 7 vs. 0 | 0.8182 | ns |
|  |  |  |  | 8 vs. 0 | 0.9236 | ns |
| 7 | ANOVA | 0.4499 | 0.0021 | 1 vs. 0 | 0.0204 | p<0.05 |

| **Day** | **Test** | **Normality p** | **Main p** | **Comparison** | **Pairwise p** | **Significance** |
| --- | --- | --- | --- | --- | --- | --- |
|  |  |  |  | 2 vs. 0 | <.0001 | p<0.05 |
|  |  |  |  | 3 vs. 0 | 0.2205 | ns |
|  |  |  |  | 4 vs. 0 | 0.1143 | ns |
|  |  |  |  | 5 vs. 0 | 0.1441 | ns |
|  |  |  |  | 6 vs. 0 | 0.0233 | p<0.05 |
|  |  |  |  | 7 vs. 0 | 0.5739 | ns |
|  |  |  |  | 8 vs. 0 | 0.0262 | p<0.05 |
| 8 | ANOVA | 0.0339 | 0.4878 | 1 vs. 0 | 0.9806 | ns |
|  |  |  |  | 2 vs. 0 | 0.3670 | ns |
|  |  |  |  | 3 vs. 0 | 0.5371 | ns |
|  |  |  |  | 4 vs. 0 | 0.9837 | ns |
|  |  |  |  | 5 vs. 0 | 0.9697 | ns |
|  |  |  |  | 6 vs. 0 | 0.0928 | ns |
|  |  |  |  | 7 vs. 0 | 0.3196 | ns |
|  |  |  |  | 8 vs. 0 | 0.9061 | ns |
| 9 | ANOVA | 0.1982 | 0.0347 | 1 vs. 0 | 0.0070 | p<0.05 |
|  |  |  |  | 2 vs. 0 | 0.0184 | p<0.05 |
|  |  |  |  | 3 vs. 0 | 0.0454 | p<0.05 |
|  |  |  |  | 4 vs. 0 | 0.0441 | p<0.05 |
|  |  |  |  | 6 vs. 0 | 0.5009 | ns |
| -5 | ANOVA | 0.8449 | 0.6146 | 4 vs. 0 | 0.2370 | ns |
|  |  |  |  | 5 vs. 0 | 0.9120 | ns |
|  |  |  |  | 6 vs. 0 | 0.5391 | ns |
|  |  |  |  | 7 vs. 0 | 0.4195 | ns |
|  |  |  |  | 8 vs. 0 | 0.9047 | ns |
| -6 | ANOVA | 0.0325 | 0.5824 | 1 vs. 0 | 0.8830 | ns |

| **Day** | **Test** | **Normality p** | **Main p** | **Comparison** | **Pairwise p** | **Significance** |
| --- | --- | --- | --- | --- | --- | --- |
|  |  |  |  | 2 vs. 0 | 0.7316 | ns |
|  |  |  |  | 3 vs. 0 | 0.4697 | ns |
|  |  |  |  | 4 vs. 0 | 0.4326 | ns |
|  |  |  |  | 5 vs. 0 | 0.4011 | ns |
|  |  |  |  | 6 vs. 0 | 0.3713 | ns |
|  |  |  |  | 7 vs. 0 | 0.7688 | ns |
|  |  |  |  | 8 vs. 0 | 0.3337 | ns |
| 10 | Kruskal-Wallis | 0.0001 | 0.3455 | 1 vs. 0 | 1.0000 | ns |
|  |  |  |  | 2 vs. 0 | 1.0000 | ns |
|  |  |  |  | 3 vs. 0 | 1.0000 | ns |
|  |  |  |  | 4 vs. 0 | 1.0000 | ns |
|  |  |  |  | 5 vs. 0 | 1.0000 | ns |
|  |  |  |  | 6 vs. 0 | 1.0000 | ns |
|  |  |  |  | 7 vs. 0 | 1.0000 | ns |
|  |  |  |  | 8 vs. 0 | 1.0000 | ns |
| 12 | ANOVA | 0.0396 | 0.0046 | 4 vs. 0 | 0.0044 | p<0.05 |
|  |  |  |  | 5 vs. 0 | 0.0230 | p<0.05 |
|  |  |  |  | 6 vs. 0 | 0.0026 | p<0.05 |
|  |  |  |  | 7 vs. 0 | 0.0090 | p<0.05 |
|  |  |  |  | 8 vs. 0 | 0.5713 | ns |
| 14 | ANOVA | 0.5247 | 0.0934 | 1 vs. 0 | 0.1439 | ns |
|  |  |  |  | 2 vs. 0 | 0.1052 | ns |
|  |  |  |  | 3 vs. 0 | 0.0335 | p<0.05 |
|  |  |  |  | 4 vs. 0 | 0.0177 | p<0.05 |
|  |  |  |  | 5 vs. 0 | 0.0390 | p<0.05 |
|  |  |  |  | 6 vs. 0 | 0.0105 | p<0.05 |

| **Day** | **Test** | **Normality p** | **Main p** | **Comparison** | **Pairwise p** | **Significance** |
| --- | --- | --- | --- | --- | --- | --- |
|  |  |  |  | 7 vs. 0 | 0.0252 | p<0.05 |
|  |  |  |  | 8 vs. 0 | 0.7504 | ns |
| 16 | ANOVA | 0.5211 | 0.0212 | 4 vs. 0 | 0.1131 | ns |
|  |  |  |  | 5 vs. 0 | 0.2294 | ns |
|  |  |  |  | 6 vs. 0 | 0.0934 | ns |
|  |  |  |  | 7 vs. 0 | 0.3025 | ns |
|  |  |  |  | 8 vs. 0 | 0.4911 | ns |
| 18 | ANOVA | 0.5492 | 0.1172 | 4 vs. 0 | 0.3372 | ns |
|  |  |  |  | 5 vs. 0 | 0.5041 | ns |
|  |  |  |  | 6 vs. 0 | 0.2714 | ns |
|  |  |  |  | 7 vs. 0 | 0.2911 | ns |
|  |  |  |  | 8 vs. 0 | 0.4976 | ns |
| 20 | ANOVA | 0.6793 | 0.0746 | 4 vs. 0 | 0.3389 | ns |
|  |  |  |  | 5 vs. 0 | 0.4346 | ns |
|  |  |  |  | 6 vs. 0 | 0.0806 | ns |
|  |  |  |  | 7 vs. 0 | 0.3468 | ns |
|  |  |  |  | 8 vs. 0 | 0.6866 | ns |
| 21 | ANOVA | 0.5812 | 0.6197 | 1 vs. 0 | 0.2617 | ns |
|  |  |  |  | 2 vs. 0 | 0.1523 | ns |
|  |  |  |  | 3 vs. 0 | 0.1339 | ns |
|  |  |  |  | 4 vs. 0 | 0.1625 | ns |
| 28 | ANOVA | 0.2964 | 0.0511 | 1 vs. 0 | 0.3676 | ns |
|  |  |  |  | 2 vs. 0 | 0.8366 | ns |
|  |  |  |  | 3 vs. 0 | 0.5344 | ns |
|  |  |  |  | 4 vs. 0 | 0.6367 | ns |
|  |  |  |  | 5 vs. 0 | 0.5863 | ns |

| **Day** | **Test** | **Normality p** | **Main p** | **Comparison** | **Pairwise p** | **Significance** |
| --- | --- | --- | --- | --- | --- | --- |
|  |  |  |  | 6 vs. 0 | 0.1564 | ns |
|  |  |  |  | 7 vs. 0 | 0.4100 | ns |
|  |  |  |  | 8 vs. 0 | 0.5428 | ns |
| 35 | ANOVA | 0.0921 | 0.1829 | 1 vs. 0 | 0.3935 | ns |
|  |  |  |  | 2 vs. 0 | 0.7117 | ns |
|  |  |  |  | 3 vs. 0 | 0.5281 | ns |
|  |  |  |  | 4 vs. 0 | 0.5954 | ns |
|  |  |  |  | 5 vs. 0 | 0.6822 | ns |
|  |  |  |  | 6 vs. 0 | 0.4032 | ns |
|  |  |  |  | 7 vs. 0 | 0.6296 | ns |
|  |  |  |  | 8 vs. 0 | 0.2574 | ns |
| 44 | ANOVA | 0.4098 | 0.1621 | 4 vs. 0 | 0.6965 | ns |
|  |  |  |  | 5 vs. 0 | 0.5398 | ns |
|  |  |  |  | 6 vs. 0 | 0.1364 | ns |
|  |  |  |  | 7 vs. 0 | 0.3312 | ns |
|  |  |  |  | 8 vs. 0 | 0.7808 | ns |
| 45 | ANOVA | 0.0840 | 0.3317 | 1 vs. 0 | 0.0523 | ns |
|  |  |  |  | 2 vs. 0 | 0.1041 | ns |
|  |  |  |  | 3 vs. 0 | 0.0677 | ns |
|  |  |  |  | 4 vs. 0 | 0.0493 | p<0.05 |
| -29 | ANOVA | 1.0000 | 0.1703 |  | . | n/a |
| -30 | ANOVA | 0.2997 | 0.0387 |  | . | n/a |
| -31 | ANOVA | 0.4456 | 0.9393 |  | . | n/a |
| -32 | ANOVA | 0.5122 | 0.4758 | 3 vs. 0 | 0.3852 | ns |
|  |  |  |  | 4 vs. 0 | 0.0934 | ns |
|  |  |  |  | 5 vs. 0 | 0.6143 | ns |

| **Day** | **Test** | **Normality p** | **Main p** | **Comparison** | **Pairwise p** | **Significance** |
| --- | --- | --- | --- | --- | --- | --- |
|  |  |  |  | 6 vs. 0 | 0.8777 | ns |
|  |  |  |  | 7 vs. 0 | 0.5792 | ns |
|  |  |  |  | 8 vs. 0 | 0.2734 | ns |
| -33 | ANOVA | 0.6260 | 0.7761 | 4 vs. 0 | 0.3524 | ns |
|  |  |  |  | 5 vs. 0 | 0.9105 | ns |
|  |  |  |  | 6 vs. 0 | 0.4872 | ns |
|  |  |  |  | 7 vs. 0 | 0.3412 | ns |
|  |  |  |  | 8 vs. 0 | 0.5897 | ns |
| -34 | ANOVA | 0.2847 | 0.8459 | 4 vs. 0 | 0.6392 | ns |
|  |  |  |  | 5 vs. 0 | 0.4214 | ns |
|  |  |  |  | 6 vs. 0 | 0.5419 | ns |
|  |  |  |  | 7 vs. 0 | 0.9604 | ns |
|  |  |  |  | 8 vs. 0 | 0.9226 | ns |

| **Day** | **Test** | **Normality p** | **Main p** | **Comparison** | **Pairwise p** | **Significance** |
| --- | --- | --- | --- | --- | --- | --- |
| 3 | ANOVA | 0.1489 | 0.5186 | 1 vs. 0 | 0.9094 | ns |
|  |  |  |  | 2 vs. 0 | 0.3293 | ns |
|  |  |  |  | 3 vs. 0 | 0.1444 | ns |
|  |  |  |  | 4 vs. 0 | 0.3029 | ns |
| 4 | ANOVA | 0.2457 | 0.6806 | 4 vs. 0 | 0.9313 | ns |
|  |  |  |  | 5 vs. 0 | 0.7021 | ns |
|  |  |  |  | 6 vs. 0 | 0.9039 | ns |
|  |  |  |  | 7 vs. 0 | 0.6337 | ns |
|  |  |  |  | 8 vs. 0 | 0.2057 | ns |
| 5 | ANOVA | 0.0054 | 0.0576 | 1 vs. 0 | 0.0593 | ns |
|  |  |  |  | 2 vs. 0 | 0.0380 | p<0.05 |
|  |  |  |  | 3 vs. 0 | 0.0805 | ns |
|  |  |  |  | 4 vs. 0 | 0.2626 | ns |
|  |  |  |  | 5 vs. 0 | 0.5110 | ns |
|  |  |  |  | 6 vs. 0 | 0.7367 | ns |
|  |  |  |  | 7 vs. 0 | 0.7367 | ns |
|  |  |  |  | 8 vs. 0 | 0.5417 | ns |
| 6 | ANOVA | 0.2804 | 0.0077 | 1 vs. 0 | 0.0023 | p<0.05 |
|  |  |  |  | 2 vs. 0 | 0.0089 | p<0.05 |
|  |  |  |  | 3 vs. 0 | 0.1688 | ns |
|  |  |  |  | 4 vs. 0 | 0.1063 | ns |
|  |  |  |  | 5 vs. 0 | 0.1833 | ns |
|  |  |  |  | 6 vs. 0 | 0.9203 | ns |
|  |  |  |  | 7 vs. 0 | 0.6971 | ns |
|  |  |  |  | 8 vs. 0 | 0.7484 | ns |
| 7 | ANOVA | 0.4587 | 0.0407 | 1 vs. 0 | 0.0253 | p<0.05 |

| **Day** | **Test** | **Normality p** | **Main p** | **Comparison** | **Pairwise p** | **Significance** |
| --- | --- | --- | --- | --- | --- | --- |
|  |  |  |  | 2 vs. 0 | 0.0328 | p<0.05 |
|  |  |  |  | 3 vs. 0 | 0.3209 | ns |
|  |  |  |  | 4 vs. 0 | 0.1804 | ns |
|  |  |  |  | 5 vs. 0 | 0.6109 | ns |
|  |  |  |  | 6 vs. 0 | 0.8253 | ns |
|  |  |  |  | 7 vs. 0 | 0.2093 | ns |
|  |  |  |  | 8 vs. 0 | 0.7990 | ns |
| 8 | ANOVA | 0.0640 | 0.0229 | 1 vs. 0 | 0.0045 | p<0.05 |
|  |  |  |  | 2 vs. 0 | 0.0060 | p<0.05 |
|  |  |  |  | 3 vs. 0 | 0.1112 | ns |
|  |  |  |  | 4 vs. 0 | 0.0454 | p<0.05 |
|  |  |  |  | 5 vs. 0 | 0.0769 | ns |
|  |  |  |  | 6 vs. 0 | 0.5163 | ns |
|  |  |  |  | 7 vs. 0 | 0.1670 | ns |
|  |  |  |  | 8 vs. 0 | 0.5111 | ns |
| 9 | Kruskal-Wallis | 0.0001 | 0.0142 | 1 vs. 0 | 0.1695 | ns |
|  |  |  |  | 2 vs. 0 | 0.1695 | ns |
|  |  |  |  | 3 vs. 0 | 0.4591 | ns |
|  |  |  |  | 4 vs. 0 | 0.5188 | ns |
|  |  |  |  | 6 vs. 0 | 0.6547 | ns |
| -5 | ANOVA | 0.4676 | 0.8721 | 4 vs. 0 | 0.6417 | ns |
|  |  |  |  | 5 vs. 0 | 0.7316 | ns |
|  |  |  |  | 6 vs. 0 | 0.3395 | ns |
|  |  |  |  | 7 vs. 0 | 0.6417 | ns |
|  |  |  |  | 8 vs. 0 | 0.8755 | ns |
| -6 | ANOVA | 0.2260 | 0.9821 | 1 vs. 0 | 0.5644 | ns |

| **Day** | **Test** | **Normality p** | **Main p** | **Comparison** | **Pairwise p** | **Significance** |
| --- | --- | --- | --- | --- | --- | --- |
|  |  |  |  | 2 vs. 0 | 0.7534 | ns |
|  |  |  |  | 3 vs. 0 | 0.6635 | ns |
|  |  |  |  | 4 vs. 0 | 0.4033 | ns |
|  |  |  |  | 5 vs. 0 | 0.7557 | ns |
|  |  |  |  | 6 vs. 0 | 0.7375 | ns |
|  |  |  |  | 7 vs. 0 | 0.2408 | ns |
|  |  |  |  | 8 vs. 0 | 0.8682 | ns |
| 10 | Kruskal-Wallis | 0.0001 | 0.3383 | 1 vs. 0 | 1.0000 | ns |
|  |  |  |  | 2 vs. 0 | 1.0000 | ns |
|  |  |  |  | 3 vs. 0 | 1.0000 | ns |
|  |  |  |  | 4 vs. 0 | 1.0000 | ns |
|  |  |  |  | 5 vs. 0 | 1.0000 | ns |
|  |  |  |  | 6 vs. 0 | 1.0000 | ns |
|  |  |  |  | 7 vs. 0 | 1.0000 | ns |
|  |  |  |  | 8 vs. 0 | 1.0000 | ns |
| 12 | ANOVA | 0.0187 | 0.8847 | 4 vs. 0 | 0.5463 | ns |
|  |  |  |  | 5 vs. 0 | 0.8090 | ns |
|  |  |  |  | 6 vs. 0 | 0.8746 | ns |
|  |  |  |  | 7 vs. 0 | 0.7865 | ns |
|  |  |  |  | 8 vs. 0 | 0.9377 | ns |
| 14 | Kruskal-Wallis | 0.0001 | 0.9325 | 1 vs. 0 | 1.0000 | ns |
|  |  |  |  | 2 vs. 0 | 1.0000 | ns |
|  |  |  |  | 3 vs. 0 | 1.0000 | ns |
|  |  |  |  | 4 vs. 0 | 1.0000 | ns |
|  |  |  |  | 5 vs. 0 | 1.0000 | ns |
|  |  |  |  | 6 vs. 0 | 1.0000 | ns |

| **Day** | **Test** | **Normality p** | **Main p** | **Comparison** | **Pairwise p** | **Significance** |
| --- | --- | --- | --- | --- | --- | --- |
|  |  |  |  | 7 vs. 0 | 1.0000 | ns |
|  |  |  |  | 8 vs. 0 | 1.0000 | ns |
| 16 | Kruskal-Wallis | 0.0001 | 0.1306 | 4 vs. 0 | 0.7162 | ns |
|  |  |  |  | 5 vs. 0 | 0.7162 | ns |
|  |  |  |  | 6 vs. 0 | 0.7162 | ns |
|  |  |  |  | 7 vs. 0 | 0.7162 | ns |
|  |  |  |  | 8 vs. 0 | 0.7162 | ns |
| 18 | Kruskal-Wallis | 0.0001 | 0.3903 | 4 vs. 0 | 0.7162 | ns |
|  |  |  |  | 5 vs. 0 | 0.7162 | ns |
|  |  |  |  | 6 vs. 0 | 0.7162 | ns |
|  |  |  |  | 7 vs. 0 | 0.7162 | ns |
|  |  |  |  | 8 vs. 0 | 0.7162 | ns |
| 20 | ANOVA | 0.7642 | 0.5647 | 4 vs. 0 | 0.1686 | ns |
|  |  |  |  | 5 vs. 0 | 0.3053 | ns |
|  |  |  |  | 6 vs. 0 | 0.4747 | ns |
|  |  |  |  | 7 vs. 0 | 0.6328 | ns |
|  |  |  |  | 8 vs. 0 | 0.3925 | ns |
| 21 | ANOVA | 0.2754 | 0.4189 | 1 vs. 0 | 0.3162 | ns |
|  |  |  |  | 2 vs. 0 | 0.2254 | ns |
|  |  |  |  | 3 vs. 0 | 0.1091 | ns |
|  |  |  |  | 4 vs. 0 | 0.1081 | ns |
| 28 | ANOVA | 0.0033 | 0.6714 | 1 vs. 0 | 0.6800 | ns |
|  |  |  |  | 2 vs. 0 | 0.7104 | ns |
|  |  |  |  | 3 vs. 0 | 0.3937 | ns |
|  |  |  |  | 4 vs. 0 | 0.8058 | ns |
|  |  |  |  | 5 vs. 0 | 0.7908 | ns |

| **Day** | **Test** | **Normality p** | **Main p** | **Comparison** | **Pairwise p** | **Significance** |
| --- | --- | --- | --- | --- | --- | --- |
|  |  |  |  | 6 vs. 0 | 0.8610 | ns |
|  |  |  |  | 7 vs. 0 | 0.8735 | ns |
|  |  |  |  | 8 vs. 0 | 0.8618 | ns |
| 35 | ANOVA | 0.2364 | 0.6535 | 1 vs. 0 | 0.6884 | ns |
|  |  |  |  | 2 vs. 0 | 0.3157 | ns |
|  |  |  |  | 3 vs. 0 | 0.2054 | ns |
|  |  |  |  | 4 vs. 0 | 0.5863 | ns |
|  |  |  |  | 5 vs. 0 | 0.8747 | ns |
|  |  |  |  | 6 vs. 0 | 0.7550 | ns |
|  |  |  |  | 7 vs. 0 | 0.9832 | ns |
|  |  |  |  | 8 vs. 0 | 0.8361 | ns |
| 44 | ANOVA | 0.5438 | 0.3924 | 4 vs. 0 | 0.4014 | ns |
|  |  |  |  | 5 vs. 0 | 0.9767 | ns |
|  |  |  |  | 6 vs. 0 | 0.7350 | ns |
|  |  |  |  | 7 vs. 0 | 0.9767 | ns |
|  |  |  |  | 8 vs. 0 | 0.5859 | ns |
| 45 | ANOVA | 0.0075 | 0.8959 | 1 vs. 0 | 0.5679 | ns |
|  |  |  |  | 2 vs. 0 | 0.7598 | ns |
|  |  |  |  | 3 vs. 0 | 0.8822 | ns |
|  |  |  |  | 4 vs. 0 | 0.9500 | ns |
| -32 | ANOVA | 0.0292 | 0.5117 | 4 vs. 0 | 0.2993 | ns |
|  |  |  |  | 5 vs. 0 | 0.7979 | ns |
|  |  |  |  | 6 vs. 0 | 0.4203 | ns |
|  |  |  |  | 7 vs. 0 | 0.4372 | ns |
|  |  |  |  | 8 vs. 0 | 0.5909 | ns |
| -33 | ANOVA | 0.2447 | 0.7697 | 4 vs. 0 | 0.9191 | ns |

| **Day** | **Test** | **Normality p** | **Main p** | **Comparison** | **Pairwise p** | **Significance** |
| --- | --- | --- | --- | --- | --- | --- |
|  |  |  |  | 5 vs. 0 | 0.5715 | ns |
|  |  |  |  | 6 vs. 0 | 0.2802 | ns |
|  |  |  |  | 7 vs. 0 | 0.6393 | ns |
|  |  |  |  | 8 vs. 0 | 0.7616 | ns |
| -34 | ANOVA | 0.1417 | 0.8674 | 4 vs. 0 | 0.8613 | ns |
|  |  |  |  | 5 vs. 0 | 0.7443 | ns |
|  |  |  |  | 6 vs. 0 | 0.5080 | ns |
|  |  |  |  | 7 vs. 0 | 0.8207 | ns |
|  |  |  |  | 8 vs. 0 | 0.7821 | ns |

| **Day** | **Test** | **Normality p** | **Main p** | **Comparison** | **Pairwise p** | **Significance** |
| --- | --- | --- | --- | --- | --- | --- |
| 3 | Kruskal-Wallis | 0.0001 | 0.2959 | 1 vs. 0 | 1.0000 | ns |
|  |  |  |  | 2 vs. 0 | 1.0000 | ns |
|  |  |  |  | 3 vs. 0 | 0.7034 | ns |
|  |  |  |  | 4 vs. 0 | 0.5925 | ns |
| 4 | ANOVA | 0.8239 | 0.1265 | 4 vs. 0 | 0.0803 | ns |
|  |  |  |  | 5 vs. 0 | 0.0396 | p<0.05 |
|  |  |  |  | 6 vs. 0 | 0.1582 | ns |
|  |  |  |  | 7 vs. 0 | 0.3949 | ns |
|  |  |  |  | 8 vs. 0 | 0.0131 | p<0.05 |
| 5 | ANOVA | 0.9965 | <.0001 | 1 vs. 0 | 0.0036 | p<0.05 |
|  |  |  |  | 2 vs. 0 | <.0001 | p<0.05 |
|  |  |  |  | 3 vs. 0 | 0.0022 | p<0.05 |
|  |  |  |  | 4 vs. 0 | 0.1009 | ns |
|  |  |  |  | 5 vs. 0 | 0.1767 | ns |
|  |  |  |  | 6 vs. 0 | 0.1115 | ns |
|  |  |  |  | 7 vs. 0 | 0.5358 | ns |
|  |  |  |  | 8 vs. 0 | 0.5476 | ns |
| 6 | ANOVA | 0.4940 | 0.0008 | 1 vs. 0 | 0.0013 | p<0.05 |
|  |  |  |  | 2 vs. 0 | 0.0029 | p<0.05 |
|  |  |  |  | 3 vs. 0 | 0.0058 | p<0.05 |
|  |  |  |  | 4 vs. 0 | 0.0587 | ns |
|  |  |  |  | 5 vs. 0 | 0.8493 | ns |
|  |  |  |  | 6 vs. 0 | 0.7513 | ns |
|  |  |  |  | 7 vs. 0 | 0.8914 | ns |
|  |  |  |  | 8 vs. 0 | 0.7190 | ns |
| 7 | ANOVA | 0.6413 | 0.0033 | 1 vs. 0 | 0.6172 | ns |

| **Day** | **Test** | **Normality p** | **Main p** | **Comparison** | **Pairwise p** | **Significance** |
| --- | --- | --- | --- | --- | --- | --- |
|  |  |  |  | 2 vs. 0 | 0.0349 | p<0.05 |
|  |  |  |  | 3 vs. 0 | 0.1999 | ns |
|  |  |  |  | 4 vs. 0 | 0.1378 | ns |
|  |  |  |  | 5 vs. 0 | 0.7170 | ns |
|  |  |  |  | 6 vs. 0 | 0.5758 | ns |
|  |  |  |  | 7 vs. 0 | 0.3684 | ns |
|  |  |  |  | 8 vs. 0 | 0.0185 | p<0.05 |
| 8 | ANOVA | 0.0196 | 0.0112 | 1 vs. 0 | 0.0172 | p<0.05 |
|  |  |  |  | 2 vs. 0 | 0.0010 | p<0.05 |
|  |  |  |  | 3 vs. 0 | 0.0606 | ns |
|  |  |  |  | 4 vs. 0 | 0.0144 | p<0.05 |
|  |  |  |  | 5 vs. 0 | 0.0989 | ns |
|  |  |  |  | 6 vs. 0 | 0.5048 | ns |
|  |  |  |  | 7 vs. 0 | 0.5405 | ns |
|  |  |  |  | 8 vs. 0 | 0.7270 | ns |
| 9 | ANOVA | 0.0149 | <.0001 | 1 vs. 0 | <.0001 | p<0.05 |
|  |  |  |  | 2 vs. 0 | <.0001 | p<0.05 |
|  |  |  |  | 3 vs. 0 | <.0001 | p<0.05 |
|  |  |  |  | 4 vs. 0 | <.0001 | p<0.05 |
|  |  |  |  | 6 vs. 0 | <.0001 | p<0.05 |
| -5 | ANOVA | 0.8477 | 0.8993 | 4 vs. 0 | 0.6089 | ns |
|  |  |  |  | 5 vs. 0 | 0.8196 | ns |
|  |  |  |  | 6 vs. 0 | 0.7279 | ns |
|  |  |  |  | 7 vs. 0 | 0.8002 | ns |
|  |  |  |  | 8 vs. 0 | 0.6110 | ns |
| -6 | ANOVA | 0.4923 | 0.8026 | 1 vs. 0 | 0.5425 | ns |

| **Day** | **Test** | **Normality p** | **Main p** | **Comparison** | **Pairwise p** | **Significance** |
| --- | --- | --- | --- | --- | --- | --- |
|  |  |  |  | 2 vs. 0 | 0.8759 | ns |
|  |  |  |  | 3 vs. 0 | 0.7839 | ns |
|  |  |  |  | 4 vs. 0 | 0.3608 | ns |
|  |  |  |  | 5 vs. 0 | 0.4388 | ns |
|  |  |  |  | 6 vs. 0 | 0.6448 | ns |
|  |  |  |  | 7 vs. 0 | 0.7985 | ns |
|  |  |  |  | 8 vs. 0 | 0.6775 | ns |
| 10 | ANOVA | 0.0317 | <.0001 | 1 vs. 0 | 0.0068 | p<0.05 |
|  |  |  |  | 2 vs. 0 | 0.0051 | p<0.05 |
|  |  |  |  | 3 vs. 0 | 0.0036 | p<0.05 |
|  |  |  |  | 4 vs. 0 | 0.0059 | p<0.05 |
|  |  |  |  | 5 vs. 0 | 0.1607 | ns |
|  |  |  |  | 6 vs. 0 | 0.2335 | ns |
|  |  |  |  | 7 vs. 0 | 0.6524 | ns |
|  |  |  |  | 8 vs. 0 | 0.0126 | p<0.05 |
| 12 | ANOVA | 0.6889 | 0.6313 | 4 vs. 0 | 0.8606 | ns |
|  |  |  |  | 5 vs. 0 | 0.6726 | ns |
|  |  |  |  | 6 vs. 0 | 0.8055 | ns |
|  |  |  |  | 7 vs. 0 | 0.3125 | ns |
|  |  |  |  | 8 vs. 0 | 0.3634 | ns |
| 14 | ANOVA | 0.2603 | 0.2963 | 1 vs. 0 | 0.8433 | ns |
|  |  |  |  | 2 vs. 0 | 0.1839 | ns |
|  |  |  |  | 3 vs. 0 | 0.2050 | ns |
|  |  |  |  | 4 vs. 0 | 0.3249 | ns |
|  |  |  |  | 5 vs. 0 | 0.8734 | ns |
|  |  |  |  | 6 vs. 0 | 0.4235 | ns |

| **Day** | **Test** | **Normality p** | **Main p** | **Comparison** | **Pairwise p** | **Significance** |
| --- | --- | --- | --- | --- | --- | --- |
|  |  |  |  | 7 vs. 0 | 0.8075 | ns |
|  |  |  |  | 8 vs. 0 | 0.7325 | ns |
| 16 | ANOVA | 0.1373 | 0.8924 | 4 vs. 0 | 0.5383 | ns |
|  |  |  |  | 5 vs. 0 | 0.8748 | ns |
|  |  |  |  | 6 vs. 0 | 0.7831 | ns |
|  |  |  |  | 7 vs. 0 | 0.9475 | ns |
|  |  |  |  | 8 vs. 0 | 0.8834 | ns |
| 18 | ANOVA | 0.3738 | 0.3730 | 4 vs. 0 | 0.2942 | ns |
|  |  |  |  | 5 vs. 0 | 0.8748 | ns |
|  |  |  |  | 6 vs. 0 | 0.8175 | ns |
|  |  |  |  | 7 vs. 0 | 0.7657 | ns |
|  |  |  |  | 8 vs. 0 | 0.6292 | ns |
| 20 | ANOVA | 0.2998 | 0.2310 | 4 vs. 0 | 0.2251 | ns |
|  |  |  |  | 5 vs. 0 | 0.6248 | ns |
|  |  |  |  | 6 vs. 0 | 0.6613 | ns |
|  |  |  |  | 7 vs. 0 | 0.5148 | ns |
|  |  |  |  | 8 vs. 0 | 0.5876 | ns |
| 21 | ANOVA | 0.5474 | 0.3806 | 1 vs. 0 | 0.8893 | ns |
|  |  |  |  | 2 vs. 0 | 0.5434 | ns |
|  |  |  |  | 3 vs. 0 | 0.3054 | ns |
|  |  |  |  | 4 vs. 0 | 0.8384 | ns |
| 28 | ANOVA | 0.0255 | 0.2460 | 1 vs. 0 | 0.1117 | ns |
|  |  |  |  | 2 vs. 0 | 0.0916 | ns |
|  |  |  |  | 3 vs. 0 | 0.0109 | p<0.05 |
|  |  |  |  | 4 vs. 0 | 0.0493 | p<0.05 |
|  |  |  |  | 5 vs. 0 | 0.1390 | ns |

| **Day** | **Test** | **Normality p** | **Main p** | **Comparison** | **Pairwise p** | **Significance** |
| --- | --- | --- | --- | --- | --- | --- |
|  |  |  |  | 6 vs. 0 | 0.0417 | p<0.05 |
|  |  |  |  | 7 vs. 0 | 0.0417 | p<0.05 |
|  |  |  |  | 8 vs. 0 | 0.4751 | ns |
| 35 | ANOVA | 0.0018 | 0.4577 | 1 vs. 0 | 0.2076 | ns |
|  |  |  |  | 2 vs. 0 | 0.7733 | ns |
|  |  |  |  | 3 vs. 0 | 0.8431 | ns |
|  |  |  |  | 4 vs. 0 | 0.5487 | ns |
|  |  |  |  | 5 vs. 0 | 0.9461 | ns |
|  |  |  |  | 6 vs. 0 | 0.9544 | ns |
|  |  |  |  | 7 vs. 0 | 0.4406 | ns |
|  |  |  |  | 8 vs. 0 | 0.2325 | ns |
| 44 | ANOVA | 0.5457 | 0.0252 | 4 vs. 0 | 0.0015 | p<0.05 |
|  |  |  |  | 5 vs. 0 | 0.0054 | p<0.05 |
|  |  |  |  | 6 vs. 0 | 0.0164 | p<0.05 |
|  |  |  |  | 7 vs. 0 | 0.0103 | p<0.05 |
|  |  |  |  | 8 vs. 0 | 0.0643 | ns |
| 45 | Kruskal-Wallis | 0.0007 | 0.9793 | 1 vs. 0 | 1.0000 | ns |
|  |  |  |  | 2 vs. 0 | 1.0000 | ns |
|  |  |  |  | 3 vs. 0 | 1.0000 | ns |
|  |  |  |  | 4 vs. 0 | 1.0000 | ns |
| -32 | ANOVA | 0.1425 | 0.8017 | 4 vs. 0 | 0.6290 | ns |
|  |  |  |  | 5 vs. 0 | 0.2916 | ns |
|  |  |  |  | 6 vs. 0 | 0.8733 | ns |
|  |  |  |  | 7 vs. 0 | 0.6916 | ns |
|  |  |  |  | 8 vs. 0 | 0.7401 | ns |
| -33 | ANOVA | 0.7622 | 0.4196 | 4 vs. 0 | 0.5414 | ns |

| **Day** | **Test** | **Normality p** | **Main p** | **Comparison** | **Pairwise p** | **Significance** |
| --- | --- | --- | --- | --- | --- | --- |
|  |  |  |  | 5 vs. 0 | 0.7239 | ns |
|  |  |  |  | 6 vs. 0 | 0.3378 | ns |
|  |  |  |  | 7 vs. 0 | 0.7814 | ns |
|  |  |  |  | 8 vs. 0 | 0.6127 | ns |
| -34 | ANOVA | 0.6412 | 0.0406 | 4 vs. 0 | 0.5168 | ns |
|  |  |  |  | 5 vs. 0 | 0.3272 | ns |
|  |  |  |  | 6 vs. 0 | 0.6655 | ns |
|  |  |  |  | 7 vs. 0 | 0.0322 | p<0.05 |
|  |  |  |  | 8 vs. 0 | 0.8003 | ns |

| **Day** | **Test** | **Normality p** | **Main p** | **Comparison** | **Pairwise p** | **Significance** |
| --- | --- | --- | --- | --- | --- | --- |
| 3 | Kruskal-Wallis | 0.0003 | 0.7280 | 1 vs. 0 | 1.0000 | ns |
|  |  |  |  | 2 vs. 0 | 1.0000 | ns |
|  |  |  |  | 3 vs. 0 | 1.0000 | ns |
|  |  |  |  | 4 vs. 0 | 0.3577 | ns |
| 4 | ANOVA | 0.7702 | 0.1717 | 4 vs. 0 | 0.0607 | ns |
|  |  |  |  | 5 vs. 0 | 0.0314 | p<0.05 |
|  |  |  |  | 6 vs. 0 | 0.1229 | ns |
|  |  |  |  | 7 vs. 0 | 0.1859 | ns |
|  |  |  |  | 8 vs. 0 | 0.0139 | p<0.05 |
| 5 | ANOVA | 0.4286 | 0.0033 | 1 vs. 0 | 0.1703 | ns |
|  |  |  |  | 2 vs. 0 | 0.0386 | p<0.05 |
|  |  |  |  | 3 vs. 0 | 0.0274 | p<0.05 |
|  |  |  |  | 4 vs. 0 | 0.4713 | ns |
|  |  |  |  | 5 vs. 0 | 0.3214 | ns |
|  |  |  |  | 6 vs. 0 | 0.1218 | ns |
|  |  |  |  | 7 vs. 0 | 0.5899 | ns |
|  |  |  |  | 8 vs. 0 | 0.6477 | ns |
| 6 | ANOVA | 0.7125 | 0.3178 | 1 vs. 0 | 0.0772 | ns |
|  |  |  |  | 2 vs. 0 | 0.2738 | ns |
|  |  |  |  | 3 vs. 0 | 0.1373 | ns |
|  |  |  |  | 4 vs. 0 | 0.3109 | ns |
|  |  |  |  | 5 vs. 0 | 0.8357 | ns |
|  |  |  |  | 6 vs. 0 | 0.9458 | ns |
|  |  |  |  | 7 vs. 0 | 0.9810 | ns |
|  |  |  |  | 8 vs. 0 | 0.6931 | ns |
| 7 | ANOVA | 0.7493 | 0.0658 | 1 vs. 0 | 0.8627 | ns |

| **Day** | **Test** | **Normality p** | **Main p** | **Comparison** | **Pairwise p** | **Significance** |
| --- | --- | --- | --- | --- | --- | --- |
|  |  |  |  | 2 vs. 0 | 0.8951 | ns |
|  |  |  |  | 3 vs. 0 | 0.5282 | ns |
|  |  |  |  | 4 vs. 0 | 0.5169 | ns |
|  |  |  |  | 5 vs. 0 | 0.7652 | ns |
|  |  |  |  | 6 vs. 0 | 0.5904 | ns |
|  |  |  |  | 7 vs. 0 | 0.3288 | ns |
|  |  |  |  | 8 vs. 0 | 0.0092 | p<0.05 |
| 8 | ANOVA | 0.0820 | 0.0232 | 1 vs. 0 | 0.0263 | p<0.05 |
|  |  |  |  | 2 vs. 0 | 0.0130 | p<0.05 |
|  |  |  |  | 3 vs. 0 | 0.0511 | ns |
|  |  |  |  | 4 vs. 0 | 0.0358 | p<0.05 |
|  |  |  |  | 5 vs. 0 | 0.1642 | ns |
|  |  |  |  | 6 vs. 0 | 0.8159 | ns |
|  |  |  |  | 7 vs. 0 | 0.9176 | ns |
|  |  |  |  | 8 vs. 0 | 0.7395 | ns |
| 9 | ANOVA | 0.0040 | <.0001 | 1 vs. 0 | <.0001 | p<0.05 |
|  |  |  |  | 2 vs. 0 | <.0001 | p<0.05 |
|  |  |  |  | 3 vs. 0 | <.0001 | p<0.05 |
|  |  |  |  | 4 vs. 0 | <.0001 | p<0.05 |
|  |  |  |  | 6 vs. 0 | 0.0082 | p<0.05 |
| -5 | ANOVA | 0.4133 | 0.6029 | 4 vs. 0 | 0.4155 | ns |
|  |  |  |  | 5 vs. 0 | 0.9774 | ns |
|  |  |  |  | 6 vs. 0 | 0.7110 | ns |
|  |  |  |  | 7 vs. 0 | 0.3565 | ns |
|  |  |  |  | 8 vs. 0 | 0.8087 | ns |
| -6 | ANOVA | 0.4647 | 0.9781 | 1 vs. 0 | 0.5444 | ns |

| **Day** | **Test** | **Normality p** | **Main p** | **Comparison** | **Pairwise p** | **Significance** |
| --- | --- | --- | --- | --- | --- | --- |
|  |  |  |  | 2 vs. 0 | 0.8506 | ns |
|  |  |  |  | 3 vs. 0 | 0.5622 | ns |
|  |  |  |  | 4 vs. 0 | 0.8737 | ns |
|  |  |  |  | 5 vs. 0 | 0.9198 | ns |
|  |  |  |  | 6 vs. 0 | 0.7253 | ns |
|  |  |  |  | 7 vs. 0 | 0.7602 | ns |
|  |  |  |  | 8 vs. 0 | 0.7284 | ns |
| 10 | ANOVA | 0.0204 | 0.0283 | 1 vs. 0 | 0.0351 | p<0.05 |
|  |  |  |  | 2 vs. 0 | 0.0483 | p<0.05 |
|  |  |  |  | 3 vs. 0 | 0.2506 | ns |
|  |  |  |  | 4 vs. 0 | 0.1740 | ns |
|  |  |  |  | 5 vs. 0 | 0.3970 | ns |
|  |  |  |  | 6 vs. 0 | 0.8374 | ns |
|  |  |  |  | 7 vs. 0 | 0.5807 | ns |
|  |  |  |  | 8 vs. 0 | 0.2779 | ns |
| 12 | ANOVA | 0.9288 | 0.4945 | 4 vs. 0 | 0.3670 | ns |
|  |  |  |  | 5 vs. 0 | 0.2511 | ns |
|  |  |  |  | 6 vs. 0 | 0.1633 | ns |
|  |  |  |  | 7 vs. 0 | 0.0667 | ns |
|  |  |  |  | 8 vs. 0 | 0.3872 | ns |
| 14 | ANOVA | 0.9219 | 0.2586 | 1 vs. 0 | 0.6292 | ns |
|  |  |  |  | 2 vs. 0 | 0.7447 | ns |
|  |  |  |  | 3 vs. 0 | 0.9138 | ns |
|  |  |  |  | 4 vs. 0 | 0.6204 | ns |
|  |  |  |  | 5 vs. 0 | 0.3823 | ns |
|  |  |  |  | 6 vs. 0 | 0.4417 | ns |

| **Day** | **Test** | **Normality p** | **Main p** | **Comparison** | **Pairwise p** | **Significance** |
| --- | --- | --- | --- | --- | --- | --- |
|  |  |  |  | 7 vs. 0 | 0.1269 | ns |
|  |  |  |  | 8 vs. 0 | 0.7671 | ns |
| 16 | ANOVA | 0.0861 | 0.8623 | 4 vs. 0 | 0.9053 | ns |
|  |  |  |  | 5 vs. 0 | 0.9010 | ns |
|  |  |  |  | 6 vs. 0 | 0.8141 | ns |
|  |  |  |  | 7 vs. 0 | 0.8218 | ns |
|  |  |  |  | 8 vs. 0 | 0.6125 | ns |
| 18 | ANOVA | 0.9185 | 0.8063 | 4 vs. 0 | 0.7206 | ns |
|  |  |  |  | 5 vs. 0 | 0.9337 | ns |
|  |  |  |  | 6 vs. 0 | 0.7775 | ns |
|  |  |  |  | 7 vs. 0 | 0.7678 | ns |
|  |  |  |  | 8 vs. 0 | 0.7179 | ns |
| 20 | ANOVA | 0.7118 | 0.7254 | 4 vs. 0 | 0.7156 | ns |
|  |  |  |  | 5 vs. 0 | 0.9352 | ns |
|  |  |  |  | 6 vs. 0 | 0.5089 | ns |
|  |  |  |  | 7 vs. 0 | 0.9113 | ns |
|  |  |  |  | 8 vs. 0 | 0.9808 | ns |
| 21 | ANOVA | 0.1966 | 0.7597 | 1 vs. 0 | 0.3713 | ns |
|  |  |  |  | 2 vs. 0 | 0.3986 | ns |
|  |  |  |  | 3 vs. 0 | 0.4640 | ns |
|  |  |  |  | 4 vs. 0 | 0.2495 | ns |
| 28 | ANOVA | 0.2486 | 0.2209 | 1 vs. 0 | 0.1022 | ns |
|  |  |  |  | 2 vs. 0 | 0.2421 | ns |
|  |  |  |  | 3 vs. 0 | 0.0632 | ns |
|  |  |  |  | 4 vs. 0 | 0.1248 | ns |
|  |  |  |  | 5 vs. 0 | 0.6135 | ns |

| **Day** | **Test** | **Normality p** | **Main p** | **Comparison** | **Pairwise p** | **Significance** |
| --- | --- | --- | --- | --- | --- | --- |
|  |  |  |  | 6 vs. 0 | 0.8427 | ns |
|  |  |  |  | 7 vs. 0 | 0.5305 | ns |
|  |  |  |  | 8 vs. 0 | 0.3783 | ns |
| 35 | ANOVA | 0.2992 | 0.7058 | 1 vs. 0 | 0.7141 | ns |
|  |  |  |  | 2 vs. 0 | 0.9789 | ns |
|  |  |  |  | 3 vs. 0 | 0.6542 | ns |
|  |  |  |  | 4 vs. 0 | 0.9022 | ns |
|  |  |  |  | 5 vs. 0 | 0.7187 | ns |
|  |  |  |  | 6 vs. 0 | 0.5207 | ns |
|  |  |  |  | 7 vs. 0 | 0.3515 | ns |
|  |  |  |  | 8 vs. 0 | 0.9193 | ns |
| 44 | ANOVA | 0.7052 | 0.2660 | 4 vs. 0 | 0.1438 | ns |
|  |  |  |  | 5 vs. 0 | 0.3151 | ns |
|  |  |  |  | 6 vs. 0 | 0.9263 | ns |
|  |  |  |  | 7 vs. 0 | 0.5643 | ns |
|  |  |  |  | 8 vs. 0 | 0.2848 | ns |
| 45 | ANOVA | 0.0067 | 0.9094 | 1 vs. 0 | 0.3796 | ns |
|  |  |  |  | 2 vs. 0 | 0.3756 | ns |
|  |  |  |  | 3 vs. 0 | 0.4802 | ns |
|  |  |  |  | 4 vs. 0 | 0.4582 | ns |
| -32 | ANOVA | 0.0386 | 0.7751 | 4 vs. 0 | 0.5794 | ns |
|  |  |  |  | 5 vs. 0 | 0.4406 | ns |
|  |  |  |  | 6 vs. 0 | 0.9269 | ns |
|  |  |  |  | 7 vs. 0 | 0.9109 | ns |
|  |  |  |  | 8 vs. 0 | 0.6500 | ns |
| -33 | ANOVA | 0.7643 | 0.3472 | 4 vs. 0 | 0.5417 | ns |

| **Day** | **Test** | **Normality p** | **Main p** | **Comparison** | **Pairwise p** | **Significance** |
| --- | --- | --- | --- | --- | --- | --- |
|  |  |  |  | 5 vs. 0 | 0.6457 | ns |
|  |  |  |  | 6 vs. 0 | 0.1240 | ns |
|  |  |  |  | 7 vs. 0 | 0.1776 | ns |
|  |  |  |  | 8 vs. 0 | 0.8226 | ns |
| -34 | ANOVA | 0.4992 | 0.8609 | 4 vs. 0 | 0.7948 | ns |
|  |  |  |  | 5 vs. 0 | 0.6165 | ns |
|  |  |  |  | 6 vs. 0 | 0.6190 | ns |
|  |  |  |  | 7 vs. 0 | 0.3381 | ns |
|  |  |  |  | 8 vs. 0 | 0.8536 | ns |

| **Day** | **Test** | **Normality p** | **Main p** | **Comparison** | **Pairwise p** | **Significance** |
| --- | --- | --- | --- | --- | --- | --- |
| 3 | Kruskal-Wallis | 0.0001 | 0.7698 | 1 vs. 0 | 1.0000 | ns |
|  |  |  |  | 2 vs. 0 | 1.0000 | ns |
|  |  |  |  | 3 vs. 0 | 1.0000 | ns |
|  |  |  |  | 4 vs. 0 | 1.0000 | ns |
| 4 | Kruskal-Wallis | 0.0001 | 0.1387 | 4 vs. 0 | 0.1732 | ns |
|  |  |  |  | 5 vs. 0 | 0.2642 | ns |
|  |  |  |  | 6 vs. 0 | 0.0951 | ns |
|  |  |  |  | 7 vs. 0 | 0.3290 | ns |
|  |  |  |  | 8 vs. 0 | 0.3290 | ns |
| 5 | Kruskal-Wallis | 0.0001 | <.0001 | 1 vs. 0 | 0.0340 | p<0.05 |
|  |  |  |  | 2 vs. 0 | 0.0088 | p<0.05 |
|  |  |  |  | 3 vs. 0 | 0.0026 | p<0.05 |
|  |  |  |  | 4 vs. 0 | 0.6511 | ns |
|  |  |  |  | 5 vs. 0 | 0.6067 | ns |
|  |  |  |  | 6 vs. 0 | 0.6067 | ns |
|  |  |  |  | 7 vs. 0 | 0.6982 | ns |
|  |  |  |  | 8 vs. 0 | 0.6511 | ns |
| 6 | Kruskal-Wallis | 0.0001 | <.0001 | 1 vs. 0 | 0.0958 | ns |
|  |  |  |  | 2 vs. 0 | 0.0303 | p<0.05 |
|  |  |  |  | 3 vs. 0 | 0.1226 | ns |
|  |  |  |  | 4 vs. 0 | 1.0000 | ns |
|  |  |  |  | 5 vs. 0 | 1.0000 | ns |
|  |  |  |  | 6 vs. 0 | 1.0000 | ns |
|  |  |  |  | 7 vs. 0 | 1.0000 | ns |
|  |  |  |  | 8 vs. 0 | 1.0000 | ns |
| 7 | Kruskal-Wallis | 0.0001 | <.0001 | 1 vs. 0 | 0.0279 | p<0.05 |

| **Day** | **Test** | **Normality p** | **Main p** | **Comparison** | **Pairwise p** | **Significance** |
| --- | --- | --- | --- | --- | --- | --- |
|  |  |  |  | 2 vs. 0 | 0.0075 | p<0.05 |
|  |  |  |  | 3 vs. 0 | 0.0055 | p<0.05 |
|  |  |  |  | 4 vs. 0 | 0.0055 | p<0.05 |
|  |  |  |  | 5 vs. 0 | 0.0563 | ns |
|  |  |  |  | 6 vs. 0 | 0.9035 | ns |
|  |  |  |  | 7 vs. 0 | 0.9035 | ns |
|  |  |  |  | 8 vs. 0 | 1.0000 | ns |
| 8 | Kruskal-Wallis | 0.0001 | <.0001 | 1 vs. 0 | 0.0284 | p<0.05 |
|  |  |  |  | 2 vs. 0 | 0.0088 | p<0.05 |
|  |  |  |  | 3 vs. 0 | 0.0062 | p<0.05 |
|  |  |  |  | 4 vs. 0 | 0.0157 | p<0.05 |
|  |  |  |  | 5 vs. 0 | 0.0805 | ns |
|  |  |  |  | 6 vs. 0 | 0.1166 | ns |
|  |  |  |  | 7 vs. 0 | 0.3112 | ns |
|  |  |  |  | 8 vs. 0 | 0.5582 | ns |
| 9 | Kruskal-Wallis | 0.0001 | 0.0003 | 1 vs. 0 | 0.0277 | p<0.05 |
|  |  |  |  | 2 vs. 0 | 0.0197 | p<0.05 |
|  |  |  |  | 3 vs. 0 | 0.0248 | p<0.05 |
|  |  |  |  | 4 vs. 0 | 0.0248 | p<0.05 |
|  |  |  |  | 6 vs. 0 | 0.1573 | ns |
| 10 | Kruskal-Wallis | 0.0001 | <.0001 | 1 vs. 0 | 0.0526 | ns |
|  |  |  |  | 2 vs. 0 | 0.0169 | p<0.05 |
|  |  |  |  | 3 vs. 0 | 0.0067 | p<0.05 |
|  |  |  |  | 4 vs. 0 | 0.0067 | p<0.05 |
|  |  |  |  | 5 vs. 0 | 0.1131 | ns |
|  |  |  |  | 6 vs. 0 | 0.1131 | ns |

| **Day** | **Test** | **Normality p** | **Main p** | **Comparison** | **Pairwise p** | **Significance** |
| --- | --- | --- | --- | --- | --- | --- |
|  |  |  |  | 7 vs. 0 | 0.1131 | ns |
|  |  |  |  | 8 vs. 0 | 1.0000 | ns |
| 12 | Kruskal-Wallis | 0.0001 | 0.0947 | 4 vs. 0 | 0.2224 | ns |
|  |  |  |  | 5 vs. 0 | 0.3298 | ns |
|  |  |  |  | 6 vs. 0 | 0.2224 | ns |
|  |  |  |  | 7 vs. 0 | 0.2224 | ns |
|  |  |  |  | 8 vs. 0 | 0.3298 | ns |
| 14 | Kruskal-Wallis | 0.0001 | 0.0131 | 1 vs. 0 | 0.1423 | ns |
|  |  |  |  | 2 vs. 0 | 0.0618 | ns |
|  |  |  |  | 3 vs. 0 | 0.0230 | p<0.05 |
|  |  |  |  | 4 vs. 0 | 0.0640 | ns |
|  |  |  |  | 5 vs. 0 | 0.1423 | ns |
|  |  |  |  | 6 vs. 0 | 0.1423 | ns |
|  |  |  |  | 7 vs. 0 | 0.1423 | ns |
|  |  |  |  | 8 vs. 0 | 0.1423 | ns |
| 16 | ANOVA | 0.0018 | <.0001 | 4 vs. 0 | <.0001 | p<0.05 |
|  |  |  |  | 5 vs. 0 | <.0001 | p<0.05 |
|  |  |  |  | 6 vs. 0 | <.0001 | p<0.05 |
|  |  |  |  | 7 vs. 0 | <.0001 | p<0.05 |
|  |  |  |  | 8 vs. 0 | <.0001 | p<0.05 |
| 18 | Kruskal-Wallis | 0.0005 | 0.0588 | 4 vs. 0 | 0.3917 | ns |
|  |  |  |  | 5 vs. 0 | 0.3917 | ns |
|  |  |  |  | 6 vs. 0 | 0.2275 | ns |
|  |  |  |  | 7 vs. 0 | 0.3917 | ns |
|  |  |  |  | 8 vs. 0 | 0.3917 | ns |
| 20 | ANOVA | 0.0013 | 0.0732 | 4 vs. 0 | 0.0254 | p<0.05 |

| **Day** | **Test** | **Normality p** | **Main p** | **Comparison** | **Pairwise p** | **Significance** |
| --- | --- | --- | --- | --- | --- | --- |
|  |  |  |  | 5 vs. 0 | 0.0060 | p<0.05 |
|  |  |  |  | 6 vs. 0 | 0.0044 | p<0.05 |
|  |  |  |  | 7 vs. 0 | 0.0078 | p<0.05 |
|  |  |  |  | 8 vs. 0 | 0.0308 | p<0.05 |
| 21 | Kruskal-Wallis | 0.0001 | <.0001 | 1 vs. 0 | 0.0455 | p<0.05 |
|  |  |  |  | 2 vs. 0 | 0.0286 | p<0.05 |
|  |  |  |  | 3 vs. 0 | 0.0187 | p<0.05 |
|  |  |  |  | 4 vs. 0 | 0.0187 | p<0.05 |
| 28 | Kruskal-Wallis | 0.0001 | 0.0944 | 1 vs. 0 | 0.7865 | ns |
|  |  |  |  | 2 vs. 0 | 0.4996 | ns |
|  |  |  |  | 3 vs. 0 | 0.3185 | ns |
|  |  |  |  | 4 vs. 0 | 0.1144 | ns |
|  |  |  |  | 5 vs. 0 | 1.0000 | ns |
|  |  |  |  | 6 vs. 0 | 0.7865 | ns |
|  |  |  |  | 7 vs. 0 | 1.0000 | ns |
|  |  |  |  | 8 vs. 0 | 0.7865 | ns |
| 35 | Kruskal-Wallis | 0.0001 | 0.3172 | 1 vs. 0 | 1.0000 | ns |
|  |  |  |  | 2 vs. 0 | 1.0000 | ns |
|  |  |  |  | 3 vs. 0 | 1.0000 | ns |
|  |  |  |  | 4 vs. 0 | 1.0000 | ns |
|  |  |  |  | 5 vs. 0 | 1.0000 | ns |
|  |  |  |  | 6 vs. 0 | 1.0000 | ns |
|  |  |  |  | 7 vs. 0 | 1.0000 | ns |
|  |  |  |  | 8 vs. 0 | 1.0000 | ns |
| 44 | Kruskal-Wallis | 0.0001 | 0.7158 | 4 vs. 0 | 1.0000 | ns |
|  |  |  |  | 5 vs. 0 | 1.0000 | ns |

| **Day** | **Test** | **Normality p** | **Main p** | **Comparison** | **Pairwise p** | **Significance** |
| --- | --- | --- | --- | --- | --- | --- |
|  |  |  |  | 6 vs. 0 | 1.0000 | ns |
|  |  |  |  | 7 vs. 0 | 1.0000 | ns |
|  |  |  |  | 8 vs. 0 | 1.0000 | ns |
| 45 | Kruskal-Wallis | 0.0001 | 0.6671 | 1 vs. 0 | 1.0000 | ns |
|  |  |  |  | 2 vs. 0 | 1.0000 | ns |
|  |  |  |  | 3 vs. 0 | 1.0000 | ns |
|  |  |  |  | 4 vs. 0 | 1.0000 | ns |

| **Day** | **Test** | **Normality p** | **Main p** | **Comparison** | **Pairwise p** | **Significance** |
| --- | --- | --- | --- | --- | --- | --- |
| 1 | Kruskal-Wallis | 0.0001 | 0.3248 | 1 vs. 0 | 1.0000 | ns |
|  |  |  |  | 2 vs. 0 | 1.0000 | ns |
|  |  |  |  | 3 vs. 0 | 0.3351 | ns |
|  |  |  |  | 4 vs. 0 | 1.0000 | ns |
|  |  |  |  | 5 vs. 0 | 1.0000 | ns |
|  |  |  |  | 6 vs. 0 | 1.0000 | ns |
|  |  |  |  | 7 vs. 0 | 1.0000 | ns |
|  |  |  |  | 8 vs. 0 | 1.0000 | ns |
| 2 | ANOVA | 0.0143 | 0.8394 | 1 vs. 0 | 0.9404 | ns |
|  |  |  |  | 2 vs. 0 | 0.8877 | ns |
|  |  |  |  | 3 vs. 0 | 0.3163 | ns |
|  |  |  |  | 4 vs. 0 | 0.6642 | ns |
|  |  |  |  | 5 vs. 0 | 0.2071 | ns |
|  |  |  |  | 6 vs. 0 | 0.5537 | ns |
|  |  |  |  | 7 vs. 0 | 0.6721 | ns |
|  |  |  |  | 8 vs. 0 | 0.8877 | ns |
| 3 | Kruskal-Wallis | 0.0001 | 0.0090 | 1 vs. 0 | 1.0000 | ns |
|  |  |  |  | 2 vs. 0 | 1.0000 | ns |
|  |  |  |  | 3 vs. 0 | 1.0000 | ns |
|  |  |  |  | 4 vs. 0 | 0.5671 | ns |
|  |  |  |  | 5 vs. 0 | 0.2956 | ns |
|  |  |  |  | 6 vs. 0 | 1.0000 | ns |
|  |  |  |  | 7 vs. 0 | 1.0000 | ns |
|  |  |  |  | 8 vs. 0 | 0.3094 | ns |
| 4 | ANOVA | 0.3168 | <.0001 | 1 vs. 0 | 0.0046 | p<0.05 |
|  |  |  |  | 2 vs. 0 | 0.0367 | p<0.05 |

| **Day** | **Test** | **Normality p** | **Main p** | **Comparison** | **Pairwise p** | **Significance** |
| --- | --- | --- | --- | --- | --- | --- |
|  |  |  |  | 3 vs. 0 | 0.0032 | p<0.05 |
|  |  |  |  | 4 vs. 0 | 0.3285 | ns |
|  |  |  |  | 5 vs. 0 | 0.1113 | ns |
|  |  |  |  | 6 vs. 0 | 0.0089 | p<0.05 |
|  |  |  |  | 7 vs. 0 | 0.3217 | ns |
|  |  |  |  | 8 vs. 0 | 0.0001 | p<0.05 |
| 5 | ANOVA | 0.4158 | <.0001 | 1 vs. 0 | <.0001 | p<0.05 |
|  |  |  |  | 2 vs. 0 | <.0001 | p<0.05 |
|  |  |  |  | 3 vs. 0 | <.0001 | p<0.05 |
|  |  |  |  | 4 vs. 0 | <.0001 | p<0.05 |
|  |  |  |  | 5 vs. 0 | 0.2953 | ns |
|  |  |  |  | 6 vs. 0 | 0.2779 | ns |
|  |  |  |  | 7 vs. 0 | 0.0468 | p<0.05 |
|  |  |  |  | 8 vs. 0 | 0.8762 | ns |
| 6 | ANOVA | 0.2376 | <.0001 | 1 vs. 0 | <.0001 | p<0.05 |
|  |  |  |  | 2 vs. 0 | <.0001 | p<0.05 |
|  |  |  |  | 3 vs. 0 | <.0001 | p<0.05 |
|  |  |  |  | 4 vs. 0 | <.0001 | p<0.05 |
|  |  |  |  | 5 vs. 0 | 0.0698 | ns |
|  |  |  |  | 6 vs. 0 | 0.2360 | ns |
|  |  |  |  | 7 vs. 0 | 0.1333 | ns |
|  |  |  |  | 8 vs. 0 | 0.6096 | ns |
| 7 | ANOVA | 0.0699 | <.0001 | 1 vs. 0 | <.0001 | p<0.05 |
|  |  |  |  | 2 vs. 0 | <.0001 | p<0.05 |
|  |  |  |  | 3 vs. 0 | <.0001 | p<0.05 |
|  |  |  |  | 4 vs. 0 | <.0001 | p<0.05 |

| **Day** | **Test** | **Normality p** | **Main p** | **Comparison** | **Pairwise p** | **Significance** |
| --- | --- | --- | --- | --- | --- | --- |
|  |  |  |  | 5 vs. 0 | 0.1022 | ns |
|  |  |  |  | 6 vs. 0 | 0.3316 | ns |
|  |  |  |  | 7 vs. 0 | 0.1748 | ns |
|  |  |  |  | 8 vs. 0 | 0.9216 | ns |
| 8 | ANOVA | 0.0630 | <.0001 | 1 vs. 0 | <.0001 | p<0.05 |
|  |  |  |  | 2 vs. 0 | <.0001 | p<0.05 |
|  |  |  |  | 3 vs. 0 | <.0001 | p<0.05 |
|  |  |  |  | 4 vs. 0 | <.0001 | p<0.05 |
|  |  |  |  | 5 vs. 0 | 0.3744 | ns |
|  |  |  |  | 6 vs. 0 | 0.6793 | ns |
|  |  |  |  | 7 vs. 0 | 0.0024 | p<0.05 |
|  |  |  |  | 8 vs. 0 | 0.2641 | ns |
| 9 | Kruskal-Wallis | 0.0001 | <.0001 | 1 vs. 0 | 0.0372 | p<0.05 |
|  |  |  |  | 2 vs. 0 | 0.0160 | p<0.05 |
|  |  |  |  | 3 vs. 0 | 0.0093 | p<0.05 |
|  |  |  |  | 4 vs. 0 | 0.0122 | p<0.05 |
|  |  |  |  | 5 vs. 0 | 0.0635 | ns |
|  |  |  |  | 6 vs. 0 | 0.3959 | ns |
|  |  |  |  | 7 vs. 0 | 0.0385 | p<0.05 |
|  |  |  |  | 8 vs. 0 | 0.5699 | ns |
| 10 | ANOVA | 0.0093 | <.0001 | 1 vs. 0 | <.0001 | p<0.05 |
|  |  |  |  | 2 vs. 0 | <.0001 | p<0.05 |
|  |  |  |  | 3 vs. 0 | <.0001 | p<0.05 |
|  |  |  |  | 4 vs. 0 | <.0001 | p<0.05 |
|  |  |  |  | 5 vs. 0 | 0.0133 | p<0.05 |
|  |  |  |  | 6 vs. 0 | 0.0454 | p<0.05 |

| **Day** | **Test** | **Normality p** | **Main p** | **Comparison** | **Pairwise p** | **Significance** |
| --- | --- | --- | --- | --- | --- | --- |
|  |  |  |  | 7 vs. 0 | 0.0113 | p<0.05 |
|  |  |  |  | 8 vs. 0 | 0.2401 | ns |
| 11 | ANOVA | 0.0076 | <.0001 | 1 vs. 0 | <.0001 | p<0.05 |
|  |  |  |  | 2 vs. 0 | <.0001 | p<0.05 |
|  |  |  |  | 3 vs. 0 | <.0001 | p<0.05 |
|  |  |  |  | 4 vs. 0 | <.0001 | p<0.05 |
|  |  |  |  | 5 vs. 0 | 0.0114 | p<0.05 |
|  |  |  |  | 6 vs. 0 | 0.0082 | p<0.05 |
|  |  |  |  | 7 vs. 0 | 0.0918 | ns |
|  |  |  |  | 8 vs. 0 | 0.1919 | ns |
| 12 | ANOVA | 0.0759 | <.0001 | 1 vs. 0 | <.0001 | p<0.05 |
|  |  |  |  | 2 vs. 0 | <.0001 | p<0.05 |
|  |  |  |  | 3 vs. 0 | <.0001 | p<0.05 |
|  |  |  |  | 4 vs. 0 | <.0001 | p<0.05 |
|  |  |  |  | 5 vs. 0 | 0.0006 | p<0.05 |
|  |  |  |  | 6 vs. 0 | 0.0007 | p<0.05 |
|  |  |  |  | 7 vs. 0 | 0.0044 | p<0.05 |
|  |  |  |  | 8 vs. 0 | 0.0074 | p<0.05 |
| 13 | ANOVA | 0.5931 | <.0001 | 1 vs. 0 | <.0001 | p<0.05 |
|  |  |  |  | 2 vs. 0 | <.0001 | p<0.05 |
|  |  |  |  | 3 vs. 0 | <.0001 | p<0.05 |
|  |  |  |  | 4 vs. 0 | <.0001 | p<0.05 |
|  |  |  |  | 5 vs. 0 | <.0001 | p<0.05 |
|  |  |  |  | 6 vs. 0 | <.0001 | p<0.05 |
|  |  |  |  | 7 vs. 0 | <.0001 | p<0.05 |
|  |  |  |  | 8 vs. 0 | 0.0001 | p<0.05 |

| **Day** | **Test** | **Normality p** | **Main p** | **Comparison** | **Pairwise p** | **Significance** |
| --- | --- | --- | --- | --- | --- | --- |
| 14 | ANOVA | 0.0262 | <.0001 | 1 vs. 0 | <.0001 | p<0.05 |
|  |  |  |  | 2 vs. 0 | <.0001 | p<0.05 |
|  |  |  |  | 3 vs. 0 | <.0001 | p<0.05 |
|  |  |  |  | 4 vs. 0 | <.0001 | p<0.05 |
|  |  |  |  | 5 vs. 0 | <.0001 | p<0.05 |
|  |  |  |  | 6 vs. 0 | <.0001 | p<0.05 |
|  |  |  |  | 7 vs. 0 | <.0001 | p<0.05 |
|  |  |  |  | 8 vs. 0 | 0.0009 | p<0.05 |
| 15 | ANOVA | 0.0055 | <.0001 | 1 vs. 0 | <.0001 | p<0.05 |
|  |  |  |  | 2 vs. 0 | <.0001 | p<0.05 |
|  |  |  |  | 3 vs. 0 | <.0001 | p<0.05 |
|  |  |  |  | 4 vs. 0 | <.0001 | p<0.05 |
|  |  |  |  | 5 vs. 0 | <.0001 | p<0.05 |
|  |  |  |  | 6 vs. 0 | <.0001 | p<0.05 |
|  |  |  |  | 7 vs. 0 | <.0001 | p<0.05 |
|  |  |  |  | 8 vs. 0 | <.0001 | p<0.05 |
| 16 | ANOVA | 0.0139 | <.0001 | 1 vs. 0 | <.0001 | p<0.05 |
|  |  |  |  | 2 vs. 0 | <.0001 | p<0.05 |
|  |  |  |  | 3 vs. 0 | <.0001 | p<0.05 |
|  |  |  |  | 4 vs. 0 | <.0001 | p<0.05 |
|  |  |  |  | 5 vs. 0 | <.0001 | p<0.05 |
|  |  |  |  | 6 vs. 0 | <.0001 | p<0.05 |
|  |  |  |  | 7 vs. 0 | <.0001 | p<0.05 |
|  |  |  |  | 8 vs. 0 | <.0001 | p<0.05 |
| 17 | ANOVA | 0.0297 | <.0001 | 1 vs. 0 | <.0001 | p<0.05 |
|  |  |  |  | 2 vs. 0 | <.0001 | p<0.05 |

| **Day** | **Test** | **Normality p** | **Main p** | **Comparison** | **Pairwise p** | **Significance** |
| --- | --- | --- | --- | --- | --- | --- |
|  |  |  |  | 3 vs. 0 | <.0001 | p<0.05 |
|  |  |  |  | 4 vs. 0 | <.0001 | p<0.05 |
|  |  |  |  | 5 vs. 0 | <.0001 | p<0.05 |
|  |  |  |  | 6 vs. 0 | <.0001 | p<0.05 |
|  |  |  |  | 7 vs. 0 | <.0001 | p<0.05 |
|  |  |  |  | 8 vs. 0 | 0.0007 | p<0.05 |
| 18 | Kruskal-Wallis | 0.0003 | 0.0002 | 1 vs. 0 | 0.2412 | ns |
|  |  |  |  | 2 vs. 0 | 0.2412 | ns |
|  |  |  |  | 3 vs. 0 | 0.2412 | ns |
|  |  |  |  | 4 vs. 0 | 0.2098 | ns |
|  |  |  |  | 5 vs. 0 | 0.2412 | ns |
|  |  |  |  | 6 vs. 0 | 0.2412 | ns |
|  |  |  |  | 7 vs. 0 | 0.2412 | ns |
|  |  |  |  | 8 vs. 0 | 0.2412 | ns |
| 19 | Kruskal-Wallis | 0.0001 | <.0001 | 1 vs. 0 | 0.2577 | ns |
|  |  |  |  | 2 vs. 0 | 0.2577 | ns |
|  |  |  |  | 3 vs. 0 | 0.2373 | ns |
|  |  |  |  | 4 vs. 0 | 0.2112 | ns |
|  |  |  |  | 5 vs. 0 | 0.2577 | ns |
|  |  |  |  | 6 vs. 0 | 0.2577 | ns |
|  |  |  |  | 7 vs. 0 | 0.2577 | ns |
|  |  |  |  | 8 vs. 0 | 0.2577 | ns |
| 20 | Kruskal-Wallis | 0.0001 | 0.0001 | 1 vs. 0 | 0.2456 | ns |
|  |  |  |  | 2 vs. 0 | 0.2351 | ns |
|  |  |  |  | 3 vs. 0 | 0.2334 | ns |
|  |  |  |  | 4 vs. 0 | 0.2030 | ns |

| **Day** | **Test** | **Normality p** | **Main p** | **Comparison** | **Pairwise p** | **Significance** |
| --- | --- | --- | --- | --- | --- | --- |
|  |  |  |  | 5 vs. 0 | 0.3507 | ns |
|  |  |  |  | 6 vs. 0 | 0.3507 | ns |
|  |  |  |  | 7 vs. 0 | 0.3507 | ns |
|  |  |  |  | 8 vs. 0 | 0.5637 | ns |
| 21 | Kruskal-Wallis | 0.0001 | 0.0003 | 1 vs. 0 | 0.3014 | ns |
|  |  |  |  | 2 vs. 0 | 0.2501 | ns |
|  |  |  |  | 3 vs. 0 | 0.2373 | ns |
|  |  |  |  | 4 vs. 0 | 0.1496 | ns |
|  |  |  |  | 5 vs. 0 | 0.3014 | ns |
|  |  |  |  | 6 vs. 0 | 0.3014 | ns |
|  |  |  |  | 7 vs. 0 | 0.3014 | ns |
|  |  |  |  | 8 vs. 0 | 0.3014 | ns |
| 22 | Kruskal-Wallis | 0.0001 | 0.0021 | 1 vs. 0 | 0.2827 | ns |
|  |  |  |  | 2 vs. 0 | 0.2577 | ns |
|  |  |  |  | 3 vs. 0 | 0.2412 | ns |
|  |  |  |  | 4 vs. 0 | 0.2152 | ns |
|  |  |  |  | 5 vs. 0 | 0.3596 | ns |
|  |  |  |  | 6 vs. 0 | 0.2827 | ns |
|  |  |  |  | 7 vs. 0 | 0.3596 | ns |
|  |  |  |  | 8 vs. 0 | 0.2827 | ns |
| 23 | Kruskal-Wallis | 0.0001 | 0.0020 | 1 vs. 0 | 0.2935 | ns |
|  |  |  |  | 2 vs. 0 | 0.2577 | ns |
|  |  |  |  | 3 vs. 0 | 0.2142 | ns |
|  |  |  |  | 4 vs. 0 | 0.2057 | ns |
|  |  |  |  | 5 vs. 0 | 0.2935 | ns |
|  |  |  |  | 6 vs. 0 | 0.2935 | ns |

| **Day** | **Test** | **Normality p** | **Main p** | **Comparison** | **Pairwise p** | **Significance** |
| --- | --- | --- | --- | --- | --- | --- |
|  |  |  |  | 7 vs. 0 | 0.2935 | ns |
|  |  |  |  | 8 vs. 0 | 0.2935 | ns |
| 24 | Kruskal-Wallis | 0.0001 | 0.0125 | 1 vs. 0 | 0.3014 | ns |
|  |  |  |  | 2 vs. 0 | 0.2425 | ns |
|  |  |  |  | 3 vs. 0 | 0.2098 | ns |
|  |  |  |  | 4 vs. 0 | 0.2098 | ns |
|  |  |  |  | 5 vs. 0 | 0.3014 | ns |
|  |  |  |  | 6 vs. 0 | 0.3014 | ns |
|  |  |  |  | 7 vs. 0 | 0.3014 | ns |
|  |  |  |  | 8 vs. 0 | 0.3014 | ns |
| 25 | Kruskal-Wallis | 0.0001 | 0.0249 | 1 vs. 0 | 0.2456 | ns |
|  |  |  |  | 2 vs. 0 | 0.2425 | ns |
|  |  |  |  | 3 vs. 0 | 0.2142 | ns |
|  |  |  |  | 4 vs. 0 | 0.1756 | ns |
|  |  |  |  | 5 vs. 0 | 0.2749 | ns |
|  |  |  |  | 6 vs. 0 | 0.2749 | ns |
|  |  |  |  | 7 vs. 0 | 0.2749 | ns |
|  |  |  |  | 8 vs. 0 | 0.2456 | ns |
| 26 | Kruskal-Wallis | 0.0001 | 0.0286 | 1 vs. 0 | 0.2577 | ns |
|  |  |  |  | 2 vs. 0 | 0.2577 | ns |
|  |  |  |  | 3 vs. 0 | 0.1774 | ns |
|  |  |  |  | 4 vs. 0 | 0.1768 | ns |
|  |  |  |  | 5 vs. 0 | 0.2577 | ns |
|  |  |  |  | 6 vs. 0 | 0.2577 | ns |
|  |  |  |  | 7 vs. 0 | 0.2577 | ns |
|  |  |  |  | 8 vs. 0 | 0.2577 | ns |

| **Day** | **Test** | **Normality p** | **Main p** | **Comparison** | **Pairwise p** | **Significance** |
| --- | --- | --- | --- | --- | --- | --- |
| 27 | Kruskal-Wallis | 0.0001 | 0.0110 | 1 vs. 0 | 0.2351 | ns |
|  |  |  |  | 2 vs. 0 | 0.2351 | ns |
|  |  |  |  | 3 vs. 0 | 0.2029 | ns |
|  |  |  |  | 4 vs. 0 | 0.1859 | ns |
|  |  |  |  | 5 vs. 0 | 0.2351 | ns |
|  |  |  |  | 6 vs. 0 | 0.2351 | ns |
|  |  |  |  | 7 vs. 0 | 0.2351 | ns |
|  |  |  |  | 8 vs. 0 | 0.2351 | ns |
| 28 | ANOVA | 0.0076 | 0.0016 | 1 vs. 0 | <.0001 | p<0.05 |
|  |  |  |  | 2 vs. 0 | <.0001 | p<0.05 |
|  |  |  |  | 3 vs. 0 | <.0001 | p<0.05 |
|  |  |  |  | 4 vs. 0 | <.0001 | p<0.05 |
|  |  |  |  | 5 vs. 0 | 0.0004 | p<0.05 |
|  |  |  |  | 6 vs. 0 | 0.0014 | p<0.05 |
|  |  |  |  | 7 vs. 0 | 0.0001 | p<0.05 |
|  |  |  |  | 8 vs. 0 | 0.0422 | p<0.05 |
| 29 | Kruskal-Wallis | 0.0008 | 0.0408 | 1 vs. 0 | 0.2927 | ns |
|  |  |  |  | 2 vs. 0 | 0.2927 | ns |
|  |  |  |  | 3 vs. 0 | 0.2801 | ns |
|  |  |  |  | 4 vs. 0 | 0.2927 | ns |
|  |  |  |  | 5 vs. 0 | 0.2927 | ns |
|  |  |  |  | 6 vs. 0 | 0.2927 | ns |
|  |  |  |  | 7 vs. 0 | 0.2927 | ns |
|  |  |  |  | 8 vs. 0 | 0.2927 | ns |
| 30 | ANOVA | 0.0119 | <.0001 | 1 vs. 0 | <.0001 | p<0.05 |
|  |  |  |  | 2 vs. 0 | <.0001 | p<0.05 |

| **Day** | **Test** | **Normality p** | **Main p** | **Comparison** | **Pairwise p** | **Significance** |
| --- | --- | --- | --- | --- | --- | --- |
|  |  |  |  | 3 vs. 0 | <.0001 | p<0.05 |
|  |  |  |  | 4 vs. 0 | <.0001 | p<0.05 |
|  |  |  |  | 5 vs. 0 | <.0001 | p<0.05 |
|  |  |  |  | 6 vs. 0 | <.0001 | p<0.05 |
|  |  |  |  | 7 vs. 0 | <.0001 | p<0.05 |
|  |  |  |  | 8 vs. 0 | 0.0001 | p<0.05 |
| 31 | ANOVA | 0.0025 | 0.0007 | 1 vs. 0 | <.0001 | p<0.05 |
|  |  |  |  | 2 vs. 0 | 0.0001 | p<0.05 |
|  |  |  |  | 3 vs. 0 | <.0001 | p<0.05 |
|  |  |  |  | 4 vs. 0 | <.0001 | p<0.05 |
|  |  |  |  | 5 vs. 0 | 0.0001 | p<0.05 |
|  |  |  |  | 6 vs. 0 | 0.0143 | p<0.05 |
|  |  |  |  | 7 vs. 0 | 0.0002 | p<0.05 |
|  |  |  |  | 8 vs. 0 | 0.0056 | p<0.05 |
| 32 | ANOVA | 0.0075 | 0.0008 | 1 vs. 0 | <.0001 | p<0.05 |
|  |  |  |  | 2 vs. 0 | <.0001 | p<0.05 |
|  |  |  |  | 3 vs. 0 | <.0001 | p<0.05 |
|  |  |  |  | 4 vs. 0 | <.0001 | p<0.05 |
|  |  |  |  | 5 vs. 0 | 0.0002 | p<0.05 |
|  |  |  |  | 6 vs. 0 | 0.0079 | p<0.05 |
|  |  |  |  | 7 vs. 0 | 0.0023 | p<0.05 |
|  |  |  |  | 8 vs. 0 | 0.0181 | p<0.05 |
| 33 | ANOVA | 0.0013 | 0.0046 | 1 vs. 0 | 0.0006 | p<0.05 |
|  |  |  |  | 2 vs. 0 | <.0001 | p<0.05 |
|  |  |  |  | 3 vs. 0 | 0.0003 | p<0.05 |
|  |  |  |  | 4 vs. 0 | 0.0002 | p<0.05 |

| **Day** | **Test** | **Normality p** | **Main p** | **Comparison** | **Pairwise p** | **Significance** |
| --- | --- | --- | --- | --- | --- | --- |
|  |  |  |  | 5 vs. 0 | 0.0002 | p<0.05 |
|  |  |  |  | 6 vs. 0 | 0.0178 | p<0.05 |
|  |  |  |  | 7 vs. 0 | 0.0017 | p<0.05 |
|  |  |  |  | 8 vs. 0 | 0.0280 | p<0.05 |
| 34 | ANOVA | 0.0353 | 0.0002 | 1 vs. 0 | 0.0001 | p<0.05 |
|  |  |  |  | 2 vs. 0 | <.0001 | p<0.05 |
|  |  |  |  | 3 vs. 0 | <.0001 | p<0.05 |
|  |  |  |  | 4 vs. 0 | <.0001 | p<0.05 |
|  |  |  |  | 5 vs. 0 | 0.0002 | p<0.05 |
|  |  |  |  | 6 vs. 0 | 0.0255 | p<0.05 |
|  |  |  |  | 7 vs. 0 | 0.0092 | p<0.05 |
|  |  |  |  | 8 vs. 0 | 0.0278 | p<0.05 |
| 35 | ANOVA | 0.0014 | 0.0002 | 1 vs. 0 | <.0001 | p<0.05 |
|  |  |  |  | 2 vs. 0 | <.0001 | p<0.05 |
|  |  |  |  | 3 vs. 0 | <.0001 | p<0.05 |
|  |  |  |  | 4 vs. 0 | <.0001 | p<0.05 |
|  |  |  |  | 5 vs. 0 | <.0001 | p<0.05 |
|  |  |  |  | 6 vs. 0 | 0.0014 | p<0.05 |
|  |  |  |  | 7 vs. 0 | 0.0003 | p<0.05 |
|  |  |  |  | 8 vs. 0 | 0.0028 | p<0.05 |
| 36 | ANOVA | 0.2512 | 0.0224 | 1 vs. 0 | 0.0742 | ns |
|  |  |  |  | 2 vs. 0 | 0.0247 | p<0.05 |
|  |  |  |  | 3 vs. 0 | 0.0090 | p<0.05 |
|  |  |  |  | 4 vs. 0 | 0.0094 | p<0.05 |
|  |  |  |  | 5 vs. 0 | 0.0201 | p<0.05 |
|  |  |  |  | 6 vs. 0 | 0.2290 | ns |

| **Day** | **Test** | **Normality p** | **Main p** | **Comparison** | **Pairwise p** | **Significance** |
| --- | --- | --- | --- | --- | --- | --- |
|  |  |  |  | 7 vs. 0 | 0.5489 | ns |
|  |  |  |  | 8 vs. 0 | 0.1499 | ns |
| 37 | ANOVA | 0.5435 | <.0001 | 1 vs. 0 | <.0001 | p<0.05 |
|  |  |  |  | 2 vs. 0 | <.0001 | p<0.05 |
|  |  |  |  | 3 vs. 0 | <.0001 | p<0.05 |
|  |  |  |  | 4 vs. 0 | <.0001 | p<0.05 |
|  |  |  |  | 5 vs. 0 | <.0001 | p<0.05 |
|  |  |  |  | 6 vs. 0 | 0.0004 | p<0.05 |
|  |  |  |  | 7 vs. 0 | 0.0007 | p<0.05 |
|  |  |  |  | 8 vs. 0 | 0.0012 | p<0.05 |
| 38 | ANOVA | 0.0018 | 0.0021 | 1 vs. 0 | 0.0012 | p<0.05 |
|  |  |  |  | 2 vs. 0 | 0.0011 | p<0.05 |
|  |  |  |  | 3 vs. 0 | 0.0001 | p<0.05 |
|  |  |  |  | 4 vs. 0 | <.0001 | p<0.05 |
|  |  |  |  | 5 vs. 0 | 0.0008 | p<0.05 |
|  |  |  |  | 6 vs. 0 | 0.0091 | p<0.05 |
|  |  |  |  | 7 vs. 0 | 0.0074 | p<0.05 |
|  |  |  |  | 8 vs. 0 | 0.0660 | ns |
| 39 | Kruskal-Wallis | 0.0006 | 0.0536 | 1 vs. 0 | 0.2351 | ns |
|  |  |  |  | 2 vs. 0 | 0.2351 | ns |
|  |  |  |  | 3 vs. 0 | 0.1859 | ns |
|  |  |  |  | 4 vs. 0 | 0.1859 | ns |
|  |  |  |  | 5 vs. 0 | 0.2351 | ns |
|  |  |  |  | 6 vs. 0 | 0.2351 | ns |
|  |  |  |  | 7 vs. 0 | 0.2351 | ns |
|  |  |  |  | 8 vs. 0 | 0.2351 | ns |

| **Day** | **Test** | **Normality p** | **Main p** | **Comparison** | **Pairwise p** | **Significance** |
| --- | --- | --- | --- | --- | --- | --- |
| 40 | ANOVA | 0.0018 | 0.0099 | 1 vs. 0 | 0.0006 | p<0.05 |
|  |  |  |  | 2 vs. 0 | 0.0014 | p<0.05 |
|  |  |  |  | 3 vs. 0 | 0.0003 | p<0.05 |
|  |  |  |  | 4 vs. 0 | 0.0001 | p<0.05 |
|  |  |  |  | 5 vs. 0 | 0.0004 | p<0.05 |
|  |  |  |  | 6 vs. 0 | 0.0014 | p<0.05 |
|  |  |  |  | 7 vs. 0 | 0.0122 | p<0.05 |
|  |  |  |  | 8 vs. 0 | 0.0155 | p<0.05 |
| 41 | ANOVA | 0.0034 | 0.0378 | 1 vs. 0 | 0.0044 | p<0.05 |
|  |  |  |  | 2 vs. 0 | 0.0261 | p<0.05 |
|  |  |  |  | 3 vs. 0 | 0.0009 | p<0.05 |
|  |  |  |  | 4 vs. 0 | 0.0036 | p<0.05 |
|  |  |  |  | 5 vs. 0 | 0.0016 | p<0.05 |
|  |  |  |  | 6 vs. 0 | 0.0161 | p<0.05 |
|  |  |  |  | 7 vs. 0 | 0.0320 | p<0.05 |
|  |  |  |  | 8 vs. 0 | 0.1031 | ns |
| 42 | ANOVA | 0.0016 | 0.0170 | 1 vs. 0 | 0.0024 | p<0.05 |
|  |  |  |  | 2 vs. 0 | 0.0034 | p<0.05 |
|  |  |  |  | 3 vs. 0 | 0.0004 | p<0.05 |
|  |  |  |  | 4 vs. 0 | 0.0037 | p<0.05 |
|  |  |  |  | 5 vs. 0 | 0.0034 | p<0.05 |
|  |  |  |  | 6 vs. 0 | 0.0250 | p<0.05 |
|  |  |  |  | 7 vs. 0 | 0.0510 | ns |
|  |  |  |  | 8 vs. 0 | 0.0937 | ns |
| 43 | ANOVA | 0.0133 | 0.0050 | 1 vs. 0 | 0.0002 | p<0.05 |
|  |  |  |  | 2 vs. 0 | 0.0004 | p<0.05 |

| **Day** | **Test** | **Normality p** | **Main p** | **Comparison** | **Pairwise p** | **Significance** |
| --- | --- | --- | --- | --- | --- | --- |
|  |  |  |  | 3 vs. 0 | <.0001 | p<0.05 |
|  |  |  |  | 4 vs. 0 | <.0001 | p<0.05 |
|  |  |  |  | 5 vs. 0 | 0.0010 | p<0.05 |
|  |  |  |  | 6 vs. 0 | 0.0015 | p<0.05 |
|  |  |  |  | 7 vs. 0 | 0.0029 | p<0.05 |
|  |  |  |  | 8 vs. 0 | 0.0110 | p<0.05 |
| 44 | Kruskal-Wallis | 0.0001 | 0.1739 | 1 vs. 0 | 0.3923 | ns |
|  |  |  |  | 2 vs. 0 | 0.3923 | ns |
|  |  |  |  | 3 vs. 0 | 0.3923 | ns |
|  |  |  |  | 4 vs. 0 | 0.3454 | ns |
|  |  |  |  | 5 vs. 0 | 0.3923 | ns |
|  |  |  |  | 6 vs. 0 | 0.3923 | ns |
|  |  |  |  | 7 vs. 0 | 0.3923 | ns |
|  |  |  |  | 8 vs. 0 | 0.3923 | ns |
| 45 | Kruskal-Wallis | 0.0001 | 0.4335 | 1 vs. 0 | 0.3971 | ns |
|  |  |  |  | 2 vs. 0 | 0.3971 | ns |
|  |  |  |  | 3 vs. 0 | 0.3971 | ns |
|  |  |  |  | 4 vs. 0 | 0.3971 | ns |

| **Day** | **Test** | **Normality p** | **Main p** | **Comparison** | **Pairwise p** | **Significance** |
| --- | --- | --- | --- | --- | --- | --- |
| 3 | Kruskal-Wallis | 0.0001 | 0.7473 | 1 vs. 0 | 1.0000 | ns |
|  |  |  |  | 2 vs. 0 | 1.0000 | ns |
|  |  |  |  | 3 vs. 0 | 1.0000 | ns |
|  |  |  |  | 4 vs. 0 | 1.0000 | ns |
|  |  |  |  | 5 vs. 0 | 1.0000 | ns |
|  |  |  |  | 6 vs. 0 | 1.0000 | ns |
|  |  |  |  | 7 vs. 0 | 1.0000 | ns |
|  |  |  |  | 8 vs. 0 | 1.0000 | ns |
| 4 | ANOVA | 0.0029 | 0.3034 | 4 vs. 0 | 0.0926 | ns |
|  |  |  |  | 5 vs. 0 | 0.9162 | ns |
|  |  |  |  | 6 vs. 0 | 0.2135 | ns |
|  |  |  |  | 7 vs. 0 | 0.9162 | ns |
|  |  |  |  | 8 vs. 0 | 0.9162 | ns |
| 5 | Kruskal-Wallis | 0.0001 | 0.9172 | 1 vs. 0 | 1.0000 | ns |
|  |  |  |  | 2 vs. 0 | 1.0000 | ns |
|  |  |  |  | 3 vs. 0 | 1.0000 | ns |
|  |  |  |  | 4 vs. 0 | 1.0000 | ns |
|  |  |  |  | 5 vs. 0 | 1.0000 | ns |
|  |  |  |  | 6 vs. 0 | 1.0000 | ns |
|  |  |  |  | 7 vs. 0 | 1.0000 | ns |
|  |  |  |  | 8 vs. 0 | 1.0000 | ns |
| 6 | Kruskal-Wallis | 0.0001 | 0.0587 | 1 vs. 0 | 1.0000 | ns |
|  |  |  |  | 2 vs. 0 | 1.0000 | ns |
|  |  |  |  | 3 vs. 0 | 1.0000 | ns |
|  |  |  |  | 4 vs. 0 | 1.0000 | ns |
|  |  |  |  | 5 vs. 0 | 1.0000 | ns |

| **Day** | **Test** | **Normality p** | **Main p** | **Comparison** | **Pairwise p** | **Significance** |
| --- | --- | --- | --- | --- | --- | --- |
|  |  |  |  | 6 vs. 0 | 1.0000 | ns |
|  |  |  |  | 7 vs. 0 | 1.0000 | ns |
|  |  |  |  | 8 vs. 0 | 0.1157 | ns |
| 7 | Kruskal-Wallis | 0.0001 | 0.1606 | 1 vs. 0 | 1.0000 | ns |
|  |  |  |  | 2 vs. 0 | 1.0000 | ns |
|  |  |  |  | 3 vs. 0 | 0.7018 | ns |
|  |  |  |  | 4 vs. 0 | 1.0000 | ns |
|  |  |  |  | 5 vs. 0 | 1.0000 | ns |
|  |  |  |  | 6 vs. 0 | 1.0000 | ns |
|  |  |  |  | 7 vs. 0 | 1.0000 | ns |
|  |  |  |  | 8 vs. 0 | 1.0000 | ns |
| 8 | Kruskal-Wallis | 0.0001 | 0.0506 | 1 vs. 0 | 1.0000 | ns |
|  |  |  |  | 2 vs. 0 | 1.0000 | ns |
|  |  |  |  | 3 vs. 0 | 0.4955 | ns |
|  |  |  |  | 4 vs. 0 | 0.8996 | ns |
|  |  |  |  | 5 vs. 0 | 1.0000 | ns |
|  |  |  |  | 6 vs. 0 | 1.0000 | ns |
|  |  |  |  | 7 vs. 0 | 1.0000 | ns |
|  |  |  |  | 8 vs. 0 | 1.0000 | ns |
| 9 | Kruskal-Wallis | 0.0001 | 0.4629 | 1 vs. 0 | 0.6183 | ns |
|  |  |  |  | 2 vs. 0 | 0.6183 | ns |
|  |  |  |  | 3 vs. 0 | 0.6183 | ns |
|  |  |  |  | 4 vs. 0 | 0.6183 | ns |
| 10 | Kruskal-Wallis | 0.0001 | 0.0301 | 1 vs. 0 | 0.6232 | ns |
|  |  |  |  | 2 vs. 0 | 0.5456 | ns |
|  |  |  |  | 3 vs. 0 | 0.5456 | ns |

| **Day** | **Test** | **Normality p** | **Main p** | **Comparison** | **Pairwise p** | **Significance** |
| --- | --- | --- | --- | --- | --- | --- |
|  |  |  |  | 4 vs. 0 | 0.6232 | ns |
|  |  |  |  | 5 vs. 0 | 1.0000 | ns |
|  |  |  |  | 6 vs. 0 | 0.6232 | ns |
|  |  |  |  | 7 vs. 0 | 1.0000 | ns |
|  |  |  |  | 8 vs. 0 | 1.0000 | ns |
| 12 | Kruskal-Wallis | 0.0001 | 0.8101 | 4 vs. 0 | 1.0000 | ns |
|  |  |  |  | 5 vs. 0 | 1.0000 | ns |
|  |  |  |  | 6 vs. 0 | 1.0000 | ns |
|  |  |  |  | 7 vs. 0 | 1.0000 | ns |
|  |  |  |  | 8 vs. 0 | 1.0000 | ns |
| 14 | Kruskal-Wallis | 0.0001 | 0.0011 | 1 vs. 0 | 1.0000 | ns |
|  |  |  |  | 2 vs. 0 | 1.0000 | ns |
|  |  |  |  | 3 vs. 0 | 1.0000 | ns |
|  |  |  |  | 4 vs. 0 | 1.0000 | ns |
|  |  |  |  | 5 vs. 0 | 1.0000 | ns |
|  |  |  |  | 6 vs. 0 | 1.0000 | ns |
|  |  |  |  | 7 vs. 0 | 1.0000 | ns |
|  |  |  |  | 8 vs. 0 | 1.0000 | ns |
| 16 | Kruskal-Wallis | 0.0001 | 0.1259 | 4 vs. 0 | 0.2275 | ns |
|  |  |  |  | 5 vs. 0 | 1.0000 | ns |
|  |  |  |  | 6 vs. 0 | 1.0000 | ns |
|  |  |  |  | 7 vs. 0 | 1.0000 | ns |
|  |  |  |  | 8 vs. 0 | 1.0000 | ns |
| 18 | Kruskal-Wallis | 0.0001 | 0.3070 | 4 vs. 0 | 0.2275 | ns |
|  |  |  |  | 5 vs. 0 | 1.0000 | ns |
|  |  |  |  | 6 vs. 0 | 1.0000 | ns |

| **Day** | **Test** | **Normality p** | **Main p** | **Comparison** | **Pairwise p** | **Significance** |
| --- | --- | --- | --- | --- | --- | --- |
|  |  |  |  | 7 vs. 0 | 1.0000 | ns |
|  |  |  |  | 8 vs. 0 | 1.0000 | ns |
| 20 | Kruskal-Wallis | 0.0001 | 0.8653 | 4 vs. 0 | 1.0000 | ns |
|  |  |  |  | 5 vs. 0 | 1.0000 | ns |
|  |  |  |  | 6 vs. 0 | 1.0000 | ns |
|  |  |  |  | 7 vs. 0 | 1.0000 | ns |
|  |  |  |  | 8 vs. 0 | 1.0000 | ns |
| 21 | Kruskal-Wallis | 0.0001 | <.0001 | 1 vs. 0 | 0.0455 | p<0.05 |
|  |  |  |  | 2 vs. 0 | 0.0286 | p<0.05 |
|  |  |  |  | 3 vs. 0 | 0.0187 | p<0.05 |
|  |  |  |  | 4 vs. 0 | 0.0187 | p<0.05 |
| 28 | Kruskal-Wallis | 0.0001 | 0.0535 | 1 vs. 0 | 0.6292 | ns |
|  |  |  |  | 2 vs. 0 | 0.4996 | ns |
|  |  |  |  | 3 vs. 0 | 0.3185 | ns |
|  |  |  |  | 4 vs. 0 | 0.1144 | ns |
|  |  |  |  | 5 vs. 0 | 0.6292 | ns |
|  |  |  |  | 6 vs. 0 | 0.6292 | ns |
|  |  |  |  | 7 vs. 0 | 0.4996 | ns |
|  |  |  |  | 8 vs. 0 | 0.6292 | ns |
| 35 | Kruskal-Wallis | 0.0001 | 0.0019 | 1 vs. 0 | 0.4996 | ns |
|  |  |  |  | 2 vs. 0 | 0.4996 | ns |
|  |  |  |  | 3 vs. 0 | 0.3185 | ns |
|  |  |  |  | 4 vs. 0 | 0.1144 | ns |
|  |  |  |  | 5 vs. 0 | 0.4996 | ns |
|  |  |  |  | 6 vs. 0 | 0.4996 | ns |
|  |  |  |  | 7 vs. 0 | 0.4996 | ns |

| **Day** | **Test** | **Normality p** | **Main p** | **Comparison** | **Pairwise p** | **Significance** |
| --- | --- | --- | --- | --- | --- | --- |
|  |  |  |  | 8 vs. 0 | 0.4996 | ns |
| 45 | Kruskal-Wallis | 0.0001 | <.0001 | 1 vs. 0 | 0.0455 | p<0.05 |
|  |  |  |  | 2 vs. 0 | 0.0286 | p<0.05 |
|  |  |  |  | 3 vs. 0 | 0.0187 | p<0.05 |
|  |  |  |  | 4 vs. 0 | 0.0187 | p<0.05 |

| **Day** | **Test** | **Normality p** | **Main p** | **Comparison** | **Pairwise p** | **Significance** |
| --- | --- | --- | --- | --- | --- | --- |
| 0 | ANOVA | 0.1694 | 0.6626 | 1 vs. 0 | 0.0623 | ns |
|  |  |  |  | 2 vs. 0 | 0.6105 | ns |
|  |  |  |  | 3 vs. 0 | 0.3914 | ns |
|  |  |  |  | 4 vs. 0 | 0.3751 | ns |
|  |  |  |  | 5 vs. 0 | 0.2944 | ns |
|  |  |  |  | 6 vs. 0 | 0.3662 | ns |
|  |  |  |  | 7 vs. 0 | 0.5936 | ns |
|  |  |  |  | 8 vs. 0 | 0.7483 | ns |
| 1 | ANOVA | 0.0757 | 0.1241 | 1 vs. 0 | 0.1037 | ns |
|  |  |  |  | 2 vs. 0 | 0.1556 | ns |
|  |  |  |  | 3 vs. 0 | 0.3210 | ns |
|  |  |  |  | 4 vs. 0 | 0.6279 | ns |
|  |  |  |  | 5 vs. 0 | 0.4905 | ns |
|  |  |  |  | 6 vs. 0 | 0.3140 | ns |
|  |  |  |  | 7 vs. 0 | 0.6080 | ns |
|  |  |  |  | 8 vs. 0 | 0.5495 | ns |
| 2 | ANOVA | 0.9662 | 0.1995 | 1 vs. 0 | 0.0260 | p<0.05 |
|  |  |  |  | 2 vs. 0 | 0.2645 | ns |
|  |  |  |  | 3 vs. 0 | 0.4223 | ns |
|  |  |  |  | 4 vs. 0 | 0.4770 | ns |
|  |  |  |  | 5 vs. 0 | 0.5353 | ns |
|  |  |  |  | 6 vs. 0 | 0.6024 | ns |
|  |  |  |  | 7 vs. 0 | 0.4481 | ns |
|  |  |  |  | 8 vs. 0 | 0.8290 | ns |
| 3 | ANOVA | 0.4537 | 0.0754 | 1 vs. 0 | 0.0438 | p<0.05 |
|  |  |  |  | 2 vs. 0 | 0.0747 | ns |

| **Day** | **Test** | **Normality p** | **Main p** | **Comparison** | **Pairwise p** | **Significance** |
| --- | --- | --- | --- | --- | --- | --- |
|  |  |  |  | 3 vs. 0 | 0.4125 | ns |
|  |  |  |  | 4 vs. 0 | 0.8402 | ns |
|  |  |  |  | 5 vs. 0 | 0.2588 | ns |
|  |  |  |  | 6 vs. 0 | 0.6217 | ns |
|  |  |  |  | 7 vs. 0 | 0.7447 | ns |
|  |  |  |  | 8 vs. 0 | 0.8945 | ns |
| 4 | ANOVA | 0.7954 | 0.0005 | 1 vs. 0 | 0.0223 | p<0.05 |
|  |  |  |  | 2 vs. 0 | 0.0771 | ns |
|  |  |  |  | 3 vs. 0 | 0.1593 | ns |
|  |  |  |  | 4 vs. 0 | 0.7966 | ns |
|  |  |  |  | 5 vs. 0 | 0.1078 | ns |
|  |  |  |  | 6 vs. 0 | 0.2313 | ns |
|  |  |  |  | 7 vs. 0 | 0.4671 | ns |
|  |  |  |  | 8 vs. 0 | 0.0377 | p<0.05 |
| 5 | ANOVA | 0.9946 | <.0001 | 1 vs. 0 | 0.0007 | p<0.05 |
|  |  |  |  | 2 vs. 0 | 0.0005 | p<0.05 |
|  |  |  |  | 3 vs. 0 | 0.0053 | p<0.05 |
|  |  |  |  | 4 vs. 0 | 0.0958 | ns |
|  |  |  |  | 5 vs. 0 | 0.7294 | ns |
|  |  |  |  | 6 vs. 0 | 0.5865 | ns |
|  |  |  |  | 7 vs. 0 | 0.5005 | ns |
|  |  |  |  | 8 vs. 0 | 0.4875 | ns |
| 6 | ANOVA | 0.7775 | <.0001 | 1 vs. 0 | <.0001 | p<0.05 |
|  |  |  |  | 2 vs. 0 | <.0001 | p<0.05 |
|  |  |  |  | 3 vs. 0 | <.0001 | p<0.05 |
|  |  |  |  | 4 vs. 0 | 0.0038 | p<0.05 |

| **Day** | **Test** | **Normality p** | **Main p** | **Comparison** | **Pairwise p** | **Significance** |
| --- | --- | --- | --- | --- | --- | --- |
|  |  |  |  | 5 vs. 0 | 0.9288 | ns |
|  |  |  |  | 6 vs. 0 | 0.8669 | ns |
|  |  |  |  | 7 vs. 0 | 0.4605 | ns |
|  |  |  |  | 8 vs. 0 | 0.9312 | ns |
| 7 | ANOVA | 0.7180 | <.0001 | 1 vs. 0 | <.0001 | p<0.05 |
|  |  |  |  | 2 vs. 0 | <.0001 | p<0.05 |
|  |  |  |  | 3 vs. 0 | <.0001 | p<0.05 |
|  |  |  |  | 4 vs. 0 | 0.0017 | p<0.05 |
|  |  |  |  | 5 vs. 0 | 0.7409 | ns |
|  |  |  |  | 6 vs. 0 | 0.4884 | ns |
|  |  |  |  | 7 vs. 0 | 0.8468 | ns |
|  |  |  |  | 8 vs. 0 | 0.8872 | ns |
| 8 | ANOVA | 0.1987 | <.0001 | 1 vs. 0 | <.0001 | p<0.05 |
|  |  |  |  | 2 vs. 0 | <.0001 | p<0.05 |
|  |  |  |  | 3 vs. 0 | 0.0006 | p<0.05 |
|  |  |  |  | 4 vs. 0 | 0.0142 | p<0.05 |
|  |  |  |  | 5 vs. 0 | 0.4484 | ns |
|  |  |  |  | 6 vs. 0 | 0.1207 | ns |
|  |  |  |  | 7 vs. 0 | 0.5363 | ns |
|  |  |  |  | 8 vs. 0 | 0.4729 | ns |
| 9 | ANOVA | 0.5869 | <.0001 | 1 vs. 0 | <.0001 | p<0.05 |
|  |  |  |  | 2 vs. 0 | <.0001 | p<0.05 |
|  |  |  |  | 3 vs. 0 | 0.0036 | p<0.05 |
|  |  |  |  | 4 vs. 0 | 0.0091 | p<0.05 |
|  |  |  |  | 5 vs. 0 | 0.7920 | ns |
|  |  |  |  | 6 vs. 0 | 0.0901 | ns |

| **Day** | **Test** | **Normality p** | **Main p** | **Comparison** | **Pairwise p** | **Significance** |
| --- | --- | --- | --- | --- | --- | --- |
|  |  |  |  | 7 vs. 0 | 0.6648 | ns |
|  |  |  |  | 8 vs. 0 | 0.7106 | ns |
| -2 | ANOVA | 0.9695 | 0.3367 | 1 vs. 0 | 0.0638 | ns |
|  |  |  |  | 2 vs. 0 | 0.9171 | ns |
|  |  |  |  | 3 vs. 0 | 0.4339 | ns |
|  |  |  |  | 4 vs. 0 | 0.0996 | ns |
|  |  |  |  | 5 vs. 0 | 0.1921 | ns |
|  |  |  |  | 6 vs. 0 | 0.8633 | ns |
|  |  |  |  | 7 vs. 0 | 0.5787 | ns |
|  |  |  |  | 8 vs. 0 | 0.6867 | ns |
| -3 | ANOVA | 0.5464 | 0.3434 | 1 vs. 0 | 0.0056 | p<0.05 |
|  |  |  |  | 2 vs. 0 | 0.4768 | ns |
|  |  |  |  | 3 vs. 0 | 0.3129 | ns |
|  |  |  |  | 4 vs. 0 | 0.4660 | ns |
|  |  |  |  | 5 vs. 0 | 0.5155 | ns |
|  |  |  |  | 6 vs. 0 | 0.6336 | ns |
|  |  |  |  | 7 vs. 0 | 0.4386 | ns |
|  |  |  |  | 8 vs. 0 | 0.6934 | ns |
| -4 | ANOVA | 0.3845 | 0.3165 | 1 vs. 0 | 0.0244 | p<0.05 |
|  |  |  |  | 2 vs. 0 | 0.6093 | ns |
|  |  |  |  | 3 vs. 0 | 0.9390 | ns |
|  |  |  |  | 4 vs. 0 | 0.5110 | ns |
|  |  |  |  | 5 vs. 0 | 0.8735 | ns |
|  |  |  |  | 6 vs. 0 | 0.4942 | ns |
|  |  |  |  | 7 vs. 0 | 0.9180 | ns |
|  |  |  |  | 8 vs. 0 | 0.4337 | ns |

| **Day** | **Test** | **Normality p** | **Main p** | **Comparison** | **Pairwise p** | **Significance** |
| --- | --- | --- | --- | --- | --- | --- |
| -6 | ANOVA | 0.0245 | 0.7544 | 1 vs. 0 | 0.4260 | ns |
|  |  |  |  | 2 vs. 0 | 0.4843 | ns |
|  |  |  |  | 3 vs. 0 | 0.8341 | ns |
|  |  |  |  | 4 vs. 0 | 0.6122 | ns |
|  |  |  |  | 5 vs. 0 | 0.1852 | ns |
|  |  |  |  | 6 vs. 0 | 0.2750 | ns |
|  |  |  |  | 7 vs. 0 | 0.5999 | ns |
|  |  |  |  | 8 vs. 0 | 0.7512 | ns |
| 10 | ANOVA | 0.4843 | <.0001 | 1 vs. 0 | <.0001 | p<0.05 |
|  |  |  |  | 2 vs. 0 | <.0001 | p<0.05 |
|  |  |  |  | 3 vs. 0 | 0.0002 | p<0.05 |
|  |  |  |  | 4 vs. 0 | 0.0004 | p<0.05 |
|  |  |  |  | 5 vs. 0 | 0.4699 | ns |
|  |  |  |  | 6 vs. 0 | 0.6843 | ns |
|  |  |  |  | 7 vs. 0 | 0.6535 | ns |
|  |  |  |  | 8 vs. 0 | 0.4275 | ns |
| 11 | ANOVA | 0.9862 | <.0001 | 1 vs. 0 | <.0001 | p<0.05 |
|  |  |  |  | 2 vs. 0 | <.0001 | p<0.05 |
|  |  |  |  | 3 vs. 0 | <.0001 | p<0.05 |
|  |  |  |  | 4 vs. 0 | 0.0001 | p<0.05 |
|  |  |  |  | 5 vs. 0 | 0.2975 | ns |
|  |  |  |  | 6 vs. 0 | 0.2307 | ns |
|  |  |  |  | 7 vs. 0 | 0.4612 | ns |
|  |  |  |  | 8 vs. 0 | 0.1652 | ns |
| 12 | ANOVA | 0.8425 | <.0001 | 1 vs. 0 | <.0001 | p<0.05 |
|  |  |  |  | 2 vs. 0 | <.0001 | p<0.05 |

| **Day** | **Test** | **Normality p** | **Main p** | **Comparison** | **Pairwise p** | **Significance** |
| --- | --- | --- | --- | --- | --- | --- |
|  |  |  |  | 3 vs. 0 | <.0001 | p<0.05 |
|  |  |  |  | 4 vs. 0 | <.0001 | p<0.05 |
|  |  |  |  | 5 vs. 0 | 0.1434 | ns |
|  |  |  |  | 6 vs. 0 | 0.4145 | ns |
|  |  |  |  | 7 vs. 0 | 0.5724 | ns |
|  |  |  |  | 8 vs. 0 | 0.3793 | ns |
| 13 | ANOVA | 0.8422 | <.0001 | 1 vs. 0 | <.0001 | p<0.05 |
|  |  |  |  | 2 vs. 0 | <.0001 | p<0.05 |
|  |  |  |  | 3 vs. 0 | <.0001 | p<0.05 |
|  |  |  |  | 4 vs. 0 | <.0001 | p<0.05 |
|  |  |  |  | 5 vs. 0 | 0.0622 | ns |
|  |  |  |  | 6 vs. 0 | 0.0709 | ns |
|  |  |  |  | 7 vs. 0 | 0.2234 | ns |
|  |  |  |  | 8 vs. 0 | 0.1680 | ns |
| 14 | ANOVA | 0.2515 | <.0001 | 1 vs. 0 | <.0001 | p<0.05 |
|  |  |  |  | 2 vs. 0 | <.0001 | p<0.05 |
|  |  |  |  | 3 vs. 0 | 0.0009 | p<0.05 |
|  |  |  |  | 4 vs. 0 | 0.0012 | p<0.05 |
|  |  |  |  | 5 vs. 0 | 0.3518 | ns |
|  |  |  |  | 6 vs. 0 | 0.3650 | ns |
|  |  |  |  | 7 vs. 0 | 0.6516 | ns |
|  |  |  |  | 8 vs. 0 | 0.8158 | ns |
| 15 | ANOVA | 0.4694 | <.0001 | 1 vs. 0 | <.0001 | p<0.05 |
|  |  |  |  | 2 vs. 0 | <.0001 | p<0.05 |
|  |  |  |  | 3 vs. 0 | 0.0004 | p<0.05 |
|  |  |  |  | 4 vs. 0 | 0.0006 | p<0.05 |

| **Day** | **Test** | **Normality p** | **Main p** | **Comparison** | **Pairwise p** | **Significance** |
| --- | --- | --- | --- | --- | --- | --- |
|  |  |  |  | 5 vs. 0 | 0.2193 | ns |
|  |  |  |  | 6 vs. 0 | 0.0957 | ns |
|  |  |  |  | 7 vs. 0 | 0.3456 | ns |
|  |  |  |  | 8 vs. 0 | 0.6849 | ns |
| 16 | ANOVA | 0.5671 | <.0001 | 1 vs. 0 | 0.0006 | p<0.05 |
|  |  |  |  | 2 vs. 0 | <.0001 | p<0.05 |
|  |  |  |  | 3 vs. 0 | 0.0007 | p<0.05 |
|  |  |  |  | 4 vs. 0 | 0.0008 | p<0.05 |
|  |  |  |  | 5 vs. 0 | 0.4692 | ns |
|  |  |  |  | 6 vs. 0 | 0.1103 | ns |
|  |  |  |  | 7 vs. 0 | 0.3153 | ns |
|  |  |  |  | 8 vs. 0 | 0.9109 | ns |
| 17 | ANOVA | 0.8595 | <.0001 | 1 vs. 0 | 0.0005 | p<0.05 |
|  |  |  |  | 2 vs. 0 | 0.0003 | p<0.05 |
|  |  |  |  | 3 vs. 0 | 0.0009 | p<0.05 |
|  |  |  |  | 4 vs. 0 | 0.0007 | p<0.05 |
|  |  |  |  | 5 vs. 0 | 0.1922 | ns |
|  |  |  |  | 6 vs. 0 | 0.0755 | ns |
|  |  |  |  | 7 vs. 0 | 0.1135 | ns |
|  |  |  |  | 8 vs. 0 | 0.5913 | ns |
| 18 | ANOVA | 0.9191 | <.0001 | 1 vs. 0 | 0.0007 | p<0.05 |
|  |  |  |  | 2 vs. 0 | 0.0002 | p<0.05 |
|  |  |  |  | 3 vs. 0 | 0.0018 | p<0.05 |
|  |  |  |  | 4 vs. 0 | 0.0014 | p<0.05 |
|  |  |  |  | 5 vs. 0 | 0.2436 | ns |
|  |  |  |  | 6 vs. 0 | 0.1485 | ns |

| **Day** | **Test** | **Normality p** | **Main p** | **Comparison** | **Pairwise p** | **Significance** |
| --- | --- | --- | --- | --- | --- | --- |
|  |  |  |  | 7 vs. 0 | 0.2350 | ns |
|  |  |  |  | 8 vs. 0 | 0.8601 | ns |
| 19 | ANOVA | 0.2933 | <.0001 | 1 vs. 0 | <.0001 | p<0.05 |
|  |  |  |  | 2 vs. 0 | <.0001 | p<0.05 |
|  |  |  |  | 3 vs. 0 | <.0001 | p<0.05 |
|  |  |  |  | 4 vs. 0 | <.0001 | p<0.05 |
|  |  |  |  | 5 vs. 0 | 0.0709 | ns |
|  |  |  |  | 6 vs. 0 | 0.0145 | p<0.05 |
|  |  |  |  | 7 vs. 0 | 0.0425 | p<0.05 |
|  |  |  |  | 8 vs. 0 | 0.1694 | ns |
| 20 | ANOVA | 0.2090 | <.0001 | 1 vs. 0 | <.0001 | p<0.05 |
|  |  |  |  | 2 vs. 0 | <.0001 | p<0.05 |
|  |  |  |  | 3 vs. 0 | 0.0002 | p<0.05 |
|  |  |  |  | 4 vs. 0 | <.0001 | p<0.05 |
|  |  |  |  | 5 vs. 0 | 0.0314 | p<0.05 |
|  |  |  |  | 6 vs. 0 | 0.0109 | p<0.05 |
|  |  |  |  | 7 vs. 0 | 0.0505 | ns |
|  |  |  |  | 8 vs. 0 | 0.3473 | ns |
| 21 | ANOVA | 0.4133 | <.0001 | 1 vs. 0 | <.0001 | p<0.05 |
|  |  |  |  | 2 vs. 0 | <.0001 | p<0.05 |
|  |  |  |  | 3 vs. 0 | 0.0002 | p<0.05 |
|  |  |  |  | 4 vs. 0 | <.0001 | p<0.05 |
|  |  |  |  | 5 vs. 0 | 0.0446 | p<0.05 |
|  |  |  |  | 6 vs. 0 | 0.0277 | p<0.05 |
|  |  |  |  | 7 vs. 0 | 0.0825 | ns |
|  |  |  |  | 8 vs. 0 | 0.4658 | ns |

| **Day** | **Test** | **Normality p** | **Main p** | **Comparison** | **Pairwise p** | **Significance** |
| --- | --- | --- | --- | --- | --- | --- |
| 22 | ANOVA | 0.6050 | 0.0002 | 1 vs. 0 | 0.0004 | p<0.05 |
|  |  |  |  | 2 vs. 0 | <.0001 | p<0.05 |
|  |  |  |  | 3 vs. 0 | 0.0004 | p<0.05 |
|  |  |  |  | 4 vs. 0 | <.0001 | p<0.05 |
|  |  |  |  | 5 vs. 0 | 0.0243 | p<0.05 |
|  |  |  |  | 6 vs. 0 | 0.0103 | p<0.05 |
|  |  |  |  | 7 vs. 0 | 0.0280 | p<0.05 |
|  |  |  |  | 8 vs. 0 | 0.1560 | ns |
| 23 | ANOVA | 0.6294 | <.0001 | 1 vs. 0 | 0.0008 | p<0.05 |
|  |  |  |  | 2 vs. 0 | <.0001 | p<0.05 |
|  |  |  |  | 3 vs. 0 | 0.0007 | p<0.05 |
|  |  |  |  | 4 vs. 0 | 0.0002 | p<0.05 |
|  |  |  |  | 5 vs. 0 | 0.0502 | ns |
|  |  |  |  | 6 vs. 0 | 0.0229 | p<0.05 |
|  |  |  |  | 7 vs. 0 | 0.0596 | ns |
|  |  |  |  | 8 vs. 0 | 0.2324 | ns |
| 24 | ANOVA | 0.6168 | <.0001 | 1 vs. 0 | <.0001 | p<0.05 |
|  |  |  |  | 2 vs. 0 | <.0001 | p<0.05 |
|  |  |  |  | 3 vs. 0 | <.0001 | p<0.05 |
|  |  |  |  | 4 vs. 0 | <.0001 | p<0.05 |
|  |  |  |  | 5 vs. 0 | 0.0065 | p<0.05 |
|  |  |  |  | 6 vs. 0 | 0.0026 | p<0.05 |
|  |  |  |  | 7 vs. 0 | 0.0090 | p<0.05 |
|  |  |  |  | 8 vs. 0 | 0.0709 | ns |
| 25 | ANOVA | 0.4252 | 0.0002 | 1 vs. 0 | 0.0010 | p<0.05 |
|  |  |  |  | 2 vs. 0 | 0.0002 | p<0.05 |

| **Day** | **Test** | **Normality p** | **Main p** | **Comparison** | **Pairwise p** | **Significance** |
| --- | --- | --- | --- | --- | --- | --- |
|  |  |  |  | 3 vs. 0 | 0.0011 | p<0.05 |
|  |  |  |  | 4 vs. 0 | 0.0003 | p<0.05 |
|  |  |  |  | 5 vs. 0 | 0.0387 | p<0.05 |
|  |  |  |  | 6 vs. 0 | 0.0266 | p<0.05 |
|  |  |  |  | 7 vs. 0 | 0.0839 | ns |
|  |  |  |  | 8 vs. 0 | 0.3304 | ns |
| 26 | ANOVA | 0.3778 | 0.0011 | 1 vs. 0 | 0.0017 | p<0.05 |
|  |  |  |  | 2 vs. 0 | 0.0002 | p<0.05 |
|  |  |  |  | 3 vs. 0 | 0.0006 | p<0.05 |
|  |  |  |  | 4 vs. 0 | 0.0002 | p<0.05 |
|  |  |  |  | 5 vs. 0 | 0.0161 | p<0.05 |
|  |  |  |  | 6 vs. 0 | 0.0105 | p<0.05 |
|  |  |  |  | 7 vs. 0 | 0.0391 | p<0.05 |
|  |  |  |  | 8 vs. 0 | 0.1326 | ns |
| 27 | ANOVA | 0.1199 | 0.0017 | 1 vs. 0 | 0.0015 | p<0.05 |
|  |  |  |  | 2 vs. 0 | 0.0003 | p<0.05 |
|  |  |  |  | 3 vs. 0 | 0.0008 | p<0.05 |
|  |  |  |  | 4 vs. 0 | 0.0005 | p<0.05 |
|  |  |  |  | 5 vs. 0 | 0.0338 | p<0.05 |
|  |  |  |  | 6 vs. 0 | 0.0157 | p<0.05 |
|  |  |  |  | 7 vs. 0 | 0.0384 | p<0.05 |
|  |  |  |  | 8 vs. 0 | 0.1913 | ns |
| 28 | ANOVA | 0.7648 | 0.0028 | 1 vs. 0 | 0.0021 | p<0.05 |
|  |  |  |  | 2 vs. 0 | 0.0005 | p<0.05 |
|  |  |  |  | 3 vs. 0 | 0.0016 | p<0.05 |
|  |  |  |  | 4 vs. 0 | 0.0009 | p<0.05 |

| **Day** | **Test** | **Normality p** | **Main p** | **Comparison** | **Pairwise p** | **Significance** |
| --- | --- | --- | --- | --- | --- | --- |
|  |  |  |  | 5 vs. 0 | 0.0345 | p<0.05 |
|  |  |  |  | 6 vs. 0 | 0.0276 | p<0.05 |
|  |  |  |  | 7 vs. 0 | 0.0736 | ns |
|  |  |  |  | 8 vs. 0 | 0.2362 | ns |
| 29 | ANOVA | 0.2151 | 0.0071 | 1 vs. 0 | 0.0050 | p<0.05 |
|  |  |  |  | 2 vs. 0 | 0.0015 | p<0.05 |
|  |  |  |  | 3 vs. 0 | 0.0028 | p<0.05 |
|  |  |  |  | 4 vs. 0 | 0.0019 | p<0.05 |
|  |  |  |  | 5 vs. 0 | 0.0392 | p<0.05 |
|  |  |  |  | 6 vs. 0 | 0.0536 | ns |
|  |  |  |  | 7 vs. 0 | 0.1151 | ns |
|  |  |  |  | 8 vs. 0 | 0.3133 | ns |
| 30 | ANOVA | 0.3459 | 0.0076 | 1 vs. 0 | 0.0023 | p<0.05 |
|  |  |  |  | 2 vs. 0 | 0.0006 | p<0.05 |
|  |  |  |  | 3 vs. 0 | 0.0016 | p<0.05 |
|  |  |  |  | 4 vs. 0 | 0.0009 | p<0.05 |
|  |  |  |  | 5 vs. 0 | 0.0149 | p<0.05 |
|  |  |  |  | 6 vs. 0 | 0.0415 | p<0.05 |
|  |  |  |  | 7 vs. 0 | 0.0472 | p<0.05 |
|  |  |  |  | 8 vs. 0 | 0.1548 | ns |
| 31 | ANOVA | 0.6910 | 0.0098 | 1 vs. 0 | 0.0043 | p<0.05 |
|  |  |  |  | 2 vs. 0 | 0.0012 | p<0.05 |
|  |  |  |  | 3 vs. 0 | 0.0040 | p<0.05 |
|  |  |  |  | 4 vs. 0 | 0.0028 | p<0.05 |
|  |  |  |  | 5 vs. 0 | 0.0383 | p<0.05 |
|  |  |  |  | 6 vs. 0 | 0.1053 | ns |

| **Day** | **Test** | **Normality p** | **Main p** | **Comparison** | **Pairwise p** | **Significance** |
| --- | --- | --- | --- | --- | --- | --- |
|  |  |  |  | 7 vs. 0 | 0.1048 | ns |
|  |  |  |  | 8 vs. 0 | 0.2423 | ns |
| 32 | ANOVA | 0.5334 | 0.0354 | 1 vs. 0 | 0.0182 | p<0.05 |
|  |  |  |  | 2 vs. 0 | 0.0036 | p<0.05 |
|  |  |  |  | 3 vs. 0 | 0.0112 | p<0.05 |
|  |  |  |  | 4 vs. 0 | 0.0057 | p<0.05 |
|  |  |  |  | 5 vs. 0 | 0.0734 | ns |
|  |  |  |  | 6 vs. 0 | 0.1430 | ns |
|  |  |  |  | 7 vs. 0 | 0.1687 | ns |
|  |  |  |  | 8 vs. 0 | 0.2590 | ns |
| 33 | ANOVA | 0.4240 | 0.0560 | 1 vs. 0 | 0.0112 | p<0.05 |
|  |  |  |  | 2 vs. 0 | 0.0035 | p<0.05 |
|  |  |  |  | 3 vs. 0 | 0.0106 | p<0.05 |
|  |  |  |  | 4 vs. 0 | 0.0033 | p<0.05 |
|  |  |  |  | 5 vs. 0 | 0.0269 | p<0.05 |
|  |  |  |  | 6 vs. 0 | 0.0845 | ns |
|  |  |  |  | 7 vs. 0 | 0.0916 | ns |
|  |  |  |  | 8 vs. 0 | 0.2123 | ns |
| 34 | ANOVA | 0.3772 | 0.1261 | 1 vs. 0 | 0.0320 | p<0.05 |
|  |  |  |  | 2 vs. 0 | 0.0132 | p<0.05 |
|  |  |  |  | 3 vs. 0 | 0.0346 | p<0.05 |
|  |  |  |  | 4 vs. 0 | 0.0106 | p<0.05 |
|  |  |  |  | 5 vs. 0 | 0.0770 | ns |
|  |  |  |  | 6 vs. 0 | 0.1821 | ns |
|  |  |  |  | 7 vs. 0 | 0.2217 | ns |
|  |  |  |  | 8 vs. 0 | 0.2881 | ns |

| **Day** | **Test** | **Normality p** | **Main p** | **Comparison** | **Pairwise p** | **Significance** |
| --- | --- | --- | --- | --- | --- | --- |
| 35 | ANOVA | 0.5830 | 0.0762 | 1 vs. 0 | 0.0149 | p<0.05 |
|  |  |  |  | 2 vs. 0 | 0.0043 | p<0.05 |
|  |  |  |  | 3 vs. 0 | 0.0123 | p<0.05 |
|  |  |  |  | 4 vs. 0 | 0.0047 | p<0.05 |
|  |  |  |  | 5 vs. 0 | 0.0332 | p<0.05 |
|  |  |  |  | 6 vs. 0 | 0.1017 | ns |
|  |  |  |  | 7 vs. 0 | 0.0951 | ns |
|  |  |  |  | 8 vs. 0 | 0.2283 | ns |
| 36 | ANOVA | 0.4360 | 0.0809 | 1 vs. 0 | 0.0339 | p<0.05 |
|  |  |  |  | 2 vs. 0 | 0.0048 | p<0.05 |
|  |  |  |  | 3 vs. 0 | 0.0111 | p<0.05 |
|  |  |  |  | 4 vs. 0 | 0.0064 | p<0.05 |
|  |  |  |  | 5 vs. 0 | 0.0509 | ns |
|  |  |  |  | 6 vs. 0 | 0.0829 | ns |
|  |  |  |  | 7 vs. 0 | 0.1142 | ns |
|  |  |  |  | 8 vs. 0 | 0.2997 | ns |
| 37 | ANOVA | 0.5559 | 0.1246 | 1 vs. 0 | 0.0396 | p<0.05 |
|  |  |  |  | 2 vs. 0 | 0.0095 | p<0.05 |
|  |  |  |  | 3 vs. 0 | 0.0205 | p<0.05 |
|  |  |  |  | 4 vs. 0 | 0.0084 | p<0.05 |
|  |  |  |  | 5 vs. 0 | 0.0580 | ns |
|  |  |  |  | 6 vs. 0 | 0.1463 | ns |
|  |  |  |  | 7 vs. 0 | 0.1449 | ns |
|  |  |  |  | 8 vs. 0 | 0.3010 | ns |
| 38 | ANOVA | 0.4875 | 0.1833 | 1 vs. 0 | 0.1671 | ns |
|  |  |  |  | 2 vs. 0 | 0.0505 | ns |

| **Day** | **Test** | **Normality p** | **Main p** | **Comparison** | **Pairwise p** | **Significance** |
| --- | --- | --- | --- | --- | --- | --- |
|  |  |  |  | 3 vs. 0 | 0.0761 | ns |
|  |  |  |  | 4 vs. 0 | 0.0521 | ns |
|  |  |  |  | 5 vs. 0 | 0.1874 | ns |
|  |  |  |  | 6 vs. 0 | 0.2840 | ns |
|  |  |  |  | 7 vs. 0 | 0.3858 | ns |
|  |  |  |  | 8 vs. 0 | 0.9645 | ns |
| 39 | ANOVA | 0.4045 | 0.4513 | 1 vs. 0 | 0.1732 | ns |
|  |  |  |  | 2 vs. 0 | 0.0474 | p<0.05 |
|  |  |  |  | 3 vs. 0 | 0.0898 | ns |
|  |  |  |  | 4 vs. 0 | 0.0390 | p<0.05 |
|  |  |  |  | 5 vs. 0 | 0.0993 | ns |
|  |  |  |  | 6 vs. 0 | 0.1424 | ns |
|  |  |  |  | 7 vs. 0 | 0.1581 | ns |
|  |  |  |  | 8 vs. 0 | 0.6453 | ns |
| 40 | ANOVA | 0.3661 | 0.5370 | 1 vs. 0 | 0.1775 | ns |
|  |  |  |  | 2 vs. 0 | 0.0419 | p<0.05 |
|  |  |  |  | 3 vs. 0 | 0.0988 | ns |
|  |  |  |  | 4 vs. 0 | 0.0476 | p<0.05 |
|  |  |  |  | 5 vs. 0 | 0.0621 | ns |
|  |  |  |  | 6 vs. 0 | 0.1650 | ns |
|  |  |  |  | 7 vs. 0 | 0.1781 | ns |
|  |  |  |  | 8 vs. 0 | 0.4673 | ns |
| 41 | ANOVA | 0.3333 | 0.4815 | 1 vs. 0 | 0.1356 | ns |
|  |  |  |  | 2 vs. 0 | 0.0523 | ns |
|  |  |  |  | 3 vs. 0 | 0.1738 | ns |
|  |  |  |  | 4 vs. 0 | 0.0673 | ns |

| **Day** | **Test** | **Normality p** | **Main p** | **Comparison** | **Pairwise p** | **Significance** |
| --- | --- | --- | --- | --- | --- | --- |
|  |  |  |  | 5 vs. 0 | 0.1268 | ns |
|  |  |  |  | 6 vs. 0 | 0.2598 | ns |
|  |  |  |  | 7 vs. 0 | 0.2687 | ns |
|  |  |  |  | 8 vs. 0 | 0.7170 | ns |
| 42 | ANOVA | 0.1796 | 0.4487 | 1 vs. 0 | 0.1150 | ns |
|  |  |  |  | 2 vs. 0 | 0.0452 | p<0.05 |
|  |  |  |  | 3 vs. 0 | 0.1195 | ns |
|  |  |  |  | 4 vs. 0 | 0.0488 | p<0.05 |
|  |  |  |  | 5 vs. 0 | 0.0831 | ns |
|  |  |  |  | 6 vs. 0 | 0.1839 | ns |
|  |  |  |  | 7 vs. 0 | 0.2002 | ns |
|  |  |  |  | 8 vs. 0 | 0.6631 | ns |
| 43 | ANOVA | 0.3659 | 0.3904 | 1 vs. 0 | 0.1547 | ns |
|  |  |  |  | 2 vs. 0 | 0.0401 | p<0.05 |
|  |  |  |  | 3 vs. 0 | 0.1193 | ns |
|  |  |  |  | 4 vs. 0 | 0.0480 | p<0.05 |
|  |  |  |  | 5 vs. 0 | 0.0942 | ns |
|  |  |  |  | 6 vs. 0 | 0.2452 | ns |
|  |  |  |  | 7 vs. 0 | 0.2160 | ns |
|  |  |  |  | 8 vs. 0 | 0.7037 | ns |
| 44 | ANOVA | 0.1688 | 0.4313 | 1 vs. 0 | 0.2801 | ns |
|  |  |  |  | 2 vs. 0 | 0.0724 | ns |
|  |  |  |  | 3 vs. 0 | 0.2511 | ns |
|  |  |  |  | 4 vs. 0 | 0.0969 | ns |
|  |  |  |  | 5 vs. 0 | 0.1787 | ns |
|  |  |  |  | 6 vs. 0 | 0.4121 | ns |

| **Day** | **Test** | **Normality p** | **Main p** | **Comparison** | **Pairwise p** | **Significance** |
| --- | --- | --- | --- | --- | --- | --- |
|  |  |  |  | 7 vs. 0 | 0.3067 | ns |
|  |  |  |  | 8 vs. 0 | 0.9952 | ns |
| 45 | ANOVA | 0.1713 | 0.0018 | 1 vs. 0 | 0.0012 | p<0.05 |
|  |  |  |  | 2 vs. 0 | 0.0001 | p<0.05 |
|  |  |  |  | 3 vs. 0 | 0.0006 | p<0.05 |
|  |  |  |  | 4 vs. 0 | 0.0001 | p<0.05 |

| **Day** | **Test** | **Normality p** | **Main p** | **Comparison** | **Pairwise p** | **Significance** |
| --- | --- | --- | --- | --- | --- | --- |
| 0 | ANOVA | 0.0138 | 0.9992 | 1 vs. 0 | 0.5839 | ns |
|  |  |  |  | 2 vs. 0 | 0.7394 | ns |
|  |  |  |  | 3 vs. 0 | 0.9113 | ns |
|  |  |  |  | 4 vs. 0 | 0.8677 | ns |
|  |  |  |  | 5 vs. 0 | 0.8478 | ns |
|  |  |  |  | 6 vs. 0 | 0.8655 | ns |
|  |  |  |  | 7 vs. 0 | 0.8854 | ns |
|  |  |  |  | 8 vs. 0 | 0.9825 | ns |
| 1 | ANOVA | 0.0110 | 0.9985 | 1 vs. 0 | 0.5548 | ns |
|  |  |  |  | 2 vs. 0 | 0.6711 | ns |
|  |  |  |  | 3 vs. 0 | 0.9428 | ns |
|  |  |  |  | 4 vs. 0 | 0.8432 | ns |
|  |  |  |  | 5 vs. 0 | 0.9566 | ns |
|  |  |  |  | 6 vs. 0 | 0.9952 | ns |
|  |  |  |  | 7 vs. 0 | 0.9566 | ns |
|  |  |  |  | 8 vs. 0 | 0.9855 | ns |
| 2 | ANOVA | 0.0066 | 0.9986 | 1 vs. 0 | 0.5396 | ns |
|  |  |  |  | 2 vs. 0 | 0.7057 | ns |
|  |  |  |  | 3 vs. 0 | 0.9238 | ns |
|  |  |  |  | 4 vs. 0 | 0.8595 | ns |
|  |  |  |  | 5 vs. 0 | 0.9498 | ns |
|  |  |  |  | 6 vs. 0 | 0.9498 | ns |
|  |  |  |  | 7 vs. 0 | 0.8749 | ns |
|  |  |  |  | 8 vs. 0 | 0.9874 | ns |
| 3 | ANOVA | 0.0068 | 0.9968 | 1 vs. 0 | 0.5057 | ns |
|  |  |  |  | 2 vs. 0 | 0.6384 | ns |

| **Day** | **Test** | **Normality p** | **Main p** | **Comparison** | **Pairwise p** | **Significance** |
| --- | --- | --- | --- | --- | --- | --- |
|  |  |  |  | 3 vs. 0 | 0.9487 | ns |
|  |  |  |  | 4 vs. 0 | 0.8272 | ns |
|  |  |  |  | 5 vs. 0 | 0.9952 | ns |
|  |  |  |  | 6 vs. 0 | 0.9604 | ns |
|  |  |  |  | 7 vs. 0 | 0.9415 | ns |
|  |  |  |  | 8 vs. 0 | 0.9857 | ns |
| 4 | ANOVA | 0.0036 | 0.9936 | 1 vs. 0 | 0.4690 | ns |
|  |  |  |  | 2 vs. 0 | 0.6124 | ns |
|  |  |  |  | 3 vs. 0 | 0.9719 | ns |
|  |  |  |  | 4 vs. 0 | 0.8536 | ns |
|  |  |  |  | 5 vs. 0 | 0.9449 | ns |
|  |  |  |  | 6 vs. 0 | 0.9764 | ns |
|  |  |  |  | 7 vs. 0 | 0.9858 | ns |
|  |  |  |  | 8 vs. 0 | 0.8327 | ns |
| 5 | ANOVA | 0.0039 | 0.9730 | 1 vs. 0 | 0.3326 | ns |
|  |  |  |  | 2 vs. 0 | 0.4130 | ns |
|  |  |  |  | 3 vs. 0 | 0.7818 | ns |
|  |  |  |  | 4 vs. 0 | 0.9906 | ns |
|  |  |  |  | 5 vs. 0 | 0.9477 | ns |
|  |  |  |  | 6 vs. 0 | 0.9794 | ns |
|  |  |  |  | 7 vs. 0 | 0.9952 | ns |
|  |  |  |  | 8 vs. 0 | 0.9445 | ns |
| 6 | ANOVA | 0.0070 | 0.9463 | 1 vs. 0 | 0.2696 | ns |
|  |  |  |  | 2 vs. 0 | 0.3415 | ns |
|  |  |  |  | 3 vs. 0 | 0.6230 | ns |
|  |  |  |  | 4 vs. 0 | 0.8872 | ns |

| **Day** | **Test** | **Normality p** | **Main p** | **Comparison** | **Pairwise p** | **Significance** |
| --- | --- | --- | --- | --- | --- | --- |
|  |  |  |  | 5 vs. 0 | 0.9141 | ns |
|  |  |  |  | 6 vs. 0 | 0.9522 | ns |
|  |  |  |  | 7 vs. 0 | 0.9777 | ns |
|  |  |  |  | 8 vs. 0 | 0.9968 | ns |
| 7 | ANOVA | 0.0045 | 0.9415 | 1 vs. 0 | 0.2725 | ns |
|  |  |  |  | 2 vs. 0 | 0.3217 | ns |
|  |  |  |  | 3 vs. 0 | 0.6104 | ns |
|  |  |  |  | 4 vs. 0 | 0.8159 | ns |
|  |  |  |  | 5 vs. 0 | 0.9717 | ns |
|  |  |  |  | 6 vs. 0 | 0.9780 | ns |
|  |  |  |  | 7 vs. 0 | 0.9717 | ns |
|  |  |  |  | 8 vs. 0 | 0.9780 | ns |
| 8 | ANOVA | 0.0057 | 0.9072 | 1 vs. 0 | 0.2681 | ns |
|  |  |  |  | 2 vs. 0 | 0.3005 | ns |
|  |  |  |  | 3 vs. 0 | 0.6854 | ns |
|  |  |  |  | 4 vs. 0 | 0.8842 | ns |
|  |  |  |  | 5 vs. 0 | 0.9747 | ns |
|  |  |  |  | 6 vs. 0 | 0.8738 | ns |
|  |  |  |  | 7 vs. 0 | 0.9810 | ns |
|  |  |  |  | 8 vs. 0 | 0.9101 | ns |
| 9 | ANOVA | 0.0175 | 0.9235 | 1 vs. 0 | 0.3226 | ns |
|  |  |  |  | 2 vs. 0 | 0.3999 | ns |
|  |  |  |  | 3 vs. 0 | 0.8804 | ns |
|  |  |  |  | 4 vs. 0 | 0.9827 | ns |
|  |  |  |  | 5 vs. 0 | 0.9062 | ns |
|  |  |  |  | 6 vs. 0 | 0.7344 | ns |

| **Day** | **Test** | **Normality p** | **Main p** | **Comparison** | **Pairwise p** | **Significance** |
| --- | --- | --- | --- | --- | --- | --- |
|  |  |  |  | 7 vs. 0 | 0.8760 | ns |
|  |  |  |  | 8 vs. 0 | 0.8845 | ns |
| -1 | ANOVA | 0.0136 | 0.9996 | 1 vs. 0 | 0.6593 | ns |
|  |  |  |  | 2 vs. 0 | 0.7569 | ns |
|  |  |  |  | 3 vs. 0 | 0.8824 | ns |
|  |  |  |  | 4 vs. 0 | 0.8362 | ns |
|  |  |  |  | 5 vs. 0 | 0.8926 | ns |
|  |  |  |  | 6 vs. 0 | 0.9052 | ns |
|  |  |  |  | 7 vs. 0 | 0.9052 | ns |
|  |  |  |  | 8 vs. 0 | 0.9620 | ns |
| -2 | ANOVA | 0.0090 | 0.9995 | 1 vs. 0 | 0.5761 | ns |
|  |  |  |  | 2 vs. 0 | 0.7629 | ns |
|  |  |  |  | 3 vs. 0 | 0.9191 | ns |
|  |  |  |  | 4 vs. 0 | 0.9151 | ns |
|  |  |  |  | 5 vs. 0 | 0.8355 | ns |
|  |  |  |  | 6 vs. 0 | 0.9033 | ns |
|  |  |  |  | 7 vs. 0 | 0.8847 | ns |
|  |  |  |  | 8 vs. 0 | 0.9906 | ns |
| -3 | ANOVA | 0.0129 | 0.9984 | 1 vs. 0 | 0.5089 | ns |
|  |  |  |  | 2 vs. 0 | 0.7180 | ns |
|  |  |  |  | 3 vs. 0 | 0.9385 | ns |
|  |  |  |  | 4 vs. 0 | 0.8805 | ns |
|  |  |  |  | 5 vs. 0 | 0.8520 | ns |
|  |  |  |  | 6 vs. 0 | 0.8769 | ns |
|  |  |  |  | 7 vs. 0 | 0.8582 | ns |
|  |  |  |  | 8 vs. 0 | 0.9399 | ns |

| **Day** | **Test** | **Normality p** | **Main p** | **Comparison** | **Pairwise p** | **Significance** |
| --- | --- | --- | --- | --- | --- | --- |
| -4 | ANOVA | 0.0147 | 0.9984 | 1 vs. 0 | 0.5309 | ns |
|  |  |  |  | 2 vs. 0 | 0.7303 | ns |
|  |  |  |  | 3 vs. 0 | 0.8819 | ns |
|  |  |  |  | 4 vs. 0 | 0.8633 | ns |
|  |  |  |  | 5 vs. 0 | 0.8906 | ns |
|  |  |  |  | 6 vs. 0 | 0.8654 | ns |
|  |  |  |  | 7 vs. 0 | 0.9222 | ns |
|  |  |  |  | 8 vs. 0 | 0.9825 | ns |
| -5 | ANOVA | 0.0176 | 0.9995 | 1 vs. 0 | 0.6242 | ns |
|  |  |  |  | 2 vs. 0 | 0.7564 | ns |
|  |  |  |  | 3 vs. 0 | 0.9974 | ns |
|  |  |  |  | 4 vs. 0 | 0.8525 | ns |
|  |  |  |  | 5 vs. 0 | 0.8246 | ns |
|  |  |  |  | 6 vs. 0 | 0.8184 | ns |
|  |  |  |  | 7 vs. 0 | 0.8751 | ns |
|  |  |  |  | 8 vs. 0 | 0.9968 | ns |
| -6 | ANOVA | 0.0176 | 0.9986 | 1 vs. 0 | 0.6147 | ns |
|  |  |  |  | 2 vs. 0 | 0.7139 | ns |
|  |  |  |  | 3 vs. 0 | 0.8506 | ns |
|  |  |  |  | 4 vs. 0 | 0.8588 | ns |
|  |  |  |  | 5 vs. 0 | 0.7929 | ns |
|  |  |  |  | 6 vs. 0 | 0.8239 | ns |
|  |  |  |  | 7 vs. 0 | 0.8679 | ns |
|  |  |  |  | 8 vs. 0 | 0.9888 | ns |
| 10 | ANOVA | 0.0068 | 0.9488 | 1 vs. 0 | 0.3421 | ns |
|  |  |  |  | 2 vs. 0 | 0.3777 | ns |

| **Day** | **Test** | **Normality p** | **Main p** | **Comparison** | **Pairwise p** | **Significance** |
| --- | --- | --- | --- | --- | --- | --- |
|  |  |  |  | 3 vs. 0 | 0.7708 | ns |
|  |  |  |  | 4 vs. 0 | 0.9023 | ns |
|  |  |  |  | 5 vs. 0 | 0.9743 | ns |
|  |  |  |  | 6 vs. 0 | 0.9358 | ns |
|  |  |  |  | 7 vs. 0 | 0.9241 | ns |
|  |  |  |  | 8 vs. 0 | 0.9818 | ns |
| 11 | ANOVA | 0.0094 | 0.9305 | 1 vs. 0 | 0.5227 | ns |
|  |  |  |  | 2 vs. 0 | 0.5661 | ns |
|  |  |  |  | 3 vs. 0 | 0.9874 | ns |
|  |  |  |  | 4 vs. 0 | 0.7995 | ns |
|  |  |  |  | 5 vs. 0 | 0.6936 | ns |
|  |  |  |  | 6 vs. 0 | 0.8440 | ns |
|  |  |  |  | 7 vs. 0 | 0.6546 | ns |
|  |  |  |  | 8 vs. 0 | 0.7697 | ns |
| 12 | ANOVA | 0.0189 | 0.9371 | 1 vs. 0 | 0.5904 | ns |
|  |  |  |  | 2 vs. 0 | 0.6793 | ns |
|  |  |  |  | 3 vs. 0 | 0.9938 | ns |
|  |  |  |  | 4 vs. 0 | 0.8384 | ns |
|  |  |  |  | 5 vs. 0 | 0.7274 | ns |
|  |  |  |  | 6 vs. 0 | 0.7696 | ns |
|  |  |  |  | 7 vs. 0 | 0.6117 | ns |
|  |  |  |  | 8 vs. 0 | 0.6857 | ns |
| 13 | ANOVA | 0.0168 | 0.9623 | 1 vs. 0 | 0.5367 | ns |
|  |  |  |  | 2 vs. 0 | 0.6164 | ns |
|  |  |  |  | 3 vs. 0 | 0.9256 | ns |
|  |  |  |  | 4 vs. 0 | 0.9421 | ns |

| **Day** | **Test** | **Normality p** | **Main p** | **Comparison** | **Pairwise p** | **Significance** |
| --- | --- | --- | --- | --- | --- | --- |
|  |  |  |  | 5 vs. 0 | 0.8042 | ns |
|  |  |  |  | 6 vs. 0 | 0.9256 | ns |
|  |  |  |  | 7 vs. 0 | 0.7038 | ns |
|  |  |  |  | 8 vs. 0 | 0.9046 | ns |
| 14 | ANOVA | 0.0363 | 0.9222 | 1 vs. 0 | 0.7900 | ns |
|  |  |  |  | 2 vs. 0 | 0.9270 | ns |
|  |  |  |  | 3 vs. 0 | 0.7141 | ns |
|  |  |  |  | 4 vs. 0 | 0.6460 | ns |
|  |  |  |  | 5 vs. 0 | 0.5355 | ns |
|  |  |  |  | 6 vs. 0 | 0.6403 | ns |
|  |  |  |  | 7 vs. 0 | 0.4774 | ns |
|  |  |  |  | 8 vs. 0 | 0.6012 | ns |
| 15 | ANOVA | 0.0309 | 0.9425 | 1 vs. 0 | 0.7720 | ns |
|  |  |  |  | 2 vs. 0 | 0.8537 | ns |
|  |  |  |  | 3 vs. 0 | 0.7843 | ns |
|  |  |  |  | 4 vs. 0 | 0.7037 | ns |
|  |  |  |  | 5 vs. 0 | 0.5912 | ns |
|  |  |  |  | 6 vs. 0 | 0.7500 | ns |
|  |  |  |  | 7 vs. 0 | 0.5497 | ns |
|  |  |  |  | 8 vs. 0 | 0.6406 | ns |
| 16 | ANOVA | 0.0384 | 0.9559 | 1 vs. 0 | 0.8470 | ns |
|  |  |  |  | 2 vs. 0 | 0.8755 | ns |
|  |  |  |  | 3 vs. 0 | 0.7812 | ns |
|  |  |  |  | 4 vs. 0 | 0.7116 | ns |
|  |  |  |  | 5 vs. 0 | 0.5418 | ns |
|  |  |  |  | 6 vs. 0 | 0.7588 | ns |

| **Day** | **Test** | **Normality p** | **Main p** | **Comparison** | **Pairwise p** | **Significance** |
| --- | --- | --- | --- | --- | --- | --- |
|  |  |  |  | 7 vs. 0 | 0.5654 | ns |
|  |  |  |  | 8 vs. 0 | 0.6101 | ns |
| 17 | ANOVA | 0.0230 | 0.9747 | 1 vs. 0 | 0.7431 | ns |
|  |  |  |  | 2 vs. 0 | 0.8609 | ns |
|  |  |  |  | 3 vs. 0 | 0.8418 | ns |
|  |  |  |  | 4 vs. 0 | 0.7936 | ns |
|  |  |  |  | 5 vs. 0 | 0.6358 | ns |
|  |  |  |  | 6 vs. 0 | 0.8270 | ns |
|  |  |  |  | 7 vs. 0 | 0.6729 | ns |
|  |  |  |  | 8 vs. 0 | 0.6785 | ns |
| 18 | ANOVA | 0.0282 | 0.9622 | 1 vs. 0 | 0.7653 | ns |
|  |  |  |  | 2 vs. 0 | 0.8722 | ns |
|  |  |  |  | 3 vs. 0 | 0.7882 | ns |
|  |  |  |  | 4 vs. 0 | 0.7471 | ns |
|  |  |  |  | 5 vs. 0 | 0.6120 | ns |
|  |  |  |  | 6 vs. 0 | 0.7761 | ns |
|  |  |  |  | 7 vs. 0 | 0.6085 | ns |
|  |  |  |  | 8 vs. 0 | 0.6244 | ns |
| 19 | ANOVA | 0.0393 | 0.9731 | 1 vs. 0 | 0.6949 | ns |
|  |  |  |  | 2 vs. 0 | 0.8012 | ns |
|  |  |  |  | 3 vs. 0 | 0.8797 | ns |
|  |  |  |  | 4 vs. 0 | 0.8175 | ns |
|  |  |  |  | 5 vs. 0 | 0.6812 | ns |
|  |  |  |  | 6 vs. 0 | 0.8901 | ns |
|  |  |  |  | 7 vs. 0 | 0.7000 | ns |
|  |  |  |  | 8 vs. 0 | 0.7757 | ns |

| **Day** | **Test** | **Normality p** | **Main p** | **Comparison** | **Pairwise p** | **Significance** |
| --- | --- | --- | --- | --- | --- | --- |
| 20 | ANOVA | 0.0408 | 0.9726 | 1 vs. 0 | 0.7036 | ns |
|  |  |  |  | 2 vs. 0 | 0.7840 | ns |
|  |  |  |  | 3 vs. 0 | 0.8533 | ns |
|  |  |  |  | 4 vs. 0 | 0.8535 | ns |
|  |  |  |  | 5 vs. 0 | 0.7224 | ns |
|  |  |  |  | 6 vs. 0 | 0.9085 | ns |
|  |  |  |  | 7 vs. 0 | 0.6885 | ns |
|  |  |  |  | 8 vs. 0 | 0.7097 | ns |
| 21 | ANOVA | 0.0246 | 0.9728 | 1 vs. 0 | 0.7339 | ns |
|  |  |  |  | 2 vs. 0 | 0.8339 | ns |
|  |  |  |  | 3 vs. 0 | 0.8306 | ns |
|  |  |  |  | 4 vs. 0 | 0.8077 | ns |
|  |  |  |  | 5 vs. 0 | 0.6901 | ns |
|  |  |  |  | 6 vs. 0 | 0.8451 | ns |
|  |  |  |  | 7 vs. 0 | 0.6492 | ns |
|  |  |  |  | 8 vs. 0 | 0.6740 | ns |
| 22 | ANOVA | 0.0280 | 0.9938 | 1 vs. 0 | 0.7524 | ns |
|  |  |  |  | 2 vs. 0 | 0.8234 | ns |
|  |  |  |  | 3 vs. 0 | 0.8658 | ns |
|  |  |  |  | 4 vs. 0 | 0.8793 | ns |
|  |  |  |  | 5 vs. 0 | 0.7726 | ns |
|  |  |  |  | 6 vs. 0 | 0.9485 | ns |
|  |  |  |  | 7 vs. 0 | 0.7610 | ns |
|  |  |  |  | 8 vs. 0 | 0.8100 | ns |
| 23 | ANOVA | 0.0315 | 0.9878 | 1 vs. 0 | 0.7721 | ns |
|  |  |  |  | 2 vs. 0 | 0.8059 | ns |

| **Day** | **Test** | **Normality p** | **Main p** | **Comparison** | **Pairwise p** | **Significance** |
| --- | --- | --- | --- | --- | --- | --- |
|  |  |  |  | 3 vs. 0 | 0.8366 | ns |
|  |  |  |  | 4 vs. 0 | 0.8481 | ns |
|  |  |  |  | 5 vs. 0 | 0.7240 | ns |
|  |  |  |  | 6 vs. 0 | 0.8963 | ns |
|  |  |  |  | 7 vs. 0 | 0.7125 | ns |
|  |  |  |  | 8 vs. 0 | 0.7730 | ns |
| 24 | ANOVA | 0.0285 | 0.9950 | 1 vs. 0 | 0.7060 | ns |
|  |  |  |  | 2 vs. 0 | 0.7512 | ns |
|  |  |  |  | 3 vs. 0 | 0.9689 | ns |
|  |  |  |  | 4 vs. 0 | 0.9528 | ns |
|  |  |  |  | 5 vs. 0 | 0.8483 | ns |
|  |  |  |  | 6 vs. 0 | 0.9830 | ns |
|  |  |  |  | 7 vs. 0 | 0.8286 | ns |
|  |  |  |  | 8 vs. 0 | 0.8712 | ns |
| 25 | ANOVA | 0.0205 | 0.9868 | 1 vs. 0 | 0.7044 | ns |
|  |  |  |  | 2 vs. 0 | 0.7498 | ns |
|  |  |  |  | 3 vs. 0 | 0.8970 | ns |
|  |  |  |  | 4 vs. 0 | 0.9159 | ns |
|  |  |  |  | 5 vs. 0 | 0.8119 | ns |
|  |  |  |  | 6 vs. 0 | 0.9657 | ns |
|  |  |  |  | 7 vs. 0 | 0.7536 | ns |
|  |  |  |  | 8 vs. 0 | 0.7893 | ns |
| 26 | ANOVA | 0.0174 | 0.9952 | 1 vs. 0 | 0.7290 | ns |
|  |  |  |  | 2 vs. 0 | 0.7439 | ns |
|  |  |  |  | 3 vs. 0 | 0.9401 | ns |
|  |  |  |  | 4 vs. 0 | 0.9521 | ns |

| **Day** | **Test** | **Normality p** | **Main p** | **Comparison** | **Pairwise p** | **Significance** |
| --- | --- | --- | --- | --- | --- | --- |
|  |  |  |  | 5 vs. 0 | 0.8702 | ns |
|  |  |  |  | 6 vs. 0 | 0.9769 | ns |
|  |  |  |  | 7 vs. 0 | 0.8064 | ns |
|  |  |  |  | 8 vs. 0 | 0.8766 | ns |
| 27 | ANOVA | 0.0217 | 0.9944 | 1 vs. 0 | 0.7703 | ns |
|  |  |  |  | 2 vs. 0 | 0.8123 | ns |
|  |  |  |  | 3 vs. 0 | 0.8635 | ns |
|  |  |  |  | 4 vs. 0 | 0.8577 | ns |
|  |  |  |  | 5 vs. 0 | 0.7844 | ns |
|  |  |  |  | 6 vs. 0 | 0.9592 | ns |
|  |  |  |  | 7 vs. 0 | 0.7726 | ns |
|  |  |  |  | 8 vs. 0 | 0.8103 | ns |
| 28 | ANOVA | 0.0193 | 0.9941 | 1 vs. 0 | 0.7778 | ns |
|  |  |  |  | 2 vs. 0 | 0.8498 | ns |
|  |  |  |  | 3 vs. 0 | 0.8336 | ns |
|  |  |  |  | 4 vs. 0 | 0.8225 | ns |
|  |  |  |  | 5 vs. 0 | 0.7902 | ns |
|  |  |  |  | 6 vs. 0 | 0.9250 | ns |
|  |  |  |  | 7 vs. 0 | 0.7280 | ns |
|  |  |  |  | 8 vs. 0 | 0.7942 | ns |
| 29 | ANOVA | 0.0141 | 0.9958 | 1 vs. 0 | 0.8006 | ns |
|  |  |  |  | 2 vs. 0 | 0.8783 | ns |
|  |  |  |  | 3 vs. 0 | 0.8310 | ns |
|  |  |  |  | 4 vs. 0 | 0.8124 | ns |
|  |  |  |  | 5 vs. 0 | 0.8026 | ns |
|  |  |  |  | 6 vs. 0 | 0.9023 | ns |

| **Day** | **Test** | **Normality p** | **Main p** | **Comparison** | **Pairwise p** | **Significance** |
| --- | --- | --- | --- | --- | --- | --- |
|  |  |  |  | 7 vs. 0 | 0.7134 | ns |
|  |  |  |  | 8 vs. 0 | 0.7842 | ns |
| 30 | ANOVA | 0.0151 | 0.9961 | 1 vs. 0 | 0.7627 | ns |
|  |  |  |  | 2 vs. 0 | 0.8309 | ns |
|  |  |  |  | 3 vs. 0 | 0.8531 | ns |
|  |  |  |  | 4 vs. 0 | 0.8448 | ns |
|  |  |  |  | 5 vs. 0 | 0.8587 | ns |
|  |  |  |  | 6 vs. 0 | 0.9027 | ns |
|  |  |  |  | 7 vs. 0 | 0.7720 | ns |
|  |  |  |  | 8 vs. 0 | 0.8414 | ns |
| 31 | ANOVA | 0.0288 | 0.9946 | 1 vs. 0 | 0.7889 | ns |
|  |  |  |  | 2 vs. 0 | 0.8659 | ns |
|  |  |  |  | 3 vs. 0 | 0.8090 | ns |
|  |  |  |  | 4 vs. 0 | 0.7887 | ns |
|  |  |  |  | 5 vs. 0 | 0.8100 | ns |
|  |  |  |  | 6 vs. 0 | 0.8508 | ns |
|  |  |  |  | 7 vs. 0 | 0.7242 | ns |
|  |  |  |  | 8 vs. 0 | 0.8119 | ns |
| 32 | ANOVA | 0.0149 | 0.9971 | 1 vs. 0 | 0.8931 | ns |
|  |  |  |  | 2 vs. 0 | 0.9402 | ns |
|  |  |  |  | 3 vs. 0 | 0.7480 | ns |
|  |  |  |  | 4 vs. 0 | 0.7445 | ns |
|  |  |  |  | 5 vs. 0 | 0.7453 | ns |
|  |  |  |  | 6 vs. 0 | 0.8101 | ns |
|  |  |  |  | 7 vs. 0 | 0.6748 | ns |
|  |  |  |  | 8 vs. 0 | 0.7954 | ns |

| **Day** | **Test** | **Normality p** | **Main p** | **Comparison** | **Pairwise p** | **Significance** |
| --- | --- | --- | --- | --- | --- | --- |
| 33 | ANOVA | 0.0310 | 0.9980 | 1 vs. 0 | 0.8509 | ns |
|  |  |  |  | 2 vs. 0 | 0.9185 | ns |
|  |  |  |  | 3 vs. 0 | 0.7575 | ns |
|  |  |  |  | 4 vs. 0 | 0.7826 | ns |
|  |  |  |  | 5 vs. 0 | 0.8379 | ns |
|  |  |  |  | 6 vs. 0 | 0.8736 | ns |
|  |  |  |  | 7 vs. 0 | 0.7397 | ns |
|  |  |  |  | 8 vs. 0 | 0.8298 | ns |
| 34 | ANOVA | 0.0218 | 0.9979 | 1 vs. 0 | 0.9085 | ns |
|  |  |  |  | 2 vs. 0 | 0.9878 | ns |
|  |  |  |  | 3 vs. 0 | 0.6970 | ns |
|  |  |  |  | 4 vs. 0 | 0.7303 | ns |
|  |  |  |  | 5 vs. 0 | 0.7722 | ns |
|  |  |  |  | 6 vs. 0 | 0.8182 | ns |
|  |  |  |  | 7 vs. 0 | 0.6735 | ns |
|  |  |  |  | 8 vs. 0 | 0.8132 | ns |
| 35 | ANOVA | 0.0274 | 0.9981 | 1 vs. 0 | 0.8636 | ns |
|  |  |  |  | 2 vs. 0 | 0.9354 | ns |
|  |  |  |  | 3 vs. 0 | 0.7449 | ns |
|  |  |  |  | 4 vs. 0 | 0.7660 | ns |
|  |  |  |  | 5 vs. 0 | 0.8237 | ns |
|  |  |  |  | 6 vs. 0 | 0.8579 | ns |
|  |  |  |  | 7 vs. 0 | 0.7346 | ns |
|  |  |  |  | 8 vs. 0 | 0.8244 | ns |
| 36 | ANOVA | 0.0247 | 0.9986 | 1 vs. 0 | 0.9321 | ns |
|  |  |  |  | 2 vs. 0 | 0.9519 | ns |

| **Day** | **Test** | **Normality p** | **Main p** | **Comparison** | **Pairwise p** | **Significance** |
| --- | --- | --- | --- | --- | --- | --- |
|  |  |  |  | 3 vs. 0 | 0.7440 | ns |
|  |  |  |  | 4 vs. 0 | 0.7374 | ns |
|  |  |  |  | 5 vs. 0 | 0.7825 | ns |
|  |  |  |  | 6 vs. 0 | 0.8647 | ns |
|  |  |  |  | 7 vs. 0 | 0.7104 | ns |
|  |  |  |  | 8 vs. 0 | 0.7876 | ns |
| 37 | ANOVA | 0.0341 | 0.9987 | 1 vs. 0 | 0.9484 | ns |
|  |  |  |  | 2 vs. 0 | 0.9838 | ns |
|  |  |  |  | 3 vs. 0 | 0.7056 | ns |
|  |  |  |  | 4 vs. 0 | 0.7241 | ns |
|  |  |  |  | 5 vs. 0 | 0.7760 | ns |
|  |  |  |  | 6 vs. 0 | 0.8181 | ns |
|  |  |  |  | 7 vs. 0 | 0.6919 | ns |
|  |  |  |  | 8 vs. 0 | 0.7885 | ns |
| 38 | ANOVA | 0.0260 | 0.9981 | 1 vs. 0 | 0.9274 | ns |
|  |  |  |  | 2 vs. 0 | 0.8896 | ns |
|  |  |  |  | 3 vs. 0 | 0.6235 | ns |
|  |  |  |  | 4 vs. 0 | 0.6077 | ns |
|  |  |  |  | 5 vs. 0 | 0.6773 | ns |
|  |  |  |  | 6 vs. 0 | 0.7525 | ns |
|  |  |  |  | 7 vs. 0 | 0.5963 | ns |
|  |  |  |  | 8 vs. 0 | 0.6013 | ns |
| 39 | ANOVA | 0.0196 | 0.9992 | 1 vs. 0 | 0.9437 | ns |
|  |  |  |  | 2 vs. 0 | 0.9205 | ns |
|  |  |  |  | 3 vs. 0 | 0.6294 | ns |
|  |  |  |  | 4 vs. 0 | 0.6583 | ns |

| **Day** | **Test** | **Normality p** | **Main p** | **Comparison** | **Pairwise p** | **Significance** |
| --- | --- | --- | --- | --- | --- | --- |
|  |  |  |  | 5 vs. 0 | 0.7571 | ns |
|  |  |  |  | 6 vs. 0 | 0.8434 | ns |
|  |  |  |  | 7 vs. 0 | 0.7084 | ns |
|  |  |  |  | 8 vs. 0 | 0.6980 | ns |
| 40 | ANOVA | 0.0293 | 0.9989 | 1 vs. 0 | 0.9106 | ns |
|  |  |  |  | 2 vs. 0 | 0.8934 | ns |
|  |  |  |  | 3 vs. 0 | 0.5938 | ns |
|  |  |  |  | 4 vs. 0 | 0.6093 | ns |
|  |  |  |  | 5 vs. 0 | 0.7661 | ns |
|  |  |  |  | 6 vs. 0 | 0.8007 | ns |
|  |  |  |  | 7 vs. 0 | 0.6699 | ns |
|  |  |  |  | 8 vs. 0 | 0.7261 | ns |
| 41 | ANOVA | 0.0256 | 0.9972 | 1 vs. 0 | 0.9169 | ns |
|  |  |  |  | 2 vs. 0 | 0.8480 | ns |
|  |  |  |  | 3 vs. 0 | 0.5332 | ns |
|  |  |  |  | 4 vs. 0 | 0.5604 | ns |
|  |  |  |  | 5 vs. 0 | 0.6835 | ns |
|  |  |  |  | 6 vs. 0 | 0.7335 | ns |
|  |  |  |  | 7 vs. 0 | 0.6097 | ns |
|  |  |  |  | 8 vs. 0 | 0.6478 | ns |
| 42 | ANOVA | 0.0325 | 0.9981 | 1 vs. 0 | 0.9558 | ns |
|  |  |  |  | 2 vs. 0 | 0.8947 | ns |
|  |  |  |  | 3 vs. 0 | 0.5851 | ns |
|  |  |  |  | 4 vs. 0 | 0.6099 | ns |
|  |  |  |  | 5 vs. 0 | 0.7501 | ns |
|  |  |  |  | 6 vs. 0 | 0.7976 | ns |

| **Day** | **Test** | **Normality p** | **Main p** | **Comparison** | **Pairwise p** | **Significance** |
| --- | --- | --- | --- | --- | --- | --- |
|  |  |  |  | 7 vs. 0 | 0.6629 | ns |
|  |  |  |  | 8 vs. 0 | 0.6805 | ns |
| 43 | ANOVA | 0.0330 | 0.9982 | 1 vs. 0 | 0.9062 | ns |
|  |  |  |  | 2 vs. 0 | 0.8831 | ns |
|  |  |  |  | 3 vs. 0 | 0.5673 | ns |
|  |  |  |  | 4 vs. 0 | 0.5866 | ns |
|  |  |  |  | 5 vs. 0 | 0.7135 | ns |
|  |  |  |  | 6 vs. 0 | 0.7474 | ns |
|  |  |  |  | 7 vs. 0 | 0.6350 | ns |
|  |  |  |  | 8 vs. 0 | 0.6584 | ns |
| 44 | ANOVA | 0.0354 | 0.9970 | 1 vs. 0 | 0.8276 | ns |
|  |  |  |  | 2 vs. 0 | 0.7987 | ns |
|  |  |  |  | 3 vs. 0 | 0.4868 | ns |
|  |  |  |  | 4 vs. 0 | 0.5095 | ns |
|  |  |  |  | 5 vs. 0 | 0.6341 | ns |
|  |  |  |  | 6 vs. 0 | 0.6733 | ns |
|  |  |  |  | 7 vs. 0 | 0.5805 | ns |
|  |  |  |  | 8 vs. 0 | 0.5881 | ns |
| 45 | ANOVA | 0.0314 | 0.8518 | 1 vs. 0 | 0.4475 | ns |
|  |  |  |  | 2 vs. 0 | 0.4279 | ns |
|  |  |  |  | 3 vs. 0 | 0.2949 | ns |
|  |  |  |  | 4 vs. 0 | 0.3270 | ns |

| **Day** | **Test** | **Normality p** | **Main p** | **Comparison** | **Pairwise p** | **Significance** |
| --- | --- | --- | --- | --- | --- | --- |
| 5 | ANOVA | 0.0156 | 0.0027 | 3 vs. 0 | 0.0469 | p<0.05 |
| 6 | ANOVA | 0.0021 | <.0001 | 1 vs. 0 | 0.0020 | p<0.05 |
|  |  |  |  | 2 vs. 0 | 0.0006 | p<0.05 |
|  |  |  |  | 3 vs. 0 | 0.0036 | p<0.05 |
|  |  |  |  | 4 vs. 0 | 0.0228 | p<0.05 |
| 7 | Kruskal-Wallis | 0.0001 | <.0001 | 1 vs. 0 | 0.0383 | p<0.05 |
|  |  |  |  | 2 vs. 0 | 0.0154 | p<0.05 |
|  |  |  |  | 3 vs. 0 | 0.0370 | p<0.05 |
| 8 | ANOVA | 0.0088 | <.0001 | 1 vs. 0 | <.0001 | p<0.05 |
|  |  |  |  | 2 vs. 0 | <.0001 | p<0.05 |
|  |  |  |  | 3 vs. 0 | 0.0006 | p<0.05 |
|  |  |  |  | 4 vs. 0 | 0.0013 | p<0.05 |
|  |  |  |  | 5 vs. 0 | 0.0445 | p<0.05 |
| 9 | ANOVA | 0.0036 | <.0001 | 1 vs. 0 | <.0001 | p<0.05 |
|  |  |  |  | 2 vs. 0 | <.0001 | p<0.05 |
|  |  |  |  | 3 vs. 0 | <.0001 | p<0.05 |
|  |  |  |  | 4 vs. 0 | <.0001 | p<0.05 |
|  |  |  |  | 6 vs. 0 | 0.0487 | p<0.05 |
| 16 | ANOVA | 0.0013 | 0.1790 | 4 vs. 0 | 0.0494 | p<0.05 |
|  |  |  |  | 8 vs. 0 | 0.0299 | p<0.05 |
| 20 | ANOVA | 0.0198 | 0.1947 | 8 vs. 0 | 0.0386 | p<0.05 |

| **Day** | **Test** | **Normality p** | **Main p** | **Comparison** | **Pairwise p** | **Significance** |
| --- | --- | --- | --- | --- | --- | --- |
| 5 | ANOVA | 0.0082 | <.0001 | 1 vs. 0 | 0.0001 | p<0.05 |
|  |  |  |  | 2 vs. 0 | <.0001 | p<0.05 |
|  |  |  |  | 4 vs. 0 | 0.0202 | p<0.05 |
| 6 | ANOVA | 0.2591 | <.0001 | 1 vs. 0 | 0.0002 | p<0.05 |
|  |  |  |  | 2 vs. 0 | <.0001 | p<0.05 |
|  |  |  |  | 3 vs. 0 | 0.0077 | p<0.05 |
|  |  |  |  | 4 vs. 0 | 0.0365 | p<0.05 |
| 7 | ANOVA | 0.4499 | 0.0021 | 1 vs. 0 | 0.0204 | p<0.05 |
|  |  |  |  | 2 vs. 0 | <.0001 | p<0.05 |
|  |  |  |  | 6 vs. 0 | 0.0233 | p<0.05 |
|  |  |  |  | 8 vs. 0 | 0.0262 | p<0.05 |
| 9 | ANOVA | 0.1982 | 0.0347 | 1 vs. 0 | 0.0070 | p<0.05 |
|  |  |  |  | 2 vs. 0 | 0.0184 | p<0.05 |
|  |  |  |  | 3 vs. 0 | 0.0454 | p<0.05 |
|  |  |  |  | 4 vs. 0 | 0.0441 | p<0.05 |
| 12 | ANOVA | 0.0396 | 0.0046 | 4 vs. 0 | 0.0044 | p<0.05 |
|  |  |  |  | 5 vs. 0 | 0.0230 | p<0.05 |
|  |  |  |  | 6 vs. 0 | 0.0026 | p<0.05 |
|  |  |  |  | 7 vs. 0 | 0.0090 | p<0.05 |
| 14 | ANOVA | 0.5247 | 0.0934 | 3 vs. 0 | 0.0335 | p<0.05 |
|  |  |  |  | 4 vs. 0 | 0.0177 | p<0.05 |
|  |  |  |  | 5 vs. 0 | 0.0390 | p<0.05 |
|  |  |  |  | 6 vs. 0 | 0.0105 | p<0.05 |
|  |  |  |  | 7 vs. 0 | 0.0252 | p<0.05 |
| 45 | ANOVA | 0.0840 | 0.3317 | 4 vs. 0 | 0.0493 | p<0.05 |
| -29 | ANOVA | 1.0000 | 0.1703 |  | . | n/a |

| **Day** | **Test** | **Normality p** | **Main p** | **Comparison** | **Pairwise p** | **Significance** |
| --- | --- | --- | --- | --- | --- | --- |
| -30 | ANOVA | 0.2997 | 0.0387 |  | . | n/a |
| -31 | ANOVA | 0.4456 | 0.9393 |  | . | n/a |

| **Day** | **Test** | **Normality p** | **Main p** | **Comparison** | **Pairwise p** | **Significance** |
| --- | --- | --- | --- | --- | --- | --- |
| 5 | ANOVA | 0.0054 | 0.0576 | 2 vs. 0 | 0.0380 | p<0.05 |
| 6 | ANOVA | 0.2804 | 0.0077 | 1 vs. 0 | 0.0023 | p<0.05 |
|  |  |  |  | 2 vs. 0 | 0.0089 | p<0.05 |
| 7 | ANOVA | 0.4587 | 0.0407 | 1 vs. 0 | 0.0253 | p<0.05 |
|  |  |  |  | 2 vs. 0 | 0.0328 | p<0.05 |
| 8 | ANOVA | 0.0640 | 0.0229 | 1 vs. 0 | 0.0045 | p<0.05 |
|  |  |  |  | 2 vs. 0 | 0.0060 | p<0.05 |
|  |  |  |  | 4 vs. 0 | 0.0454 | p<0.05 |

| **Day** | **Test** | **Normality p** | **Main p** | **Comparison** | **Pairwise p** | **Significance** |
| --- | --- | --- | --- | --- | --- | --- |
| 4 | ANOVA | 0.8239 | 0.1265 | 5 vs. 0 | 0.0396 | p<0.05 |
|  |  |  |  | 8 vs. 0 | 0.0131 | p<0.05 |
| 5 | ANOVA | 0.9965 | <.0001 | 1 vs. 0 | 0.0036 | p<0.05 |
|  |  |  |  | 2 vs. 0 | <.0001 | p<0.05 |
|  |  |  |  | 3 vs. 0 | 0.0022 | p<0.05 |
| 6 | ANOVA | 0.4940 | 0.0008 | 1 vs. 0 | 0.0013 | p<0.05 |
|  |  |  |  | 2 vs. 0 | 0.0029 | p<0.05 |
|  |  |  |  | 3 vs. 0 | 0.0058 | p<0.05 |
| 7 | ANOVA | 0.6413 | 0.0033 | 2 vs. 0 | 0.0349 | p<0.05 |
|  |  |  |  | 8 vs. 0 | 0.0185 | p<0.05 |
| 8 | ANOVA | 0.0196 | 0.0112 | 1 vs. 0 | 0.0172 | p<0.05 |
|  |  |  |  | 2 vs. 0 | 0.0010 | p<0.05 |
|  |  |  |  | 4 vs. 0 | 0.0144 | p<0.05 |
| 9 | ANOVA | 0.0149 | <.0001 | 1 vs. 0 | <.0001 | p<0.05 |
|  |  |  |  | 2 vs. 0 | <.0001 | p<0.05 |
|  |  |  |  | 3 vs. 0 | <.0001 | p<0.05 |
|  |  |  |  | 4 vs. 0 | <.0001 | p<0.05 |
|  |  |  |  | 6 vs. 0 | <.0001 | p<0.05 |
| 10 | ANOVA | 0.0317 | <.0001 | 1 vs. 0 | 0.0068 | p<0.05 |
|  |  |  |  | 2 vs. 0 | 0.0051 | p<0.05 |
|  |  |  |  | 3 vs. 0 | 0.0036 | p<0.05 |
|  |  |  |  | 4 vs. 0 | 0.0059 | p<0.05 |
|  |  |  |  | 8 vs. 0 | 0.0126 | p<0.05 |
| 28 | ANOVA | 0.0255 | 0.2460 | 3 vs. 0 | 0.0109 | p<0.05 |
|  |  |  |  | 4 vs. 0 | 0.0493 | p<0.05 |
|  |  |  |  | 6 vs. 0 | 0.0417 | p<0.05 |

| **Day** | **Test** | **Normality p** | **Main p** | **Comparison** | **Pairwise p** | **Significance** |
| --- | --- | --- | --- | --- | --- | --- |
|  |  |  |  | 7 vs. 0 | 0.0417 | p<0.05 |
| 44 | ANOVA | 0.5457 | 0.0252 | 4 vs. 0 | 0.0015 | p<0.05 |
|  |  |  |  | 5 vs. 0 | 0.0054 | p<0.05 |
|  |  |  |  | 6 vs. 0 | 0.0164 | p<0.05 |
|  |  |  |  | 7 vs. 0 | 0.0103 | p<0.05 |
| -34 | ANOVA | 0.6412 | 0.0406 | 7 vs. 0 | 0.0322 | p<0.05 |

| **Day** | **Test** | **Normality p** | **Main p** | **Comparison** | **Pairwise p** | **Significance** |
| --- | --- | --- | --- | --- | --- | --- |
| 4 | ANOVA | 0.7702 | 0.1717 | 5 vs. 0 | 0.0314 | p<0.05 |
|  |  |  |  | 8 vs. 0 | 0.0139 | p<0.05 |
| 5 | ANOVA | 0.4286 | 0.0033 | 2 vs. 0 | 0.0386 | p<0.05 |
|  |  |  |  | 3 vs. 0 | 0.0274 | p<0.05 |
| 7 | ANOVA | 0.7493 | 0.0658 | 8 vs. 0 | 0.0092 | p<0.05 |
| 8 | ANOVA | 0.0820 | 0.0232 | 1 vs. 0 | 0.0263 | p<0.05 |
|  |  |  |  | 2 vs. 0 | 0.0130 | p<0.05 |
|  |  |  |  | 4 vs. 0 | 0.0358 | p<0.05 |
| 9 | ANOVA | 0.0040 | <.0001 | 1 vs. 0 | <.0001 | p<0.05 |
|  |  |  |  | 2 vs. 0 | <.0001 | p<0.05 |
|  |  |  |  | 3 vs. 0 | <.0001 | p<0.05 |
|  |  |  |  | 4 vs. 0 | <.0001 | p<0.05 |
|  |  |  |  | 6 vs. 0 | 0.0082 | p<0.05 |
| 10 | ANOVA | 0.0204 | 0.0283 | 1 vs. 0 | 0.0351 | p<0.05 |
|  |  |  |  | 2 vs. 0 | 0.0483 | p<0.05 |

| **Day** | **Test** | **Normality p** | **Main p** | **Comparison** | **Pairwise p** | **Significance** |
| --- | --- | --- | --- | --- | --- | --- |
| 5 | Kruskal-Wallis | 0.0001 | <.0001 | 1 vs. 0 | 0.0340 | p<0.05 |
|  |  |  |  | 2 vs. 0 | 0.0088 | p<0.05 |
|  |  |  |  | 3 vs. 0 | 0.0026 | p<0.05 |
| 6 | Kruskal-Wallis | 0.0001 | <.0001 | 2 vs. 0 | 0.0303 | p<0.05 |
| 7 | Kruskal-Wallis | 0.0001 | <.0001 | 1 vs. 0 | 0.0279 | p<0.05 |
|  |  |  |  | 2 vs. 0 | 0.0075 | p<0.05 |
|  |  |  |  | 3 vs. 0 | 0.0055 | p<0.05 |
|  |  |  |  | 4 vs. 0 | 0.0055 | p<0.05 |
| 8 | Kruskal-Wallis | 0.0001 | <.0001 | 1 vs. 0 | 0.0284 | p<0.05 |
|  |  |  |  | 2 vs. 0 | 0.0088 | p<0.05 |
|  |  |  |  | 3 vs. 0 | 0.0062 | p<0.05 |
|  |  |  |  | 4 vs. 0 | 0.0157 | p<0.05 |
| 9 | Kruskal-Wallis | 0.0001 | 0.0003 | 1 vs. 0 | 0.0277 | p<0.05 |
|  |  |  |  | 2 vs. 0 | 0.0197 | p<0.05 |
|  |  |  |  | 3 vs. 0 | 0.0248 | p<0.05 |
|  |  |  |  | 4 vs. 0 | 0.0248 | p<0.05 |
| 10 | Kruskal-Wallis | 0.0001 | <.0001 | 2 vs. 0 | 0.0169 | p<0.05 |
|  |  |  |  | 3 vs. 0 | 0.0067 | p<0.05 |
|  |  |  |  | 4 vs. 0 | 0.0067 | p<0.05 |
| 14 | Kruskal-Wallis | 0.0001 | 0.0131 | 3 vs. 0 | 0.0230 | p<0.05 |
| 16 | ANOVA | 0.0018 | <.0001 | 4 vs. 0 | <.0001 | p<0.05 |
|  |  |  |  | 5 vs. 0 | <.0001 | p<0.05 |
|  |  |  |  | 6 vs. 0 | <.0001 | p<0.05 |
|  |  |  |  | 7 vs. 0 | <.0001 | p<0.05 |
|  |  |  |  | 8 vs. 0 | <.0001 | p<0.05 |
| 20 | ANOVA | 0.0013 | 0.0732 | 4 vs. 0 | 0.0254 | p<0.05 |

| **Day** | **Test** | **Normality p** | **Main p** | **Comparison** | **Pairwise p** | **Significance** |
| --- | --- | --- | --- | --- | --- | --- |
|  |  |  |  | 5 vs. 0 | 0.0060 | p<0.05 |
|  |  |  |  | 6 vs. 0 | 0.0044 | p<0.05 |
|  |  |  |  | 7 vs. 0 | 0.0078 | p<0.05 |
|  |  |  |  | 8 vs. 0 | 0.0308 | p<0.05 |
| 21 | Kruskal-Wallis | 0.0001 | <.0001 | 1 vs. 0 | 0.0455 | p<0.05 |
|  |  |  |  | 2 vs. 0 | 0.0286 | p<0.05 |
|  |  |  |  | 3 vs. 0 | 0.0187 | p<0.05 |
|  |  |  |  | 4 vs. 0 | 0.0187 | p<0.05 |

| **Day** | **Test** | **Normality p** | **Main p** | **Comparison** | **Pairwise p** | **Significance** |
| --- | --- | --- | --- | --- | --- | --- |
| 4 | ANOVA | 0.3168 | <.0001 | 1 vs. 0 | 0.0046 | p<0.05 |
|  |  |  |  | 2 vs. 0 | 0.0367 | p<0.05 |
|  |  |  |  | 3 vs. 0 | 0.0032 | p<0.05 |
|  |  |  |  | 6 vs. 0 | 0.0089 | p<0.05 |
|  |  |  |  | 8 vs. 0 | 0.0001 | p<0.05 |
| 5 | ANOVA | 0.4158 | <.0001 | 1 vs. 0 | <.0001 | p<0.05 |
|  |  |  |  | 2 vs. 0 | <.0001 | p<0.05 |
|  |  |  |  | 3 vs. 0 | <.0001 | p<0.05 |
|  |  |  |  | 4 vs. 0 | <.0001 | p<0.05 |
|  |  |  |  | 7 vs. 0 | 0.0468 | p<0.05 |
| 6 | ANOVA | 0.2376 | <.0001 | 1 vs. 0 | <.0001 | p<0.05 |
|  |  |  |  | 2 vs. 0 | <.0001 | p<0.05 |
|  |  |  |  | 3 vs. 0 | <.0001 | p<0.05 |
|  |  |  |  | 4 vs. 0 | <.0001 | p<0.05 |
| 7 | ANOVA | 0.0699 | <.0001 | 1 vs. 0 | <.0001 | p<0.05 |
|  |  |  |  | 2 vs. 0 | <.0001 | p<0.05 |
|  |  |  |  | 3 vs. 0 | <.0001 | p<0.05 |
|  |  |  |  | 4 vs. 0 | <.0001 | p<0.05 |
| 8 | ANOVA | 0.0630 | <.0001 | 1 vs. 0 | <.0001 | p<0.05 |
|  |  |  |  | 2 vs. 0 | <.0001 | p<0.05 |
|  |  |  |  | 3 vs. 0 | <.0001 | p<0.05 |
|  |  |  |  | 4 vs. 0 | <.0001 | p<0.05 |
|  |  |  |  | 7 vs. 0 | 0.0024 | p<0.05 |
| 9 | Kruskal-Wallis | 0.0001 | <.0001 | 1 vs. 0 | 0.0372 | p<0.05 |
|  |  |  |  | 2 vs. 0 | 0.0160 | p<0.05 |
|  |  |  |  | 3 vs. 0 | 0.0093 | p<0.05 |

| **Day** | **Test** | **Normality p** | **Main p** | **Comparison** | **Pairwise p** | **Significance** |
| --- | --- | --- | --- | --- | --- | --- |
|  |  |  |  | 4 vs. 0 | 0.0122 | p<0.05 |
|  |  |  |  | 7 vs. 0 | 0.0385 | p<0.05 |
| 10 | ANOVA | 0.0093 | <.0001 | 1 vs. 0 | <.0001 | p<0.05 |
|  |  |  |  | 2 vs. 0 | <.0001 | p<0.05 |
|  |  |  |  | 3 vs. 0 | <.0001 | p<0.05 |
|  |  |  |  | 4 vs. 0 | <.0001 | p<0.05 |
|  |  |  |  | 5 vs. 0 | 0.0133 | p<0.05 |
|  |  |  |  | 6 vs. 0 | 0.0454 | p<0.05 |
|  |  |  |  | 7 vs. 0 | 0.0113 | p<0.05 |
| 11 | ANOVA | 0.0076 | <.0001 | 1 vs. 0 | <.0001 | p<0.05 |
|  |  |  |  | 2 vs. 0 | <.0001 | p<0.05 |
|  |  |  |  | 3 vs. 0 | <.0001 | p<0.05 |
|  |  |  |  | 4 vs. 0 | <.0001 | p<0.05 |
|  |  |  |  | 5 vs. 0 | 0.0114 | p<0.05 |
|  |  |  |  | 6 vs. 0 | 0.0082 | p<0.05 |
| 12 | ANOVA | 0.0759 | <.0001 | 1 vs. 0 | <.0001 | p<0.05 |
|  |  |  |  | 2 vs. 0 | <.0001 | p<0.05 |
|  |  |  |  | 3 vs. 0 | <.0001 | p<0.05 |
|  |  |  |  | 4 vs. 0 | <.0001 | p<0.05 |
|  |  |  |  | 5 vs. 0 | 0.0006 | p<0.05 |
|  |  |  |  | 6 vs. 0 | 0.0007 | p<0.05 |
|  |  |  |  | 7 vs. 0 | 0.0044 | p<0.05 |
|  |  |  |  | 8 vs. 0 | 0.0074 | p<0.05 |
| 13 | ANOVA | 0.5931 | <.0001 | 1 vs. 0 | <.0001 | p<0.05 |
|  |  |  |  | 2 vs. 0 | <.0001 | p<0.05 |
|  |  |  |  | 3 vs. 0 | <.0001 | p<0.05 |

| **Day** | **Test** | **Normality p** | **Main p** | **Comparison** | **Pairwise p** | **Significance** |
| --- | --- | --- | --- | --- | --- | --- |
|  |  |  |  | 4 vs. 0 | <.0001 | p<0.05 |
|  |  |  |  | 5 vs. 0 | <.0001 | p<0.05 |
|  |  |  |  | 6 vs. 0 | <.0001 | p<0.05 |
|  |  |  |  | 7 vs. 0 | <.0001 | p<0.05 |
|  |  |  |  | 8 vs. 0 | 0.0001 | p<0.05 |
| 14 | ANOVA | 0.0262 | <.0001 | 1 vs. 0 | <.0001 | p<0.05 |
|  |  |  |  | 2 vs. 0 | <.0001 | p<0.05 |
|  |  |  |  | 3 vs. 0 | <.0001 | p<0.05 |
|  |  |  |  | 4 vs. 0 | <.0001 | p<0.05 |
|  |  |  |  | 5 vs. 0 | <.0001 | p<0.05 |
|  |  |  |  | 6 vs. 0 | <.0001 | p<0.05 |
|  |  |  |  | 7 vs. 0 | <.0001 | p<0.05 |
|  |  |  |  | 8 vs. 0 | 0.0009 | p<0.05 |
| 15 | ANOVA | 0.0055 | <.0001 | 1 vs. 0 | <.0001 | p<0.05 |
|  |  |  |  | 2 vs. 0 | <.0001 | p<0.05 |
|  |  |  |  | 3 vs. 0 | <.0001 | p<0.05 |
|  |  |  |  | 4 vs. 0 | <.0001 | p<0.05 |
|  |  |  |  | 5 vs. 0 | <.0001 | p<0.05 |
|  |  |  |  | 6 vs. 0 | <.0001 | p<0.05 |
|  |  |  |  | 7 vs. 0 | <.0001 | p<0.05 |
|  |  |  |  | 8 vs. 0 | <.0001 | p<0.05 |
| 16 | ANOVA | 0.0139 | <.0001 | 1 vs. 0 | <.0001 | p<0.05 |
|  |  |  |  | 2 vs. 0 | <.0001 | p<0.05 |
|  |  |  |  | 3 vs. 0 | <.0001 | p<0.05 |
|  |  |  |  | 4 vs. 0 | <.0001 | p<0.05 |
|  |  |  |  | 5 vs. 0 | <.0001 | p<0.05 |

| **Day** | **Test** | **Normality p** | **Main p** | **Comparison** | **Pairwise p** | **Significance** |
| --- | --- | --- | --- | --- | --- | --- |
|  |  |  |  | 6 vs. 0 | <.0001 | p<0.05 |
|  |  |  |  | 7 vs. 0 | <.0001 | p<0.05 |
|  |  |  |  | 8 vs. 0 | <.0001 | p<0.05 |
| 17 | ANOVA | 0.0297 | <.0001 | 1 vs. 0 | <.0001 | p<0.05 |
|  |  |  |  | 2 vs. 0 | <.0001 | p<0.05 |
|  |  |  |  | 3 vs. 0 | <.0001 | p<0.05 |
|  |  |  |  | 4 vs. 0 | <.0001 | p<0.05 |
|  |  |  |  | 5 vs. 0 | <.0001 | p<0.05 |
|  |  |  |  | 6 vs. 0 | <.0001 | p<0.05 |
|  |  |  |  | 7 vs. 0 | <.0001 | p<0.05 |
|  |  |  |  | 8 vs. 0 | 0.0007 | p<0.05 |
| 28 | ANOVA | 0.0076 | 0.0016 | 1 vs. 0 | <.0001 | p<0.05 |
|  |  |  |  | 2 vs. 0 | <.0001 | p<0.05 |
|  |  |  |  | 3 vs. 0 | <.0001 | p<0.05 |
|  |  |  |  | 4 vs. 0 | <.0001 | p<0.05 |
|  |  |  |  | 5 vs. 0 | 0.0004 | p<0.05 |
|  |  |  |  | 6 vs. 0 | 0.0014 | p<0.05 |
|  |  |  |  | 7 vs. 0 | 0.0001 | p<0.05 |
|  |  |  |  | 8 vs. 0 | 0.0422 | p<0.05 |
| 30 | ANOVA | 0.0119 | <.0001 | 1 vs. 0 | <.0001 | p<0.05 |
|  |  |  |  | 2 vs. 0 | <.0001 | p<0.05 |
|  |  |  |  | 3 vs. 0 | <.0001 | p<0.05 |
|  |  |  |  | 4 vs. 0 | <.0001 | p<0.05 |
|  |  |  |  | 5 vs. 0 | <.0001 | p<0.05 |
|  |  |  |  | 6 vs. 0 | <.0001 | p<0.05 |
|  |  |  |  | 7 vs. 0 | <.0001 | p<0.05 |

| **Day** | **Test** | **Normality p** | **Main p** | **Comparison** | **Pairwise p** | **Significance** |
| --- | --- | --- | --- | --- | --- | --- |
|  |  |  |  | 8 vs. 0 | 0.0001 | p<0.05 |
| 31 | ANOVA | 0.0025 | 0.0007 | 1 vs. 0 | <.0001 | p<0.05 |
|  |  |  |  | 2 vs. 0 | 0.0001 | p<0.05 |
|  |  |  |  | 3 vs. 0 | <.0001 | p<0.05 |
|  |  |  |  | 4 vs. 0 | <.0001 | p<0.05 |
|  |  |  |  | 5 vs. 0 | 0.0001 | p<0.05 |
|  |  |  |  | 6 vs. 0 | 0.0143 | p<0.05 |
|  |  |  |  | 7 vs. 0 | 0.0002 | p<0.05 |
|  |  |  |  | 8 vs. 0 | 0.0056 | p<0.05 |
| 32 | ANOVA | 0.0075 | 0.0008 | 1 vs. 0 | <.0001 | p<0.05 |
|  |  |  |  | 2 vs. 0 | <.0001 | p<0.05 |
|  |  |  |  | 3 vs. 0 | <.0001 | p<0.05 |
|  |  |  |  | 4 vs. 0 | <.0001 | p<0.05 |
|  |  |  |  | 5 vs. 0 | 0.0002 | p<0.05 |
|  |  |  |  | 6 vs. 0 | 0.0079 | p<0.05 |
|  |  |  |  | 7 vs. 0 | 0.0023 | p<0.05 |
|  |  |  |  | 8 vs. 0 | 0.0181 | p<0.05 |
| 33 | ANOVA | 0.0013 | 0.0046 | 1 vs. 0 | 0.0006 | p<0.05 |
|  |  |  |  | 2 vs. 0 | <.0001 | p<0.05 |
|  |  |  |  | 3 vs. 0 | 0.0003 | p<0.05 |
|  |  |  |  | 4 vs. 0 | 0.0002 | p<0.05 |
|  |  |  |  | 5 vs. 0 | 0.0002 | p<0.05 |
|  |  |  |  | 6 vs. 0 | 0.0178 | p<0.05 |
|  |  |  |  | 7 vs. 0 | 0.0017 | p<0.05 |
|  |  |  |  | 8 vs. 0 | 0.0280 | p<0.05 |
| 34 | ANOVA | 0.0353 | 0.0002 | 1 vs. 0 | 0.0001 | p<0.05 |

| **Day** | **Test** | **Normality p** | **Main p** | **Comparison** | **Pairwise p** | **Significance** |
| --- | --- | --- | --- | --- | --- | --- |
|  |  |  |  | 2 vs. 0 | <.0001 | p<0.05 |
|  |  |  |  | 3 vs. 0 | <.0001 | p<0.05 |
|  |  |  |  | 4 vs. 0 | <.0001 | p<0.05 |
|  |  |  |  | 5 vs. 0 | 0.0002 | p<0.05 |
|  |  |  |  | 6 vs. 0 | 0.0255 | p<0.05 |
|  |  |  |  | 7 vs. 0 | 0.0092 | p<0.05 |
|  |  |  |  | 8 vs. 0 | 0.0278 | p<0.05 |
| 35 | ANOVA | 0.0014 | 0.0002 | 1 vs. 0 | <.0001 | p<0.05 |
|  |  |  |  | 2 vs. 0 | <.0001 | p<0.05 |
|  |  |  |  | 3 vs. 0 | <.0001 | p<0.05 |
|  |  |  |  | 4 vs. 0 | <.0001 | p<0.05 |
|  |  |  |  | 5 vs. 0 | <.0001 | p<0.05 |
|  |  |  |  | 6 vs. 0 | 0.0014 | p<0.05 |
|  |  |  |  | 7 vs. 0 | 0.0003 | p<0.05 |
|  |  |  |  | 8 vs. 0 | 0.0028 | p<0.05 |
| 36 | ANOVA | 0.2512 | 0.0224 | 2 vs. 0 | 0.0247 | p<0.05 |
|  |  |  |  | 3 vs. 0 | 0.0090 | p<0.05 |
|  |  |  |  | 4 vs. 0 | 0.0094 | p<0.05 |
|  |  |  |  | 5 vs. 0 | 0.0201 | p<0.05 |
| 37 | ANOVA | 0.5435 | <.0001 | 1 vs. 0 | <.0001 | p<0.05 |
|  |  |  |  | 2 vs. 0 | <.0001 | p<0.05 |
|  |  |  |  | 3 vs. 0 | <.0001 | p<0.05 |
|  |  |  |  | 4 vs. 0 | <.0001 | p<0.05 |
|  |  |  |  | 5 vs. 0 | <.0001 | p<0.05 |
|  |  |  |  | 6 vs. 0 | 0.0004 | p<0.05 |
|  |  |  |  | 7 vs. 0 | 0.0007 | p<0.05 |

| **Day** | **Test** | **Normality p** | **Main p** | **Comparison** | **Pairwise p** | **Significance** |
| --- | --- | --- | --- | --- | --- | --- |
|  |  |  |  | 8 vs. 0 | 0.0012 | p<0.05 |
| 38 | ANOVA | 0.0018 | 0.0021 | 1 vs. 0 | 0.0012 | p<0.05 |
|  |  |  |  | 2 vs. 0 | 0.0011 | p<0.05 |
|  |  |  |  | 3 vs. 0 | 0.0001 | p<0.05 |
|  |  |  |  | 4 vs. 0 | <.0001 | p<0.05 |
|  |  |  |  | 5 vs. 0 | 0.0008 | p<0.05 |
|  |  |  |  | 6 vs. 0 | 0.0091 | p<0.05 |
|  |  |  |  | 7 vs. 0 | 0.0074 | p<0.05 |
| 40 | ANOVA | 0.0018 | 0.0099 | 1 vs. 0 | 0.0006 | p<0.05 |
|  |  |  |  | 2 vs. 0 | 0.0014 | p<0.05 |
|  |  |  |  | 3 vs. 0 | 0.0003 | p<0.05 |
|  |  |  |  | 4 vs. 0 | 0.0001 | p<0.05 |
|  |  |  |  | 5 vs. 0 | 0.0004 | p<0.05 |
|  |  |  |  | 6 vs. 0 | 0.0014 | p<0.05 |
|  |  |  |  | 7 vs. 0 | 0.0122 | p<0.05 |
|  |  |  |  | 8 vs. 0 | 0.0155 | p<0.05 |
| 41 | ANOVA | 0.0034 | 0.0378 | 1 vs. 0 | 0.0044 | p<0.05 |
|  |  |  |  | 2 vs. 0 | 0.0261 | p<0.05 |
|  |  |  |  | 3 vs. 0 | 0.0009 | p<0.05 |
|  |  |  |  | 4 vs. 0 | 0.0036 | p<0.05 |
|  |  |  |  | 5 vs. 0 | 0.0016 | p<0.05 |
|  |  |  |  | 6 vs. 0 | 0.0161 | p<0.05 |
|  |  |  |  | 7 vs. 0 | 0.0320 | p<0.05 |
| 42 | ANOVA | 0.0016 | 0.0170 | 1 vs. 0 | 0.0024 | p<0.05 |
|  |  |  |  | 2 vs. 0 | 0.0034 | p<0.05 |
|  |  |  |  | 3 vs. 0 | 0.0004 | p<0.05 |

| **Day** | **Test** | **Normality p** | **Main p** | **Comparison** | **Pairwise p** | **Significance** |
| --- | --- | --- | --- | --- | --- | --- |
|  |  |  |  | 4 vs. 0 | 0.0037 | p<0.05 |
|  |  |  |  | 5 vs. 0 | 0.0034 | p<0.05 |
|  |  |  |  | 6 vs. 0 | 0.0250 | p<0.05 |
| 43 | ANOVA | 0.0133 | 0.0050 | 1 vs. 0 | 0.0002 | p<0.05 |
|  |  |  |  | 2 vs. 0 | 0.0004 | p<0.05 |
|  |  |  |  | 3 vs. 0 | <.0001 | p<0.05 |
|  |  |  |  | 4 vs. 0 | <.0001 | p<0.05 |
|  |  |  |  | 5 vs. 0 | 0.0010 | p<0.05 |
|  |  |  |  | 6 vs. 0 | 0.0015 | p<0.05 |
|  |  |  |  | 7 vs. 0 | 0.0029 | p<0.05 |
|  |  |  |  | 8 vs. 0 | 0.0110 | p<0.05 |

| **Day** | **Test** | **Normality p** | **Main p** | **Comparison** | **Pairwise p** | **Significance** |
| --- | --- | --- | --- | --- | --- | --- |
| 21 | Kruskal-Wallis | 0.0001 | <.0001 | 1 vs. 0 | 0.0455 | p<0.05 |
|  |  |  |  | 2 vs. 0 | 0.0286 | p<0.05 |
|  |  |  |  | 3 vs. 0 | 0.0187 | p<0.05 |
|  |  |  |  | 4 vs. 0 | 0.0187 | p<0.05 |
| 45 | Kruskal-Wallis | 0.0001 | <.0001 | 1 vs. 0 | 0.0455 | p<0.05 |
|  |  |  |  | 2 vs. 0 | 0.0286 | p<0.05 |
|  |  |  |  | 3 vs. 0 | 0.0187 | p<0.05 |
|  |  |  |  | 4 vs. 0 | 0.0187 | p<0.05 |

| **Day** | **Test** | **Normality p** | **Main p** | **Comparison** | **Pairwise p** | **Significance** |
| --- | --- | --- | --- | --- | --- | --- |
| 2 | ANOVA | 0.9662 | 0.1995 | 1 vs. 0 | 0.0260 | p<0.05 |
| 3 | ANOVA | 0.4537 | 0.0754 | 1 vs. 0 | 0.0438 | p<0.05 |
| 4 | ANOVA | 0.7954 | 0.0005 | 1 vs. 0 | 0.0223 | p<0.05 |
|  |  |  |  | 8 vs. 0 | 0.0377 | p<0.05 |
| 5 | ANOVA | 0.9946 | <.0001 | 1 vs. 0 | 0.0007 | p<0.05 |
|  |  |  |  | 2 vs. 0 | 0.0005 | p<0.05 |
|  |  |  |  | 3 vs. 0 | 0.0053 | p<0.05 |
| 6 | ANOVA | 0.7775 | <.0001 | 1 vs. 0 | <.0001 | p<0.05 |
|  |  |  |  | 2 vs. 0 | <.0001 | p<0.05 |
|  |  |  |  | 3 vs. 0 | <.0001 | p<0.05 |
|  |  |  |  | 4 vs. 0 | 0.0038 | p<0.05 |
| 7 | ANOVA | 0.7180 | <.0001 | 1 vs. 0 | <.0001 | p<0.05 |
|  |  |  |  | 2 vs. 0 | <.0001 | p<0.05 |
|  |  |  |  | 3 vs. 0 | <.0001 | p<0.05 |
|  |  |  |  | 4 vs. 0 | 0.0017 | p<0.05 |
| 8 | ANOVA | 0.1987 | <.0001 | 1 vs. 0 | <.0001 | p<0.05 |
|  |  |  |  | 2 vs. 0 | <.0001 | p<0.05 |
|  |  |  |  | 3 vs. 0 | 0.0006 | p<0.05 |
|  |  |  |  | 4 vs. 0 | 0.0142 | p<0.05 |
| 9 | ANOVA | 0.5869 | <.0001 | 1 vs. 0 | <.0001 | p<0.05 |
|  |  |  |  | 2 vs. 0 | <.0001 | p<0.05 |
|  |  |  |  | 3 vs. 0 | 0.0036 | p<0.05 |
|  |  |  |  | 4 vs. 0 | 0.0091 | p<0.05 |
| -3 | ANOVA | 0.5464 | 0.3434 | 1 vs. 0 | 0.0056 | p<0.05 |
| -4 | ANOVA | 0.3845 | 0.3165 | 1 vs. 0 | 0.0244 | p<0.05 |
| 10 | ANOVA | 0.4843 | <.0001 | 1 vs. 0 | <.0001 | p<0.05 |

| **Day** | **Test** | **Normality p** | **Main p** | **Comparison** | **Pairwise p** | **Significance** |
| --- | --- | --- | --- | --- | --- | --- |
|  |  |  |  | 2 vs. 0 | <.0001 | p<0.05 |
|  |  |  |  | 3 vs. 0 | 0.0002 | p<0.05 |
|  |  |  |  | 4 vs. 0 | 0.0004 | p<0.05 |
| 11 | ANOVA | 0.9862 | <.0001 | 1 vs. 0 | <.0001 | p<0.05 |
|  |  |  |  | 2 vs. 0 | <.0001 | p<0.05 |
|  |  |  |  | 3 vs. 0 | <.0001 | p<0.05 |
|  |  |  |  | 4 vs. 0 | 0.0001 | p<0.05 |
| 12 | ANOVA | 0.8425 | <.0001 | 1 vs. 0 | <.0001 | p<0.05 |
|  |  |  |  | 2 vs. 0 | <.0001 | p<0.05 |
|  |  |  |  | 3 vs. 0 | <.0001 | p<0.05 |
|  |  |  |  | 4 vs. 0 | <.0001 | p<0.05 |
| 13 | ANOVA | 0.8422 | <.0001 | 1 vs. 0 | <.0001 | p<0.05 |
|  |  |  |  | 2 vs. 0 | <.0001 | p<0.05 |
|  |  |  |  | 3 vs. 0 | <.0001 | p<0.05 |
|  |  |  |  | 4 vs. 0 | <.0001 | p<0.05 |
| 14 | ANOVA | 0.2515 | <.0001 | 1 vs. 0 | <.0001 | p<0.05 |
|  |  |  |  | 2 vs. 0 | <.0001 | p<0.05 |
|  |  |  |  | 3 vs. 0 | 0.0009 | p<0.05 |
|  |  |  |  | 4 vs. 0 | 0.0012 | p<0.05 |
| 15 | ANOVA | 0.4694 | <.0001 | 1 vs. 0 | <.0001 | p<0.05 |
|  |  |  |  | 2 vs. 0 | <.0001 | p<0.05 |
|  |  |  |  | 3 vs. 0 | 0.0004 | p<0.05 |
|  |  |  |  | 4 vs. 0 | 0.0006 | p<0.05 |
| 16 | ANOVA | 0.5671 | <.0001 | 1 vs. 0 | 0.0006 | p<0.05 |
|  |  |  |  | 2 vs. 0 | <.0001 | p<0.05 |
|  |  |  |  | 3 vs. 0 | 0.0007 | p<0.05 |

| **Day** | **Test** | **Normality p** | **Main p** | **Comparison** | **Pairwise p** | **Significance** |
| --- | --- | --- | --- | --- | --- | --- |
|  |  |  |  | 4 vs. 0 | 0.0008 | p<0.05 |
| 17 | ANOVA | 0.8595 | <.0001 | 1 vs. 0 | 0.0005 | p<0.05 |
|  |  |  |  | 2 vs. 0 | 0.0003 | p<0.05 |
|  |  |  |  | 3 vs. 0 | 0.0009 | p<0.05 |
|  |  |  |  | 4 vs. 0 | 0.0007 | p<0.05 |
| 18 | ANOVA | 0.9191 | <.0001 | 1 vs. 0 | 0.0007 | p<0.05 |
|  |  |  |  | 2 vs. 0 | 0.0002 | p<0.05 |
|  |  |  |  | 3 vs. 0 | 0.0018 | p<0.05 |
|  |  |  |  | 4 vs. 0 | 0.0014 | p<0.05 |
| 19 | ANOVA | 0.2933 | <.0001 | 1 vs. 0 | <.0001 | p<0.05 |
|  |  |  |  | 2 vs. 0 | <.0001 | p<0.05 |
|  |  |  |  | 3 vs. 0 | <.0001 | p<0.05 |
|  |  |  |  | 4 vs. 0 | <.0001 | p<0.05 |
|  |  |  |  | 6 vs. 0 | 0.0145 | p<0.05 |
|  |  |  |  | 7 vs. 0 | 0.0425 | p<0.05 |
| 20 | ANOVA | 0.2090 | <.0001 | 1 vs. 0 | <.0001 | p<0.05 |
|  |  |  |  | 2 vs. 0 | <.0001 | p<0.05 |
|  |  |  |  | 3 vs. 0 | 0.0002 | p<0.05 |
|  |  |  |  | 4 vs. 0 | <.0001 | p<0.05 |
|  |  |  |  | 5 vs. 0 | 0.0314 | p<0.05 |
|  |  |  |  | 6 vs. 0 | 0.0109 | p<0.05 |
| 21 | ANOVA | 0.4133 | <.0001 | 1 vs. 0 | <.0001 | p<0.05 |
|  |  |  |  | 2 vs. 0 | <.0001 | p<0.05 |
|  |  |  |  | 3 vs. 0 | 0.0002 | p<0.05 |
|  |  |  |  | 4 vs. 0 | <.0001 | p<0.05 |
|  |  |  |  | 5 vs. 0 | 0.0446 | p<0.05 |

| **Day** | **Test** | **Normality p** | **Main p** | **Comparison** | **Pairwise p** | **Significance** |
| --- | --- | --- | --- | --- | --- | --- |
|  |  |  |  | 6 vs. 0 | 0.0277 | p<0.05 |
| 22 | ANOVA | 0.6050 | 0.0002 | 1 vs. 0 | 0.0004 | p<0.05 |
|  |  |  |  | 2 vs. 0 | <.0001 | p<0.05 |
|  |  |  |  | 3 vs. 0 | 0.0004 | p<0.05 |
|  |  |  |  | 4 vs. 0 | <.0001 | p<0.05 |
|  |  |  |  | 5 vs. 0 | 0.0243 | p<0.05 |
|  |  |  |  | 6 vs. 0 | 0.0103 | p<0.05 |
|  |  |  |  | 7 vs. 0 | 0.0280 | p<0.05 |
| 23 | ANOVA | 0.6294 | <.0001 | 1 vs. 0 | 0.0008 | p<0.05 |
|  |  |  |  | 2 vs. 0 | <.0001 | p<0.05 |
|  |  |  |  | 3 vs. 0 | 0.0007 | p<0.05 |
|  |  |  |  | 4 vs. 0 | 0.0002 | p<0.05 |
|  |  |  |  | 6 vs. 0 | 0.0229 | p<0.05 |
| 24 | ANOVA | 0.6168 | <.0001 | 1 vs. 0 | <.0001 | p<0.05 |
|  |  |  |  | 2 vs. 0 | <.0001 | p<0.05 |
|  |  |  |  | 3 vs. 0 | <.0001 | p<0.05 |
|  |  |  |  | 4 vs. 0 | <.0001 | p<0.05 |
|  |  |  |  | 5 vs. 0 | 0.0065 | p<0.05 |
|  |  |  |  | 6 vs. 0 | 0.0026 | p<0.05 |
|  |  |  |  | 7 vs. 0 | 0.0090 | p<0.05 |
| 25 | ANOVA | 0.4252 | 0.0002 | 1 vs. 0 | 0.0010 | p<0.05 |
|  |  |  |  | 2 vs. 0 | 0.0002 | p<0.05 |
|  |  |  |  | 3 vs. 0 | 0.0011 | p<0.05 |
|  |  |  |  | 4 vs. 0 | 0.0003 | p<0.05 |
|  |  |  |  | 5 vs. 0 | 0.0387 | p<0.05 |
|  |  |  |  | 6 vs. 0 | 0.0266 | p<0.05 |

| **Day** | **Test** | **Normality p** | **Main p** | **Comparison** | **Pairwise p** | **Significance** |
| --- | --- | --- | --- | --- | --- | --- |
| 26 | ANOVA | 0.3778 | 0.0011 | 1 vs. 0 | 0.0017 | p<0.05 |
|  |  |  |  | 2 vs. 0 | 0.0002 | p<0.05 |
|  |  |  |  | 3 vs. 0 | 0.0006 | p<0.05 |
|  |  |  |  | 4 vs. 0 | 0.0002 | p<0.05 |
|  |  |  |  | 5 vs. 0 | 0.0161 | p<0.05 |
|  |  |  |  | 6 vs. 0 | 0.0105 | p<0.05 |
|  |  |  |  | 7 vs. 0 | 0.0391 | p<0.05 |
| 27 | ANOVA | 0.1199 | 0.0017 | 1 vs. 0 | 0.0015 | p<0.05 |
|  |  |  |  | 2 vs. 0 | 0.0003 | p<0.05 |
|  |  |  |  | 3 vs. 0 | 0.0008 | p<0.05 |
|  |  |  |  | 4 vs. 0 | 0.0005 | p<0.05 |
|  |  |  |  | 5 vs. 0 | 0.0338 | p<0.05 |
|  |  |  |  | 6 vs. 0 | 0.0157 | p<0.05 |
|  |  |  |  | 7 vs. 0 | 0.0384 | p<0.05 |
| 28 | ANOVA | 0.7648 | 0.0028 | 1 vs. 0 | 0.0021 | p<0.05 |
|  |  |  |  | 2 vs. 0 | 0.0005 | p<0.05 |
|  |  |  |  | 3 vs. 0 | 0.0016 | p<0.05 |
|  |  |  |  | 4 vs. 0 | 0.0009 | p<0.05 |
|  |  |  |  | 5 vs. 0 | 0.0345 | p<0.05 |
|  |  |  |  | 6 vs. 0 | 0.0276 | p<0.05 |
| 29 | ANOVA | 0.2151 | 0.0071 | 1 vs. 0 | 0.0050 | p<0.05 |
|  |  |  |  | 2 vs. 0 | 0.0015 | p<0.05 |
|  |  |  |  | 3 vs. 0 | 0.0028 | p<0.05 |
|  |  |  |  | 4 vs. 0 | 0.0019 | p<0.05 |
|  |  |  |  | 5 vs. 0 | 0.0392 | p<0.05 |
| 30 | ANOVA | 0.3459 | 0.0076 | 1 vs. 0 | 0.0023 | p<0.05 |

| **Day** | **Test** | **Normality p** | **Main p** | **Comparison** | **Pairwise p** | **Significance** |
| --- | --- | --- | --- | --- | --- | --- |
|  |  |  |  | 2 vs. 0 | 0.0006 | p<0.05 |
|  |  |  |  | 3 vs. 0 | 0.0016 | p<0.05 |
|  |  |  |  | 4 vs. 0 | 0.0009 | p<0.05 |
|  |  |  |  | 5 vs. 0 | 0.0149 | p<0.05 |
|  |  |  |  | 6 vs. 0 | 0.0415 | p<0.05 |
|  |  |  |  | 7 vs. 0 | 0.0472 | p<0.05 |
| 31 | ANOVA | 0.6910 | 0.0098 | 1 vs. 0 | 0.0043 | p<0.05 |
|  |  |  |  | 2 vs. 0 | 0.0012 | p<0.05 |
|  |  |  |  | 3 vs. 0 | 0.0040 | p<0.05 |
|  |  |  |  | 4 vs. 0 | 0.0028 | p<0.05 |
|  |  |  |  | 5 vs. 0 | 0.0383 | p<0.05 |
| 32 | ANOVA | 0.5334 | 0.0354 | 1 vs. 0 | 0.0182 | p<0.05 |
|  |  |  |  | 2 vs. 0 | 0.0036 | p<0.05 |
|  |  |  |  | 3 vs. 0 | 0.0112 | p<0.05 |
|  |  |  |  | 4 vs. 0 | 0.0057 | p<0.05 |
| 33 | ANOVA | 0.4240 | 0.0560 | 1 vs. 0 | 0.0112 | p<0.05 |
|  |  |  |  | 2 vs. 0 | 0.0035 | p<0.05 |
|  |  |  |  | 3 vs. 0 | 0.0106 | p<0.05 |
|  |  |  |  | 4 vs. 0 | 0.0033 | p<0.05 |
|  |  |  |  | 5 vs. 0 | 0.0269 | p<0.05 |
| 34 | ANOVA | 0.3772 | 0.1261 | 1 vs. 0 | 0.0320 | p<0.05 |
|  |  |  |  | 2 vs. 0 | 0.0132 | p<0.05 |
|  |  |  |  | 3 vs. 0 | 0.0346 | p<0.05 |
|  |  |  |  | 4 vs. 0 | 0.0106 | p<0.05 |
| 35 | ANOVA | 0.5830 | 0.0762 | 1 vs. 0 | 0.0149 | p<0.05 |
|  |  |  |  | 2 vs. 0 | 0.0043 | p<0.05 |

| **Day** | **Test** | **Normality p** | **Main p** | **Comparison** | **Pairwise p** | **Significance** |
| --- | --- | --- | --- | --- | --- | --- |
|  |  |  |  | 3 vs. 0 | 0.0123 | p<0.05 |
|  |  |  |  | 4 vs. 0 | 0.0047 | p<0.05 |
|  |  |  |  | 5 vs. 0 | 0.0332 | p<0.05 |
| 36 | ANOVA | 0.4360 | 0.0809 | 1 vs. 0 | 0.0339 | p<0.05 |
|  |  |  |  | 2 vs. 0 | 0.0048 | p<0.05 |
|  |  |  |  | 3 vs. 0 | 0.0111 | p<0.05 |
|  |  |  |  | 4 vs. 0 | 0.0064 | p<0.05 |
| 37 | ANOVA | 0.5559 | 0.1246 | 1 vs. 0 | 0.0396 | p<0.05 |
|  |  |  |  | 2 vs. 0 | 0.0095 | p<0.05 |
|  |  |  |  | 3 vs. 0 | 0.0205 | p<0.05 |
|  |  |  |  | 4 vs. 0 | 0.0084 | p<0.05 |
| 39 | ANOVA | 0.4045 | 0.4513 | 2 vs. 0 | 0.0474 | p<0.05 |
|  |  |  |  | 4 vs. 0 | 0.0390 | p<0.05 |
| 40 | ANOVA | 0.3661 | 0.5370 | 2 vs. 0 | 0.0419 | p<0.05 |
|  |  |  |  | 4 vs. 0 | 0.0476 | p<0.05 |
| 42 | ANOVA | 0.1796 | 0.4487 | 2 vs. 0 | 0.0452 | p<0.05 |
|  |  |  |  | 4 vs. 0 | 0.0488 | p<0.05 |
| 43 | ANOVA | 0.3659 | 0.3904 | 2 vs. 0 | 0.0401 | p<0.05 |
|  |  |  |  | 4 vs. 0 | 0.0480 | p<0.05 |
| 45 | ANOVA | 0.1713 | 0.0018 | 1 vs. 0 | 0.0012 | p<0.05 |
|  |  |  |  | 2 vs. 0 | 0.0001 | p<0.05 |
|  |  |  |  | 3 vs. 0 | 0.0006 | p<0.05 |
|  |  |  |  | 4 vs. 0 | 0.0001 | p<0.05 |
